# Finding Hadamard Matrices by a Quantum Annealing Machine

**DOI:** 10.1038/s41598-019-50473-w

**Published:** 2019-10-07

**Authors:** Andriyan Bayu Suksmono, Yuichiro Minato

**Affiliations:** 1 School of Electrical Engineering and Informatics, Institut Teknologi Bandung, Jl. Ganesha No.10, Bandung, Indonesia; 2 MDR Inc., Hongo 2-40-14-3F, Bunkyo-ku, Tokyo, Japan

**Keywords:** Information theory and computation, Quantum information

## Abstract

Finding a Hadamard matrix (H-matrix) among the set of all binary matrices of corresponding order is a hard problem, which potentially can be solved by quantum computing. We propose a method to formulate the Hamiltonian of finding H-matrix problem and address its implementation limitation on existing quantum annealing machine (QAM) that allows up to quadratic terms, whereas the problem naturally introduces higher order ones. For an *M*-order H-matrix, such a limitation increases the number of variables from *M*^2^ to (*M*^3^ + *M*^2^ − *M*)/2, which makes the formulation of the Hamiltonian too exhaustive to do by hand. We use symbolic computing techniques to manage this problem. Three related cases are discussed: (1) finding *N* < *M* orthogonal binary vectors, (2) finding *M*-orthogonal binary vectors, which is equivalent to finding a H-matrix, and (3) finding *N*-deleted vectors of an *M*-order H-matrix. Solutions of the problems by a 2-body simulated annealing software and by an actual quantum annealing hardware are also discussed.

## Introduction

Solving a hard problem is one of the most important issues in computational science. This kind of problem is characterized by its complexity; i.e. the required number of computing resource for doing the computation, which grows beyond polynomial against the input’s size. Researchers have put a lot of effort to solve such a problem, among others by employing quantum mechanics in the machinery of the computation process.

In a microscopic level, nature works under quantum mechanical principles that is hardly possible to simulate by classical computing machines^1^. This phenomenon drives the progress of quantum computing, both on the theory at the beginning^2,3^ and then is followed by the implementation of the quantum computer itself^4,5^. At present, a few kinds of early quantum computer models have been proposed and built, which mainly can be categorized into either a quantum gate model or a quantum annealing processor. Accordingly, we will refer a quantum computing machine either a QGM (Quantum Gate Machine) or a QAM (Quantum Annealing Machine), respectively.

In this paper, we address a problem of finding a Hadamard matrix; denoted by *H-SEARCH*, and its related problems, especially the formulation of their Hamiltonians for implementation on a QAM and experimenting with them using both of a simulator and a real-world quantum annealer. Previously^6,7^, we have suggested that finding a Hadamard matrix (H-matrix) among the set of all possible binary matrices of corresponding order, i.e. the H-SEARCH, is a hard problem. First proposed by Sylvester^8^ and then further developed by Hadamard^9^, an *M*-order H-matrix can be defined as an orthogonal binary $\{\,-\,1,\,+\,1\}$ matrix of size $M\times M$, where $M=1,2,4,8,\ldots ,4p,\ldots$^10,11^. The H-matrix is an important discrete structure in scientific fields and engineering applications^12,13^. Construction of a 2^*n*^ order H-matrix, for any positive integer *n*, can be done easily by using Sylvester’s method. Several construction methods for other values that do not follow $M={2}^{n}$ rule also have been proposed^14–17^. Nevertheless, there is no general method for constructing (nor finding) a 4*p* order H-matrix which can be applied to every positive integer *p*. Although no proof yet exists, it is conjectured^18,19^ that there is a H-matrix of order 4*p* for every positive integer *p*.

Existing Hadamard matrix construction techniques, including the Sylvester’s and other’s^14–17^, can be considered as *deterministic* methods. Although they capable to construct high order H-matrices for some particular orders, at the time of this writing, no method is able to construct H-matrix of order 668 (and several other orders) or knowing that this H-matrix is actually exists. We have formulated a tentative method that can be categorized as a *probabilistic* one, which is based on the SA (simulated annealing)^20–22^ and later on SQA (simulated quantum annealing)^23–25^. We have successfully found some low-order H-matrices that cannot trivially be constructed by the Sylvester method, either by SA^6^ or the SQA^7^. However, direct implementation of the method on existing QAM is hindered by unrealizable absolute terms in the energy function (Hamiltonian). Changing the absolute terms into their equivalent square terms will generate quartic terms, whereas existing QAM only allows up to quadratic terms to be implemented. A possible solution is by transforming the energy function containing high order terms into ones with up to two-body interaction terms using Boolean reduction^26,27^. In our case of H-SEARCH problem, however, it involves a large number of terms where the mathematical manipulation by hand is not an easy task.

In this paper, we also extend the H-SEARCH into a problem of finding a set of $N < M$ orthogonal (ortho-set) of binary vectors. Along with H-SEARCH, which is equivalent to finding *M* ortho-set of *M*-order binary vectors, we also address H-matrix completion problem of finding *N*-deleted vectors of a given *M*-order H-matrix. The large number of terms in the Hamiltonian of these problems requires both a systematic and automated solution. We propose a method to systematically perform Boolean reduction on a large number of terms and encourage the usage of symbolic computation to formulate the energy function which leads to the Hamiltonian of the problems. We present some examples of finding low-order H-matrices to clarify the proposed method. Additionally, we use D-Wave *neal* package to find the solutions of the formulated 2-body interacting Hamiltonian of the problems by using simulated annealing and by implementing on an actual quantum annealer by using the D-Wave’s DW2000Q quantum processor. Although at this time only low order H-SEARCH problem can be implemented, due to the limited numbers of qubits in present day quantum annealer, we expect that with the grows of the number of qubits and the improvement of the method in forthcoming years, the proposed method can be used to benchmark newly released quantum annealers.

## Methods

### Quantum annealing machines

We refer a QAM or an adiabatic quantum computing machine as a configurable or a programmable quantum Ising systems ${\hat{H}}_{pot}$, whose transverse magnetic field ${\hat{H}}_{kin}$ can be controlled and the state of its spins can be read individually upon completion of an evolution. The Hamiltonians of such a system; for a given spin configuration $\{{\hat{\sigma }}_{k}^{\alpha }\}\equiv \hat{\sigma }$; where $\alpha \in \{x,y,z\}$, $k\in K=\{1,2,\ldots ,i,j,\ldots \}$ is the set of lattice’s indices, is given by

1 $${\hat{H}}_{pot}(\hat{\sigma })\equiv -\,\sum _{i\ne j}\,{J}_{ij}{\hat{\sigma }}_{i}^{z}{\hat{\sigma }}_{j}^{z}-\sum _{i}\,{h}_{i}{\hat{\sigma }}_{i}^{z}$$

and,

2 $${\hat{H}}_{kin}(\hat{\sigma })\equiv -\,\Gamma \,\sum _{i}\,{\hat{\sigma }}_{i}^{x}$$

where ${J}_{ij}$ is a coupling constant or interaction strength between a spin at site *i* with a spin at site *j*, *h*_*j*_ is magnetic strength at site *j*, and $\{{\hat{\sigma }}_{i}^{z},{\hat{\sigma }}_{i}^{x}\}$ are Pauli’s matrices at site-*i*. In QA (Quantum Annealing)^23–25,28–37^, quantum fluctuation is elaborated by introducing a transverse magnetic field $\Gamma$. To solve a problem by using QAM, we have to encode the variables into spins with their corresponding Ising coefficients $\{{h}_{i},{J}_{ij}\}$. Then it is executed by the following quantum adiabatic evolution

3 $${\hat{H}}_{QA}(\hat{\sigma },t)=(1-\frac{t}{\tau }){\hat{H}}_{kin}(\hat{\sigma })+\frac{t}{\tau }{\hat{H}}_{pot}(\hat{\sigma })$$

where $t\in [0,\tau ]$. By keeping the system in an adiabatic condition during the process, the ground-state at the end of the evolution of the system will represent a solution of the problem.

In a real quantum annealing device, the presence of thermal noise cannot be avoided. However, Dickson *et al*.^38^ show that such a noise plays a positive role in increasing the robustness of a quantum annealing device. They have shown by theory and demonstrated by experiments that due to the noise, the probabilities to perform a successful computation by the device with annealing time several orders of magnitude longer than its coherence time is comparable to a system with a fully coherent one.

We can see from Eq. (1) that the Hamiltonian includes up to quadratic terms, so that in principle it only allows encoding of quadratic (binary) problems. When the problem contains higher order terms (than the quadratic), we have to find a way to convert it into expressions that only include up to quadratic. Additionally, since the number of the spins/qubits are related to the number of binary variables, it further constraints the size of the problem that can be managed and therefore limits the machine’s capability.

Some efforts to implement the QAM have been initiated, among others is the construction of quantum annealer where the qubits are manufactured as superconducting quantum devices called RF-SQUID (Radio Frequency-Super Conducting Quantum Interference Device)^5^. The scalability of the device makes it possible for the number of qubits grows very rapidly; the last generation at the time of this writing achieves more than 2000. This device has been applied to solve various kinds of problems, such as, prime factorization^39^, hand written digit recognition^40^, computational biology^41^, and hydrologic inverse analysis^42^.

### Energy minimization and hamiltonian formulation

Consider two kinds of binary variables, i.e. a spin variable $s\in \{\,-\,1,\,+\,1\}$ and a Boolean variable $q\in \{0,1\}$, which are related by the following transform

4 $$s=\frac{1}{2}(1-q)$$

5 $$q=(1-2s).$$

To denote the location of the variables (such as when they are elements of a vector or an array), an index will be inserted as a subscript. Therefore, at location *i* they will become *s*_*i*_ and *q*_*i*_, respectively.

Consider an $M\times M$ binary matrix, whose elements are represented by spin variables *s*_*i*_ as follows

6 $$(\begin{array}{cccc}{s}_{0} & {s}_{M} & \cdots  & {s}_{M(M-1)}\\ {s}_{1} & {s}_{M+1} & \cdots  & {s}_{M(M-1)+1}\\ \cdots  & \cdots  & \cdots  & \cdots \\ {s}_{M-1} & {s}_{2M-1} & \cdots  & {s}_{{M}^{2}-1}\end{array})$$

We can show (see Supplementary Information-I) that the orthogonality condition of the matrix, i.e., any pair of either columns or row vectors are orthogonal, will be achieved when its related energy function defined by

7 $${E}_{k}(s)={E}_{4}(s)=\frac{{M}^{2}(M-1)}{2}+2\,\sum _{i < j < m < n < {M}^{2}-1}\,{s}_{i}{s}_{j}{s}_{m}{s}_{n}$$

is minimized. Note that the subscript *k* in ${E}_{k}(s)$ refers to *k*-body interaction formulated in this expression. Since there are 4-body interactions in this expression, it also can be written as ${E}_{4}(s)$.

Since QAM can only deals with up to quadratic terms, whereas Eq. (7) consists of quartics, we have to perform Boolean reduction. The reduction can be done by the following substitution^27^

8 $${q}_{i}{q}_{j}\leftarrow {q}_{k}+{C}_{\wedge }({q}_{i},{q}_{j},{q}_{k};{\delta }_{i,j})$$

where the compensation term ${C}_{\wedge }({q}_{i},{q}_{j},{q}_{k};{\delta }_{i,j})$ is given by

9 $${C}_{\wedge }({q}_{i},{q}_{j},{q}_{k};{\delta }_{i,j})={\delta }_{i,j}(3{q}_{k}+{q}_{i}{q}_{j}-2{q}_{i}{q}_{k}-2{q}_{j}{q}_{k})$$

According to these formulas, the Boolean reduction should be done for expressions in *q* variables, whereas the problems are originally formulated in *s*. Therefore, the reduction should be conducted in several steps, beginning from the *k*-body energy function expressed in *s* variables up to obtaining a 2-body (quadratic) expression of the problem’s Hamiltonian. These steps are given by the following *construction diagram*

10 $${E}_{k}(s)\to {E}_{k}(q)\to {E}_{2}(q)\to {E}_{2}(s)\to {\hat{H}}_{2}(\hat{\sigma })$$

We can show (see Supplementary Information-I for the detail) that transforming a *k*-body energy function into its corresponding 2-body’s increases the number of variables (required qubits) from *M*^2^ to $({M}^{3}+{M}^{2}-M)/2$. Therefore, Hamiltonian formulation of H-SEARCH problem involved many variables which is not easy to be manipulated by hand. We can employ symbolic computing to perform this task. Based on the construction diagram and by employing symbolic computation, the calculation of the 2-body energy function can be formulated by Algorithm 1.

After obtaining the 2-body energy function ${E}_{2}(s)$, the Hamiltonian ${\hat{H}}_{2}$ can be obtained by replacing all of the binary variables at location *i*, i.e. *s*_*i*_, with the corresponding qubit’s spin ${\hat{\sigma }}_{i}^{z}$. For examples, applying the algorithm to the simplest problem of order-2, we obtain the following results

11 $$\begin{array}{rcl}{\hat{H}}_{2}(\hat{\sigma }) & = & 28+6{\hat{\sigma }}_{0}^{z}+6{\hat{\sigma }}_{1}^{z}+6{\hat{\sigma }}_{2}^{z}+6{\hat{\sigma }}_{3}^{z}-12{\hat{\sigma }}_{4}^{z}-12{\hat{\sigma }}_{5}^{z}\\  &  & +\,2{\hat{\sigma }}_{0}^{z}{\hat{\sigma }}_{1}^{z}+2{\hat{\sigma }}_{0}^{z}{\hat{\sigma }}_{2}^{z}+2{\hat{\sigma }}_{0}^{z}{\hat{\sigma }}_{3}^{z}-8{\hat{\sigma }}_{0}^{z}{\hat{\sigma }}_{4}^{z}-4{\hat{\sigma }}_{0}^{z}{\hat{\sigma }}_{5}^{z}\\  &  & +\,2{\hat{\sigma }}_{1}^{z}{\hat{\sigma }}_{2}^{z}+4{\hat{\sigma }}_{1}^{z}{\hat{\sigma }}_{3}^{z}-4{\hat{\sigma }}_{1}^{z}{\hat{\sigma }}_{4}^{z}-8{\hat{\sigma }}_{1}^{z}{\hat{\sigma }}_{5}^{z}+2{\hat{\sigma }}_{2}^{z}{\hat{\sigma }}_{3}^{z}\\  &  & -\,8{\hat{\sigma }}_{2}^{z}{\hat{\sigma }}_{4}^{z}-4{\hat{\sigma }}_{2}^{z}{\hat{\sigma }}_{5}^{z}-4{\hat{\sigma }}_{3}^{z}{\hat{\sigma }}_{4}^{z}-8{\hat{\sigma }}_{3}^{z}{\hat{\sigma }}_{5}^{z}+8{\hat{\sigma }}_{4}^{z}{\hat{\sigma }}_{5}^{z}\end{array}$$

which consists of 22-terms. The next 4-order H-matrix searching problem Hamiltonian will consists of 389 expression, which is given as follows

12 $${\hat{H}}_{2}(\hat{\sigma })=1,248+66{\hat{\sigma }}_{0}^{z}+\cdots -44{\hat{\sigma }}_{39}^{z}+6{\hat{\sigma }}_{0}^{z}{\hat{\sigma }}_{1}^{z}+\cdots +8{\hat{\sigma }}_{38}^{z}{\hat{\sigma }}_{39}^{z}$$

A complete expression of Eq. (12) can be found in the Supplementary Information-II.

**Algorithm 1:**
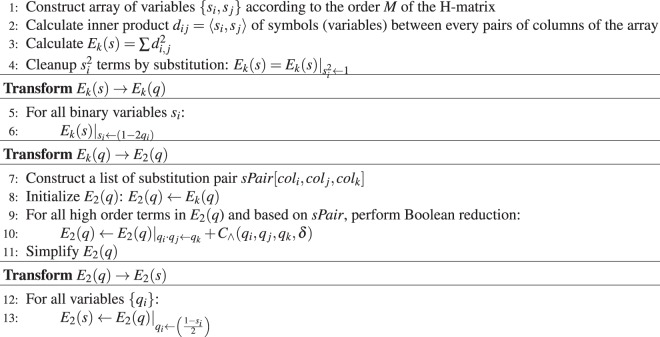
Construction of 2-body Energy Function of the H-SEARCH Problem.

### Related problems

The H-SEARCH problem can be considered as finding *M* orthogonal of *M* length binary vectors. In general, the problem of finding orthogonal binary vectors can be categorized as follows:

- *Problem-1*: Finding a set of $N < M$ orthogonal *M*-length binary vectors.
- *Problem-2*: Finding a set of $N=M$ orthogonal *M*-length binary vectors, which is equivalent to finding an *M*-order H-matrix.
- *Problem-3*: Finding $N < M$ missing columns of an *M*-order Hadamard matrix.

As an example of *Problem-1*, finding $N=3$ orthogonal vectors of length $M=4$ will have the following Hamiltonian,

13 $${\hat{H}}_{2}(\hat{\sigma })=768+52{\hat{\sigma }}_{0}^{z}+\cdots -52{\hat{\sigma }}_{23}^{z}+4{\hat{\sigma }}_{0}^{z}{\hat{\sigma }}_{1}^{z}+\cdots +8{\hat{\sigma }}_{22}^{z}{\hat{\sigma }}_{23}^{z}$$

Similarly, *Problem-2* of finding $N=2$ missing vectors of an $M=4$ order H-matrix, knowing the other 2 vectors are (+, +, +, −)^T^ and (+, −, +, −)^*T*^; note that we have represented 1 entries by + and the −1 by − for conciseness, will have the following Hamiltonian

14 $${\hat{H}}_{2}(\hat{\sigma })=128+14{\hat{\sigma }}_{0}^{z}+\cdots -28{\hat{\sigma }}_{11}^{z}+2{\hat{\sigma }}_{0}^{z}{\hat{\sigma }}_{1}^{z}+\cdots +8{\hat{\sigma }}_{10}^{z}{\hat{\sigma }}_{11}^{z}$$

The derivations of Eqs (13) and (14) are given in the Supplementary Information-I and their complete expressions are given in the Supplementary Information-II.

## Results

Experiments have been conducted to verify the proposed method by both of simulation and actual implementation on a quantum annealer. In the simulation, a python-based simulated annealing package, the D-Wave’s *neal*^43^, has been employed to find minimum energies and related configurations that yield solutions of the problem. Input of the simulator are Ising coefficients $\{{h}_{i},{J}_{ij}\}$ of the problem’s Hamiltonian or energy function. These coefficients can be extracted from either ${\hat{H}}_{2}(\hat{\sigma })$ or ${E}_{2}(s)$, where its constant value is omitted which translates into the shift of the ground state energy to a negative value of the corresponding constant. Then, we normalize the coefficients by dividing them by the largest absolute values of the coefficients to simulate a real QAM input parameters. We also have done some experiments on a D-Wave’s DW2000Q quantum annealer. The “programming” of this quantum computer is performed by configuring the qubits which are connected by a Chimera graph, and assigning weight on each of the qubit and strength of the coupler that connect the qubits according to the Ising coefficients. A simple Hamiltonian can be implemented directly by manual configuration, whereas a more complex one needs an embedding tool.

### Simulation on D-wave *neal* simulator

The input of the *neal* simulated annealing software are Ising coefficients, which after scaling will simulate the input of the D-Wave quantum annealer, except that it is not necessary to take care of the restriction of the connection among the qubits imposed by the processor’s graph. All of the Neal simulations have been conducted on an i7 Windows PC with 16G memory.

#### Finding 2-order and 4-order H-matrix

To solve the problem of finding 2-order H-matrix, we have used the Hamiltonian given by Eq. (11), which after normalization yields the following bias values

$$h=(0.5,0.5,0.5,0.5,-\,1.0,1.0)$$

whereas the coupling coefficients between a pair of qubits are given as follows

$$J=(\begin{array}{cccccc}\ast  & 0.167 & 0.333 & 0.167 & -\,0.667 & -\,0.333\\ \ast  & \ast  & 0.167 & 0.333 & -\,0.333 & -\,0.667\\ \ast  & \ast  & \ast  & 0.167 & -\,0.667 & -\,0.333\\ \ast  & \ast  & \ast  & \ast  & -\,0.333 & -\,0.667\\ \ast  & \ast  & \ast  & \ast  & \ast  & 0.667\\ \ast  & \ast  & \ast  & \ast  & \ast  & \ast \end{array}).$$

Since the diagonal entries are not used and *J* is symmetric, it is sufficient to only show the upper diagonal elements. We have set the number of sweeps in the simulator to 1,000 and the number of configurations to 10. The average running time for this simulation is around 2.6 ms. Table 1 displays the obtained configurations with their corresponding energy values after the simulation has been finished.

**Table 1:** Solutions of finding 2-order H-matrix problem by D-Wave’s *neal*.

No	Configuration	Energy	Ground-State
1	(+, −, +, **+, +, +**)	−2.33	Y
2	(+, −, −, −, **+**, **−**)	−2.33	Y
3	(+, −, +, **+, +, +**)	−2.33	Y
4	(−, +, +, +, **+, +**)	−2.33	Y
5	(+, −, +, +, **+, +**)	−2.33	Y
6	(+, −, −, −, **+, −**)	−2.33	Y
7	(+, −, +, −, **+, −**)	−2.00	N
8	(+, +, −, +, **+, +**)	−2.33	Y
9	(**+, −, +, −, +, −**)	−2.00	N
10	(**−, +, +, +, +, +**)	−2.33	Y

The final configurations of main variables and ancillas along with their associated energies are shown. The ground-state column indicates whether the lowest energy has been achieved, which is marked by “Y”, or has not been achieved which is marked by “N”. In the Configuration column, the last two qubits indicated by bold are ancillas.

Based on Eq. (11), we realized that the value of the constant is 28.00, whereas the largest (absolute value) of coefficients is 12.00. By normalization, the constant becomes 2.33, therefore the value of the lowest energy (the ground state) is −2.33, which is in agreement with the simulation result given by Table 1. We observed from the results that not all of the configurations achieved ground states. In the table, configurations achieving the ground states’s are marked by “Y”, whereas non-ground states are marked by “N”. The elements of the obtained H-matrices are given by the first 4 values of the configuration, such as (*s*_0_, *s*_1_, *s*_2_, *s*_3_) = (+, −, +, +) for the first configuration, whereas the corresponding ancillas (*s*_4_, *s*_5_) = (+, +) can be neglected. Reshaping the solution vectors into 2 × 2 matrices yields various orthogonal and non-orthogonal 2 × 2 matrices, displayed subsequently as follows,

15 $$(\begin{array}{cc}+ & +\\ - & +\end{array}),\,(\begin{array}{cc}+ & -\\ - & -\end{array}),\,\ldots ,\,(\begin{array}{cc}- & +\\ + & +\end{array})$$

It is easy to verify that the matrices that correspond to the ground state energy are indeed Hadamards.

In the second example, we consider the problem of finding 4-order H-matrix. By taking ${\delta }_{ij}=4\times {H}_{max}$, where *H*_*max*_ is the maximum absolute value of the elements of indicator matrix $D\equiv {H}^{T}H$, the problem’s Hamiltonian can be expressed by Eq. (12). By setting the simulation parameters as before, we found that the average running time for this simulation is around 21.1 ms and we obtained the following set of energies (written to the second decimal places)

$$\begin{array}{c}-\,16.00,-\,15.62,-\,15.62,-\,15.71,-\,15.71,\\ \,-\,15.71,-\,15.71,-\,15.71,-\,16.00,-\,15.62\end{array}$$

Our calculation shows that the ground state energy should have been −16.00, which only 2 out of 10 solutions have achieved. As an example, the first solution related to ${E}_{2}(s)=-\,16.00$ and the second one related to ${E}_{2}(s)=-\,15.62$ yields the following configurations

$$\begin{array}{l}(\,+\,,+\,,+\,,+\,,+\,,-\,,-\,,+\,,-\,,-\,,+\,,+\,,-\,,+\,,-\,,+\,,+\,,+\,,+\,,+\,,\\ +\,,+\,,+\,,+\,,+\,,+\,,+\,,+\,,+\,,-\,,+\,,+\,,+\,,+\,,-\,,+\,,-\,,+\,,+\,,+\,)\end{array}$$

and

$$\begin{array}{l}(\,-\,,+\,,+\,,+\,,-\,,-\,,+\,,-\,,-\,,-\,,-\,,-\,,-\,,+\,,-\,,+\,,-\,,+\,,+\,,+\,,\\ -\,,+\,,+\,,+\,,-\,,+\,,+\,,+\,,-\,,-\,,+\,,-\,,-\,,+\,,+\,,+\,,-\,,+\,,-\,,+\,)\end{array}$$

respectively. By taking the first 16 elements of the solution vectors and reshaping them into 4 × 4 matrices, we obtain the following results,

16 $$(\begin{array}{cccc}+ & + & + & +\\ + & - & - & +\\ - & - & + & +\\ - & + & - & +\end{array}),\,(\begin{array}{cccc}- & + & + & +\\ - & - & + & -\\ - & - & - & -\\ - & + & - & +\end{array})$$

We can verify that the first solution with ${E}_{2}(s)=-\,16.00$ is actually an orthogonal matrix, whereas the second one related to ${E}_{2}(s)=-\,15.62$ is not.

**Figure 1:**
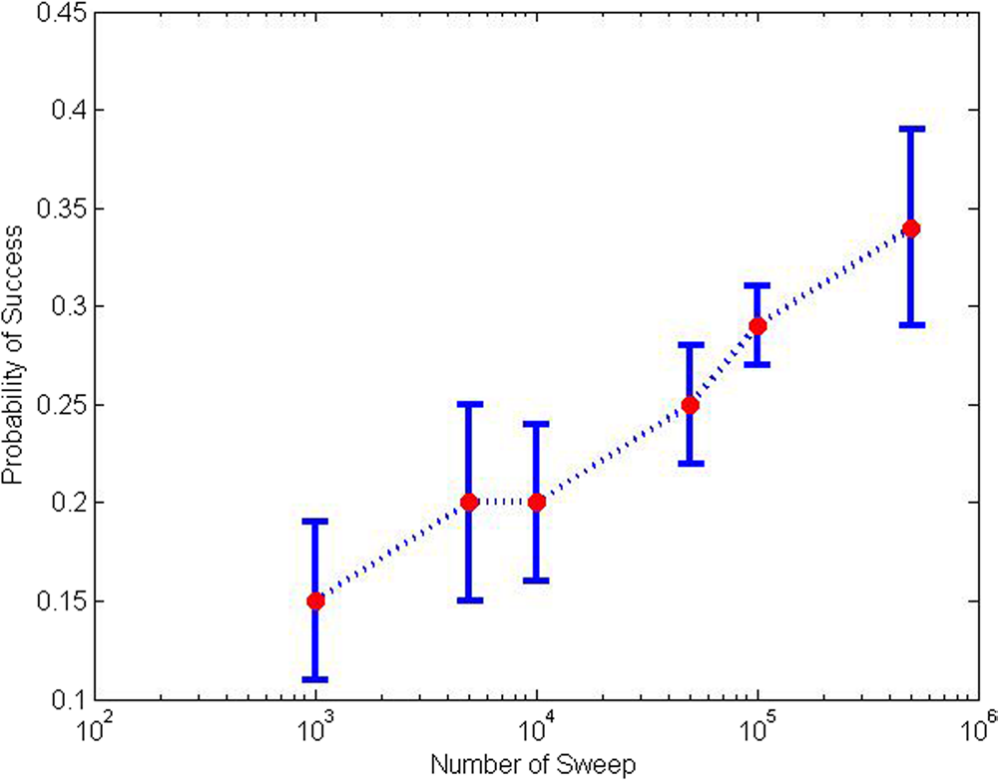
The probability of success in finding solution in *Neal* simulation for various sweep numbers. We observe that increasing the number of sweeps will increase the probability of success.

The probability of success in finding correct solutions can be improved by increasing the number of sweeps. Whereas the default number of sweeps of 1000 yields only about 20% correct solutions, our experiment by increasing the number of sweeps confirm such improvement. In this experiment, we have increased the number of reads from 10 to 100 so that the measurement of the probability can be made more precise, whereas the number of sweeps are increased subsequently into 5000, 10,000, 50,000, 100,000 and finally 500,000. Subsequent improvement of the probability of success plotted in a semi-logarithmic scale is shown in Fig. 1.

#### Finding a set of *N*-orthogonal *M*-order binary vectors

In this experiment, our objective is to find a set of 3-orthogonal binary vectors of length 12. The number of binary variables that are required to do this task are 72, whereas the number of ${E}_{2}(s)$ terms are 7,765. The Hamiltonian obtained from ${E}_{2}(s)$ after symbolic computation yields the following expression

17 $${\hat{H}}_{2}(\hat{\sigma })=19,\,872+404{\hat{\sigma }}_{0}^{z}+\cdots +404{\hat{\sigma }}_{71}^{z}+4{\hat{\sigma }}_{0}^{z}{\hat{\sigma }}_{1}^{z}+\cdots +8{\hat{\sigma }}_{70}^{z}{\hat{\sigma }}_{71}^{z}$$

A complete expression of the Hamiltonian can be found in Supplementary Information-II.

Based on ${E}_{2}(s)$ and by using compensation term ${\delta }_{ij}=5\times {M}^{2}$, the calculated ground-state energy is −49.19. Setting the number of sweep to 1000 as in the previous case did not give a correct solution, therefore, we increased the number of sweeps to 500,000 while keeping the number of configurations at 10. The average running time for this simulation is around 10.35 s. We obtained the energies at each of the configuration in the solutions as follows

$$\begin{array}{c}-\,49.09,-\,49.11,-\,49.09,-\,49.13,-\,49.17,\\ \,-\,49.11,-\,49.19,-\,49.19,-\,49.11,-\,49.11\end{array}$$

Especially, the solution given by the ground-state with energy at −49.19 are the following vectors

18 $$\begin{array}{rcl}{v}_{g,1} & = & {(+,-,-,-,+,+,-,-,+,-,+,-)}^{T}\\ {v}_{g,2} & = & {(-,-,-,-,-,-,+,-,+,-,-,+)}^{T}\\ {v}_{g,3} & = & {(+,+,-,-,-,-,-,-,-,+,+,+)}^{T}.\end{array}$$

where (·)^*T*^ denotes transpose. We can verify that these three binary vectors are orthogonal to each other. On the other hand, the non-ground state solutions with energy −49.09 given by the following vectors

19 $$\begin{array}{rcl}{v}_{ng,1} & = & {(+,-,-,+,-,+,-,-,+,+,+,+)}^{T}\\ {v}_{ng,2} & = & {(-,+,-,-,+,-,+,-,+,-,-,-)}^{T}\\ {v}_{ng,3} & = & {(+,+,+,+,-,-,-,+,+,+,-,-)}^{T}\end{array}$$

are not a set of 3-orthogonal binary vectors, and therefore is not a correct solution.

#### Finding a missing vector of a 12-order H-matrix

For the completion problem, we have chosen a 12-order H-matrix as a case, whose 1 column vector has been deleted. The rests of 11 known vectors are as follows,

20 $$\begin{array}{rcl}{v}_{0} & = & {(+,+,+,+,+,+,+,+,+,+,+,+)}^{T}\\ {v}_{1} & = & {(+,-,+,-,+,+,+,-,-,-,+,-)}^{T}\\ {v}_{2} & = & {(+,-,-,+,-,+,+,+,-,-,-,+)}^{T}\\ {v}_{3} & = & {(+,+,-,-,+,-,+,+,+,-,-,-)}^{T}\\ {v}_{4} & = & {(+,-,+,-,-,+,-,+,+,+,-,-)}^{T}\\ {v}_{5} & = & {(+,-,-,+,-,-,+,-,+,+,+,-)}^{T}\\ {v}_{6} & = & {(+,-,-,-,+,-,-,+,-,+,+,+)}^{T}\\ {v}_{7} & = & {(+,+,-,-,-,+,-,-,+,-,+,+)}^{T}\\ {v}_{8} & = & {(+,+,+,+,-,-,-,+,-,-,+,-)}^{T}\\ {v}_{9} & = & {(+,+,+,-,-,-,+,-,-,+,-,+)}^{T}\\ {v}_{10} & = & {(+,+,-,+,+,+,-,-,-,+,-,-)}^{T}\end{array}$$

Since all of the elements of *v*_0_ are 1, it is a seminormalized H-matrix. Our symbolic computation yields the number of terms in ${E}_{k}(s)$ is 379, ${E}_{k}(q)$ is 407, ${E}_{2}(q)$ is 407 and ${E}_{2}(s)$ is 379. The Hamiltonian obtained from ${E}_{2}(s)$, after symbolic computation, is as follows

21 $${\hat{H}}_{2}(\hat{\sigma })=756+2{\hat{\sigma }}_{0}^{z}{\hat{\sigma }}_{1}^{z}+\cdots +2{\hat{\sigma }}_{0}^{z}{\hat{\sigma }}_{27}^{z}+\cdots -2{\hat{\sigma }}_{26}^{z}{\hat{\sigma }}_{27}^{z}$$

The complete expression is provided in Supplementary Information-II.

By setting the number of sweeps to 1,000 in the simulation we obtained the energy equal to −66.00, which are identical for all of 10 configurations in the solution. This simulation was very fast so that the recorded running time for this simulation is less than 0.1 *μs*. The result shows that all of the configuration achieved lowest energy, which consist of two binary vectors as follows

22 $$\begin{array}{rcl}{v}_{11,1} & = & {(+,-,+,+,+,-,-,-,+,-,-,+)}^{T}\\ {v}_{11,2} & = & {(-,+,-,-,-,+,+,+,-,+,+,-)}^{T}\end{array}$$

By inspection, we can see that ${v}_{11,2}=-\,{v}_{11,1}$ and therefore both of them are correct solutions that completes the set of vectors given by Eq. (20) to become a 12-order H-matrix.

### Experiments on D-Wave quantum annealer

We have implemented the Hamiltonian of H-SEARCH problems (for order 2 and 4), finding a set of $N < M$ orthogonal binary vectors of order *M*, and H-matrix completion problems into DW2000Q quantum annealer. The DW2000Q has 2048 qubits and 6016 couplers, where the qubits are connected by a C16 Chimera graph, which means that its 2048 qubits are logically map into a 16 × 16 matrix of unit cell, whose each cell consists of 8 qubits^44^. The layout of the cell can be represented either by a *column* or by a *cross*. In this paper, we use the *cross* layout to show the connection among the qubits in each of the presented problem.

The schedule of quantum annealing process in DW2000Q can be adjusted by the user. However, in the following experiments, we have used the default schedule^45^; where the kinetic energy ${E}_{kin}/h$ (with *h* is the Planck constant) has been set at around 6 GHz at the beginning; which is decreased exponentially to around zero at the end of the annealing process. Meanwhile, the potential energy ${E}_{pot}/h$ is started from zero at the beginning and then increased exponentially to around 12 GHz at the end of the annealing. Connections among the qubits are set to follow the values of *J*_*ij*_ whereas the offset is set according to the values of *h*_*j*_ of related problem to solve. The run time of the device for all of the experiments are 20 *μs*. During the experiments, among the available 2048 qubits, only 2038 qubits are active but it will not affect the implementation since the required number is smaller than the number of active qubits, which are then chosen automatically by the embedding software.

#### Finding 2-order and 4-order H-matrix

**Figure 2:**
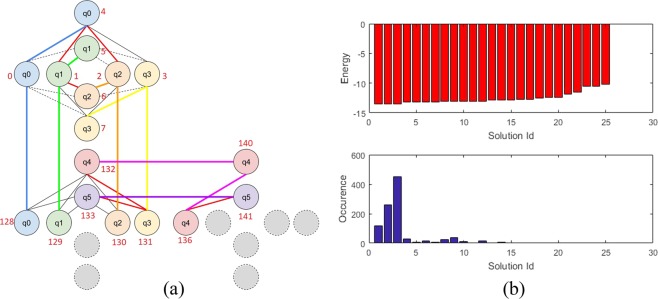
Implementation of finding a 2-order H-matrix problem’s Hamiltonian into a quantum annealer: (**a**) Embedding 6 logical qubits into 17 physical qubits in a Chimera graph’s of the DW2000Q. Circles with same color represent an identical logical qubit which are implemented on different physical qubits. As an example, the green-colored logical qubit *q*_1_ is represented by three different physical qubits no.1, 5, and 129, (**b**) Obtained results after quantum annealing: the distribution of energy related to each solution (top) and distribution of the solutions obtained by 1000 reads (bottom).

The Hamiltonian of the finding 2-order H-matrix problem given by Eq. (11) indicates that 6 (logical) qubits are required. However, implementation on the Chimera graph increases the number into 17 (physical) qubits which are located in the neighbouring blocks (unit cells). We have manually designed the qubit’s connection, whose configuration result is shown in Fig. 2(a).

We have used a default annealing schedule, whereas the number of reads is set to 1000. Energy distribution of the result and its related occurrence number of each solution are shown in the top and bottom parts of Fig. 2(b), respectively. We obtained a minimum energy of −13.52, which corresponds to solution vector (+, −, −, −) for the first four qubits, while the following values representing ancillas can be ignored. The solution can be rearranged into a 2 × 2 arrays as follows

23 $$(\begin{array}{cc}+ & -\\ - & -\end{array})$$

which actually is a 2-order H-matrix. We have observed that when non-ground state solution occurs, the obtained results will not be correct, i.e., they are not H-matrices. This experiment by setting 1000 sweeps produces 20% of error; it can be reduced by increasing the number of sweeps in the initialization of the Neal.

For the 4-order H-SEARCH problem, the Hamiltonian expressed in Eq. (12) indicates that 40 (logical) qubits are required. This number increases when it is implemented on the set of qubits with Chimera graph connection. We have employed SAPI (Solver Application Programming Interface) embedding tool^46^ which is provided by the D-Wave to construct the connection among the qubits automatically. After optimization, the SAPI indicates that 344 (physical) qubits are required.

**Figure 3:**
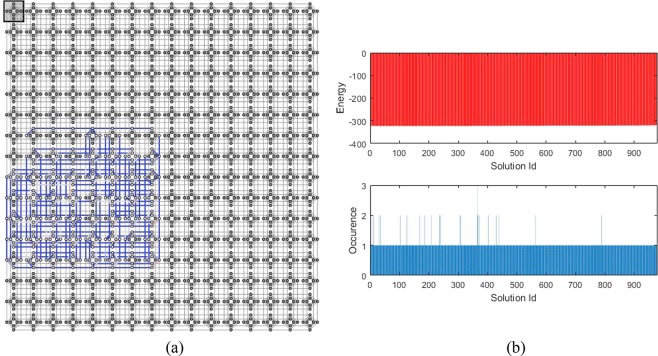
Implementation of finding a 4-order H-matrix problem’s Hamiltonian into a quantum annealer: (**a**) Embedding diagram of 40 logical qubits into 344 physical qubits in DW2000Q which is obtained by an embedding tool, (**b**) Results after finishing the quantum annealing evolution: the distribution of energy related to each solution (top) and distribution of the solutions obtained by 1000 reads (bottom).

Sketched of the qubits connection is displayed in Fig. 3(a), whereas the distribution of energy and its related population are depicted in top and bottom part of Fig. 3(b) respectively. Connection diagram displayed in Fig. 3(a) shows that a 4-order H-SEARCH problem already occupied a significant number of available qubits and couplers of the DW2000Q quantum processor. In contrast to the 2-order case, the figure shows an almost uniform distribution, except for a few number of solutions. In histogram of Fig. 3(b), the horizontal axis indicates the solution Id, in which the upper and lower histogram will have the same Id at the same coordinate location. The vertical axis indicates energy in the upper histogram, whereas it shows the occurrence at the bottom histogram, i.e., it shows the number of the solution for the corresponding Id with achieved energy level shown at the upper histogram. We have verified the received solutions from the D-Wave cloud that among 1000 reads, there are 977 distinct solutions where 38 are correct and 962 are wrong.

Default annealing schedule has been used and we also set the number of reads to 1000. The achieved lowest energy for the given configuration is −322.91. The corresponding solution, after neglecting the ancillas and reformatting it into a 4 × 4 matrix, is as follows

24 $$(\begin{array}{cccc}- & - & + & +\\ + & - & + & -\\ - & - & - & -\\ - & + & + & -\end{array})$$

We can verify that the solution is indeed a H-matrix, therefore the D-Wave has successfully found the H-matrix of order-4.

#### Finding a set of 3-orthogonal 12-order binary vectors

**Figure 4:**
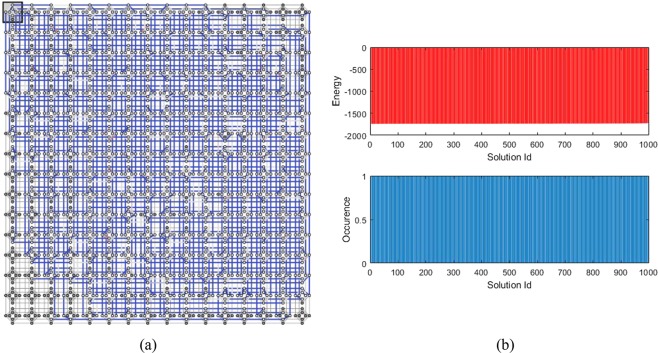
Realization of finding a set of 3-orthogonal binary vectors of order (length) 12 into a quantum annealer: (**a**) Embedding 72 logical qubits into 1,766 physical qubits in a Chimera-connected qubits of the DW2000Q, (**b**) Obtained results after quantum annealing: the distribution of energy related to each solution (top) and distribution of the solutions obtained by 1000 reads (bottom).

In this experiment, we configured the D-Wave to find a set of 3 orthogonal binary vectors of order 12. The Hamiltonian given by Eq. (17) indicates that 72 (logical) qubits is necessary. We also used SAPI embedding tool to configure the Chimera graph to obtain the qubits connection. After several steps of optimizations, the SAPI shows that 1,766 (physical) qubits are required. The sketch of configuration in the Chimera is displayed in Fig. 4(a).

We have set the annealing schedule to the default and also set the number of reads to 1,000 as before. The the distribution of energy and population of each configurations are shown in Fig. 4(b). The achieved minimum energy with this configuration is −1746.26 which is corresponding to the following vectors as the solution

25 $$\begin{array}{rcl}{v}_{0} & = & \,{(+,-,+,-,-,+,+,-,+,+,-,-)}^{T}\\ {v}_{1} & = & {(+,-,-,-,+,+,-,+,+,-,+,-)}^{T}\\ {v}_{2} & = & {(+,+,-,+,+,+,+,+,+,+,-,+)}^{T}\end{array}$$

We can verify that these set of three binary vectors are orthogonal to each others. The distribution of the solution shown in Fig. 4(b) is uniform, which means that every solution achieved minimum energy level. Among 1000 reads that we have set in the experiment, D-wave delivered 1000 different answers, where 635 are wrong and 365 are correct solutions. The connection diagram in Fig. 4(a) shows that for order-12, problem of finding three orthogonal binary vectors already occupied most of the qubits and connections of the processor.

#### Finding a missing vector of 12-order H-matrix

**Figure 5:**
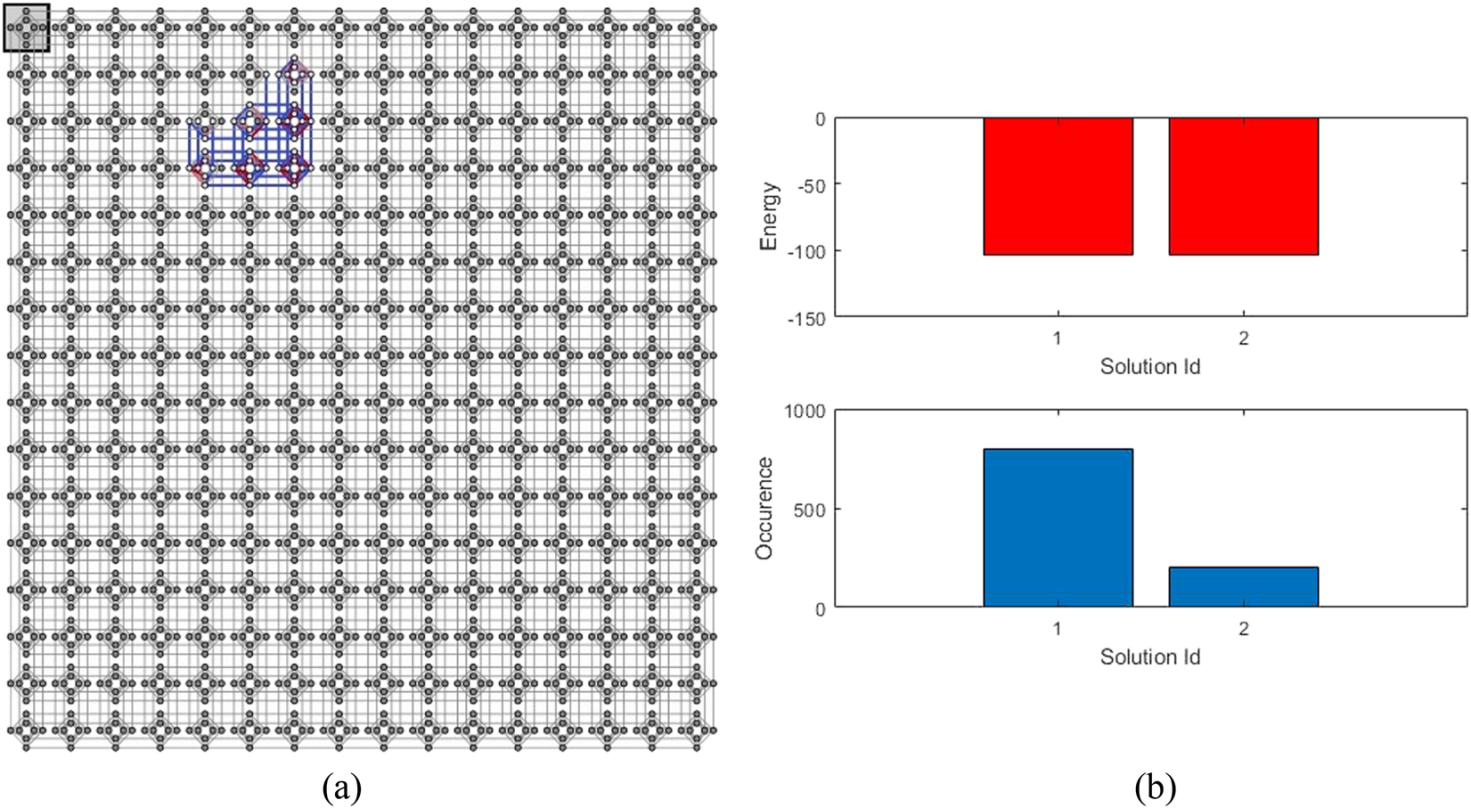
Implementation of finding 1-deleted vector of 12-order H-matrix’s Hamiltonian into a quantum annealer: (**a**) Embedding diagram of 28 logical qubits into 50 physical qubits in a Chimera-connected qubits of the DW2000Q, (**b**) Obtained results after quantum annealing: the distribution of energy related to each solution (top) and distribution of the solutions obtained by 1,000 reads (bottom).

In this experiment, the D-Wave is programmed to find one vector missing in an 12-order H-matrix. The known 11 vectors are identical to the simulation case given by Eq. (20). Based on the Hamiltonian given by Eq. (21), we realized that 28 logical qubits are needed. We rely on SAPI embedding module to configure the Chimera-connection of the qubits, which shows that 50 physical qubits are required. Figure 5(a) displays a realization of qubits connection in the Chimera graph. Although the order of the matrix is sufficiently high, since the required qubits and couplers for this problem are small, it only occupies a small area in the processor.

By using the default annealing schedule with 1000 reads as before, we have obtained the minimum energy of −104.00 and the following binary vector as a solution,

26 $${v}_{11}={(\begin{array}{c}-,+,-,-,-,+,+,+,-,+,+,-\end{array})}^{T}$$

which can be verified to be a correct one; i.e., along with 11 vectors in Eq. (20), this vector constructs a 12-order H-matrix. Figure 5(b) shows the distribution of energy and occurrence of the solutions. We see that only two kind of solutions are exists, both of them are at the identical minimum energy level.

## Discussions and Conclusions

We have investigated the possibility of quantum computing to solve the problem of finding H-matrix among possible binary matrices of the same order, which is a hard problem. The QAM or quantum annealer has been considered for its realization, which requires the problem to be translated into a Hamiltonian. We have proposed a method to formulate the Hamiltonian’s of finding H-matrix and its related problems.

Existing quantum annealer permits only up to quadratic terms for realization. Since the problem naturally induces higher order terms, we have to perform boolean reduction to obtain realizable Hamiltonians. Manipulation of large number of terms implied by both of growing number of variables with order and the boolean reduction procedure requires a computer-assisted process in constructing the Hamiltonians. The proposed method consists of a set of symbolic computing algorithms to formulate the energy function that lead to the Hamiltonian of the problems. The obtained Hamiltonians are then evaluated by both of simulation and implementation in a 2048 qubits DW2000Q quantum annealer.

For the H-SEARCH problem, an existing quantum annealer was able to find 4-order H-matrices. We also have successfully solved the problem of finding 3 orthogonal binary vectors of length 12 and the problem of finding 1 missing vector in a 12-order H-matrix. In the future, it is expected that higher order H-matrix searching problem can be solved when the device allows more than 2-body interaction or a better qubits connection beyond the Chimera graph is available.

## Supplementary information

Supplementary Information

## Data Availability

Most of the codes that have been used in this research are available in the following public accessed github: https://github.com/suksmono, https://github.com/mdrft. The data can be generated from the codes. All of related codes and data will be provided upon direct request to the authors.
