# Supplementary material for: Finding Hadamard Matrices by a Quantum Annealing Machine
Source: Sci Rep. 2019 Oct 7;9:14380. doi: 10.1038/s41598-019-50473-w (PMC6779766; doi:10.1038/s41598-019-50473-w)
Supplement: Supplementary file 1 — Supplementary Information [file 41598_2019_50473_MOESM1_ESM.pdf]

Supplementary Information
Finding Hadamard Matrices by a Quantum Annealing Machine

ANDRIYAN BAYU SUKSMONO, Institut Teknologi Bandung, Indonesia
YUICHIRO MINATO, MDR Inc., Japan

I. DETAIL DESCRIPTION OF THE METHODS

Finding a Hadamard Matrix By Energy Minimization

Consider an $M = 2,\ 4,\ 8,\ 12, \cdots, 4k$ order binary matrix B whose elements are $b_{i,j} \in \{-1, +1\}$, with k a positive integer (we have omitted $M = 1$ case due to its triviality). By writing the ith column vector of B as

$$\vec{b}_i = \left(b_{0,i}\ b_{1,i}\ \cdots\ b_{M-1,i}\right)^T \tag{S.1}$$

where $(\cdot)^T$ denotes matrix transpose operation, we can express the matrix $B = \left(\vec{b}_0\ \vec{b}_1 \cdots\ \vec{b}_{M-1}\right)$ as

$$B = \begin{pmatrix} b_{0,0} & b_{0,1} & \cdots & b_{0,M-1} \\ b_{1,0} & b_{1,1} & \cdots & b_{1,M-1} \\ \cdots & \cdots & \cdots & \cdots \\ b_{M-1,0} & b_{M-1,1} & \cdots & b_{M-1,M-1} \end{pmatrix}$$

To indicate the orthogonality relationship among the column vectors of B, we define a matrix $D \equiv B^T B$, which explicitly can be written as

$$D = \begin{pmatrix} \langle\vec{b}_0, \vec{b}_0\rangle & \langle\vec{b}_0, \vec{b}_1\rangle & \cdots & \langle\vec{b}_0, \vec{b}_{M-1}\rangle \\ \langle\vec{b}_1, \vec{b}_0\rangle & \langle\vec{b}_1, \vec{b}_1\rangle & \cdots & \langle\vec{b}_1, \vec{b}_{M-1}\rangle \\ \cdots & \cdots & \cdots & \\ \langle\vec{b}_{M-1}, \vec{b}_0\rangle & \langle\vec{b}_{M-1}, \vec{b}_1\rangle & \cdots & \langle\vec{b}_{M-1}, \vec{b}_{M-1}\rangle \end{pmatrix}$$

where $\langle\vec{b}_i, \vec{b}_j\rangle = \vec{b}_i^T \cdot \vec{b}_j$ is the inner product between (column) vector $\vec{b}_i$ and $\vec{b}_j$. By denoting $d_{ij} \equiv \langle\vec{b}_i, \vec{b}_j\rangle$ and knowing that $\langle\vec{b}_i, \vec{b}_i\rangle = M$, the indicator matrix D can be rewritten as

$$D = \begin{pmatrix} M & d_{0,1} & \cdots & d_{0,M-1} \\ d_{1,0} & M & \cdots & d_{1,M-1} \\ \cdots & \cdots & \cdots & \\ d_{M-1,0} & d_{M-1,1} & \cdots & M \end{pmatrix} \tag{S.2}$$

When all of $d_{i,j} = 0$ in Eq.(S.2), then, by definition, B is an orthogonal matrix; which due to its elements of being $\{-1, 1\}$, is also a H-matrix. Consequently, we can define the energy function as the sum of absolute values of the off-diagonal elements of D, which implies that a zero energy value corresponds to all of the column vectors being orthogonal to each other, whereas a non-zero value indicates that there is at least a pair of non-orthogonal vectors among them. Since D is a symmetric matrix, it is sufficient to consider only an upper- (or lower-) diagonal part of D, i.e., we can define the energy function for a given set of column vectors of $\left\{\vec{b}_i\right\}$ as

Authors' addresses: Andriyan Bayu Suksmono, suksmono@stei.itb.ac.id, Institut Teknologi Bandung, Jl. Ganesha No.10, Bandung, Indonesia; Yuichiro Minato, MDR Inc. Hongo 2-40-14-3F, Bunkyo-ku, Tokyo, Japan.

$$E_a\left(\left\{\vec{b}_i\right\}\right) \equiv \sum_{i<j}\left|\langle\vec{b}_i,\vec{b}_j\rangle\right| = \sum_{i<j}\left|d_{i,j}\right| \tag{S.3}$$

Furthermore, since we need to express the energy function as products of binary variables $b_{i,j}$'s, we have to change the absolute function into a square function. Then, Eq.(S.3) becomes

$$E_s\left(\left\{\vec{b}_i\right\}\right) = \sum_{i<j}\left(\langle\vec{b}_i,\vec{b}_j\rangle\right)^2$$

Considering Eq.(S.1), we can show that the square of the inner product between two binary vectors $d_{i,j}^2 = \langle\vec{b}_i,\vec{b}_j\rangle^2$ are given by

$$d_{i,j}^2 = \left(b_{0,i}b_{0,j} + b_{1,i}b_{1,j}\cdots + b_{M-1,i}b_{M-1,j}\right)^2$$

Expansion of the square terms yields the following expression

$$\begin{aligned} d_{i,j}^2 = b_{0,i}^2 b_{0,j}^2 + b_{1,i}^2 b_{1,j}^2 + \cdots + b_{M-1,i}^2 b_{M-1,j}^2 \\ +2b_{0,i}b_{0,j}b_{1,i}b_{1,j} + 2b_{0,i}b_{0,j}b_{2,i}b_{2,j} + \cdots + 2b_{0,i}b_{0,j}b_{M-1,i}b_{M-1,j} \\ +2b_{1,i}b_{1,j}b_{2,i}b_{2,j} + 2b_{1,i}b_{1,j}b_{3,i}b_{3,j} + \cdots + b_{1,i}b_{1,j}b_{M-1,i}b_{M-1,j} \\ +\cdots\cdots \\ +2b_{M-2,i}b_{M-2,j}b_{M-1,i}b_{M-1,j} \end{aligned}$$

Since $b_{i,j} \in \{-1,+1\}$, then $b_{i,j}^2 = 1$. Therefore, we can simplify $d_{i,j}^2$ into

$$d_{i,j}^2 = M + 2\sum_{m<n<M} b_{m,i}b_{m,j}b_{n,i}b_{n,j} \tag{S.4}$$

Finally, the energy function related to orthogonality condition of all pairs of the column vectors in B can be expressed as

$$E_s\left(\{\vec{b}_i\}\right) = \sum_{i<j} d_{i,j}^2 = \frac{M^2(M-1)}{2} + 2\sum_{m<n<M,\, i<j<M} b_{m,i}b_{m,j}b_{n,i}b_{n,j} \tag{S.5}$$

In our previous papers [1, 2], we have employed energy function that is similar to Eq.(S.3). For implementation in a QAM, we need a modified form of Eq.(S.5). First, we introduce a spin variable $s = \{-1,+1\}$ and a Boolean variable $q = \{0,1\}$, whose location at site i will be denoted by s_i and q_i, respectively. Both of these variables are related by transforms given by Eq.(4) and Eq.(5) (in the main text).

Since the elements of a H-matrix are $\{-1,1\}$, it is natural to formulate the energy function of H-SEARCH in the s-domain. Therefore, first we will express the energy function in this domain. We also reassign the index of the variables from the row-column format to a single contiguous indices ranging from 0 to $(M-1)^2$, i.e., we prefer to use a single indexed variable s_i rather than the previously double indexed $b_{i,j}$. The notation of its related energy function is changed by

$$E_s\left(\{\vec{b}_i\}\right) \rightarrow E_k\left(\{s_i\}\right) \equiv E_k(s) \tag{S.6}$$

Accordingly, Eq.(S.5) is changed into Eq.(7).

Considering the implementation in a QAM, we further need to transform the k-body energy function of Eq.(7) to a 2-body energy function, which normally is formulated in the q-domain. Following the formulation described in [3, 4], a k-body interaction can be converted into a 2-body interaction by substitution and an additional compensation given by Eq.(8) and Eq.(9), respectively.

According to [4], the value of $\delta_{i,j}$ should be chosen to be larger than the maximum value of its substituted function of energy, which in our case is d_{ij}^2. The substitution variable is also called an ancillary variable or simply called *ancilla*, whereas the original one will be refered to as *main variable*.

The input of a QAM or its simulator needs parameters (Ising coefficients) in s-domain. Therefore, from a general k-body interaction in s-domain energy function $E_k(s)$, we will transform it into $E_2(s)$ and eventually to its Hamiltonian $\hat{H}_2(\hat{\sigma})$ by using steps given by the Hamiltonian's *construction diagram* given by Eq.(10). In the following discussions, we will describe each of these transforms in the diagram and present examples to clarify the construction process.

First note that according to the transform given by Eq.(4), the q-transformed energy from Eq.(7) into $E_k(q)$ will contain quartic terms $q_i q_j q_m q_n$. We observed that each term in $q_i q_j$ actually comes from a product of two column vectors $\sum_{r<M} q_{r,i} q_{r,j}$ (and so is $q_m q_n$). Therefore, it will be more convenient to arrange the substitution of $q_{r,i} q_{r,j} \leftarrow q_{r,t}$ (and so is $q_m q_n$) column-wise. Then, we can make the arrangement of variables of the H-matrix and related ancillas as shown by the following table

main variables					ancillas			
q_0	q_M	q_{2M}	$\cdots$	$q_{(M-1)M}$	q_{M^2}	q_{M^2+M}	$\cdots$	$q_{M^2+M^2(M-1)/2-M+1}$
q_1	q_{M+1}	q_{2M+1}	$\cdots$	$q_{(M-1)M+1}$	q_{M^2+1}	q_{M^2+M+1}	$\cdots$	$q_{M^2+M^2(M-1)/2-M+2}$
$\cdots$	$\cdots$	$\cdots$	$\cdots$	$\cdots$	$\cdots$	$\cdots$	$\cdots$	$\cdots$
q_{M-1}	q_{2M-1}	q_{3M-1}	$\cdots$	q_{M^2-1}	q_{M^2+M-1}	q_{M^2+2M-1}	$\cdots$	$q_{M^2+M^2(M-1)/2}$

Left part of the table shows (main) variables of the matrix elements, whereas the right parts are ancillas. Using this arrangement, the substitution of a product of two binary variables by a single binary variable is done as follows

$$\begin{array}{ccc} q_0 q_M \leftarrow q_{M^2} & q_0 q_{2M} \leftarrow q_{M^2+M} & \cdots \\ q_1 q_{M+1} \leftarrow q_{M^2+1} & q_1 q_{2M} \leftarrow q_{M^2+M+1} & \cdots \\ \cdots & \cdots & \cdots \\ q_{M-1} q_{2M-1} \leftarrow q_{M^2+M+1} & q_{M-1} q_{3M-1} \leftarrow q_{M^2+2M-1} & \cdots \end{array} \tag{S.7}$$

We can adopt similar conventions for the s-domain. The arrangement of variables is then given by the following table

main variables					ancillas			
s_0	s_M	s_{2M}	$\cdots$	$s_{(M-1)M}$	s_{M^2}	s_{M^2+M}	$\cdots$	$s_{M^2+M^2(M-1)/2-M+1}$
s_1	s_{M+1}	s_{2M+1}	$\cdots$	$s_{(M-1)M+1}$	s_{M^2+1}	s_{M^2+M+1}	$\cdots$	$s_{M^2+M^2(M-1)/2-M+2}$
$\cdots$	$\cdots$	$\cdots$	$\cdots$	$\cdots$	$\cdots$	$\cdots$	$\cdots$	$\cdots$
s_{M-1}	s_{2M-1}	s_{3M-1}	$\cdots$	s_{M^2-1}	s_{M^2+M-1}	s_{M^2+2M-1}	$\cdots$	$s_{M^2+M^2(M-1)/2}$

whereas the substitution scheme of a product of two-binary variables by a single variable will be conducted as follows

$$\begin{array}{ccc} s_0 s_M \leftarrow s_{M^2} & s_0 s_{2M} \leftarrow s_{M^2+M} & \cdots \\ s_1 s_{M+1} \leftarrow s_{M^2+1} & s_1 s_{2M} \leftarrow s_{M^2+M+1} & \cdots \\ \cdots & \cdots & \cdots \\ s_{M-1} s_{2M-1} \leftarrow s_{M^2+M+1} & s_{M-1} s_{3M-1} \leftarrow s_{M^2+2M-1} & \cdots \end{array} \tag{S.8}$$

In practice, we do not perform the transform given by Eq.(S.8) directly since the substitution of a k-body to a 2-body interaction is always performed in the q-domain. The substitution in s-domain follows automatically when we transform the domain from $E_2(q)$ into $E_2(s)$ by substitution of variable $q_i \leftarrow s_i$.

Hamiltonian Formulation: Illustration by Low Order Case

To clarify the method, we will explain the Hamiltonian formulation step-by-step for a low order case, which in this case is a H-matrix of order 2. The discussions follow the stages as illustrated by the Hamiltonian construction diagram depicted in Eq. (10).

Formulation of $E_k(s)$. The formulation of energy function is started by an arrangement of variables, which for the finding H-matrix of order 2 problem is given by the followings

$$\begin{pmatrix} s_0 & s_2 \\ s_1 & s_3 \end{pmatrix}$$

The energy function is defined as the total sum of square of the off-diagonal elements of D-matrix, which in this case will only consist of a single term $d_{0,1}$. By using Eq.(7) we obtain $E_k(s) = d_{0,1}^2 = (s_0 s_2 + s_1 s_3)^2$ which leads to the following

$$E_k(s) = \left(s_0^2 s_2^2 + s_1^2 s_3^2\right) + 2\left(s_0 s_2 s_1 s_3\right) \tag{S.9}$$

By substitution of $s_i^2 \leftarrow 1$ into $E_k(s)$, we arrive to the following form

$$E_k(s) = 2 + 2 s_0 s_1 s_2 s_3 \tag{S.10}$$

The substitution $s_i^2 \leftarrow 1$ which is done in the last step simplifies greatly Eq.(S.9) into Eq.(S.10); this is one of the important steps to be highlighted in formulating the energy function.

Transformation $E_k(s) \longrightarrow E_k(q)$. To obtain $E_k(q)$, we perform $q_i \leftarrow s_i$ substitution which is defined by Eq.(4). Even for a two-term case of Eq.(S.10), the number of terms starts to increase significantly into 16, which is given by the following expression

$$\begin{aligned} E_k(q) = 4 - 4q_0 - 4q_1 - 4q_2 - 4q_3 + 8q_0q_1 + 8q_0q_2 + 8q_0q_3 + 8q_1q_2 + 8q_1q_3 + 8q_2q_3 \\ -16q_0q_1q_2 - 16q_0q_1q_3 - 16q_0q_2q_3 - 16q_1q_2q_3 + 32q_0q_1q_2q_3 \end{aligned} \tag{S.11}$$

By observing the terms in Eq.(S.11), we realize that the q-domain energy function $E_k(q)$ contains constants, quadratics, cubics, and a quartic terms. The cubics and quartics terms should be converted into at most quadratics terms for implementation into a QAM.

Transformation $E_k(q) \longrightarrow E_2(q)$. To reduce the degree of high order terms (cubics and quartics) into at most second order (quadratics), we employ the substitution by considering the following arrangement of variables as explained in the previous section

main variables		ancillas
q_0	q_2	q_4
q_1	q_3	q_5

Based on the arrangement, the substitutions to be done are $q_0 q_2 \leftarrow q_4$ and $q_1 q_3 \leftarrow q_5$, each of which is compensated by its corresponding $C_\wedge$. Then, based on Eq.(8) and Eq.(9), we should proceed as follows

$$\begin{aligned} q_0 q_2 &\leftarrow q_4 + \delta_{0,2}\left(3q_4 + q_0q_2 - 2q_0q_4 - 2q_2q_4\right) \\ q_1 q_3 &\leftarrow q_5 + \delta_{1,3}\left(3q_5 + q_1q_3 - 2q_1q_5 - 2q_3q_5\right) \end{aligned}$$

Since the substitution is done independently for each of the terms in $d_{i,j}^2$, the value of $\delta_{i,j}$ is determined by maximum value of $d_{i,j}^2 = M^2$. In our case, we take $\delta_{0,2} = \delta_{1,3} \equiv \delta = 4M^2 = 16$ for all

terms undergoing the substitution. The result for finding 2-order H-matrix problem is a 22-terms q-domain energy function given as follows

$$\begin{aligned} E_2(q) = 4 - 4q_0 - 4q_1 - 4q_2 - 4q_3 + 56q_4 + 56q_5 + 8q_0q_1 + 16q_0q_2 + 8q_0q_3 - 32q_0q_4 - 16q_0q_5 \\ +8q_1q_2 + 16q_1q_3 - 16q_1q_4 - 32q_1q_5 + 8q_2q_3 - 32q_2q_4 - 16q_2q_5 - 16q_3q_4 - 32q_3q_5 + 32q_4q_5 \end{aligned} \tag{S.12}$$

Transformation $E_2(q) \longrightarrow E_2(s)$. After obtaining the $E_2(q)$ expression, based on the construction diagram, now we should transform it back to s-domain to obtain $E_2(s)$. The result is an s-domain energy function that also consists of 22 terms given as follows

$$\begin{aligned} E_2(s) = 28 + 6s_0 + 6s_1 + 6s_2 + 6s_3 - 12s_4 - 12s_5 + 2s_0s_1 + 4s_0s_2 + 2s_0s_3 - 8s_0s_4 - 4s_0s_5 \\ +2s_1s_2 + 4s_1s_3 - 4s_1s_4 - 8s_1s_5 + 2s_2s_3 - 8s_2s_4 - 4s_2s_5 - 4s_3s_4 - 8s_3s_5 + 8s_4s_5 \end{aligned} \tag{S.13}$$

Formulation of 2-Body Hamiltonian: $E_2(s) \longrightarrow \hat{H}_2(\hat{\sigma})$. The formulation of Hamiltonian for a given $E_2(s)$ is done by substitution of $s_i \longleftarrow \hat{\sigma}_i^z$. Based on Eq.(S.13), we arrive to the Hamiltonian of a 2-body interaction for H-SEARCH problem of order 2 given by Eq.(11). At this point, we can see that for implementation on a QAM, a simple two-terms s-domain energy function with only one quartic terms given by Eq.(7) transform into a 22-terms Hamiltonian given by Eq.(11). Computation by hand for a higher order H-matrix problem surely will be not an easy task. This issue will be addressed in the following section.

Higher Order Case: The Needs of Symbolic Computing

The method to formulate Hamiltonian of finding 2-order H-matrix that has been described previously can be generalized to higher orders. It will be realized immediately that the problem start to occur due to the increasing number of variables and terms. An M order H-SEARCH needs M^2 number of binary variables to represent the matrix and an additional of $M \times M(M-1)/2$ for the ancillas, giving $M^2 + M \times M(M-1)/2$ in total. Therefore, we have increased the number of variables (complexity) from $O(M^2)$ to $O(M^3)$. In the following discussion, when an expression of energy function or a Hamiltonian includes too many terms to write, we will only display partially. The complete expressions are provided separately in Appendix section.

As an example, a problem of finding 4-order H-matrix needs 40 binary variables, which consists of 16 main variables and 24 ancillas. We can arrange the s-variables as follows

main variables				ancillas					
s_0	s_4	s_8	s_{12}	s_{16}	s_{20}	s_{24}	s_{28}	s_{32}	s_{36}
s_1	s_5	s_9	s_{13}	s_{17}	s_{21}	s_{25}	s_{29}	s_{33}	s_{37}
s_2	s_6	s_{10}	s_{14}	s_{18}	s_{22}	s_{26}	s_{30}	s_{34}	s_{38}
s_3	s_7	s_{11}	s_{15}	s_{19}	s_{23}	s_{27}	s_{31}	s_{35}	s_{39}

Similarly, this problem also needs 40 number of q-variables arranged as follows,

main variables				ancillas					
q_0	q_4	q_8	q_{12}	q_{16}	q_{20}	q_{24}	q_{28}	q_{32}	q_{36}
q_1	q_5	q_9	q_{13}	q_{17}	q_{21}	q_{25}	q_{29}	q_{33}	q_{37}
q_2	q_6	q_{10}	q_{14}	q_{18}	q_{22}	q_{26}	q_{30}	q_{34}	q_{38}
q_3	q_7	q_{11}	q_{15}	q_{19}	q_{23}	q_{27}	q_{31}	q_{35}	q_{39}

Although the formulation of $E_k(s)$ can be done similarly to the 2-order case, due to a large number of variables and terms, it will be better to automatize this process in a computer, i.e., we employ

symbolic computing software to derive the energy function. We have formulated **Algorithm S.1** to calculate $E_k(s)$.

Algorithm S.1 Construction of $E_k(s)$ by symbolic computation

1: Construct array of variables $\{s_i, s_j\}$ according to the order M of the H-matrix
2: Calculate inner product $d_{ij} = \langle s_i, s_j \rangle$ of symbols (variables) between every pairs of columns of the array
3: Calculate $E_k(s) = \sum d_{i,j}^2$
4: Cleanup s_i^2 terms by substitution: $E_k(s) = E_k(s)|_{s_i^2 \leftarrow 1}$

The **Algorithm S.1** can be implemented into a programming language that has a symbolic computing capability. The energy function of finding 4-order H-matrix problem is given as follows,

$$E_k(s) = \sum_{i<j<M} d_{ij}^2$$

Expanding the this energy function will generate a 37-terms expression that can be written as follows,

$$E_k(s) = 24 + 2s_0 s_1 s_4 s_5 + \cdots + 2s_0 s_1 s_{12} s_{13} + \cdots + 2s_{10} s_{11} s_{14} s_{15} \tag{S.14}$$

Likewise, the transformation of $E_k(s) \rightarrow E_k(q)$ can also be done automatically by using **Algorithm S.2**.

Algorithm S.2 Transform $E_k(s) \rightarrow E_k(q)$

1: For all binary variables s_i:
2: $\quad E_k(s)|_{s_i \leftarrow (1-2q_i)}$

The processing of the 4-order case yields an energy function with 317 terms, which by setting $\delta = 4 \times H_{max} = 64$, can be expressed as follows

$$E_k(q) = 96 - 36q_0 + \cdots - 36q_{15} + \cdots + 24q_{14}q_{15} + \cdots - 16q_{11}q_{14}q_{15} + \cdots + 32q_{10}q_{11}q_{14}q_{15} \tag{S.15}$$

The next stage of transforming $E_k(q) \rightarrow E_2(q)$ will yield an energy function with more number of terms. The processing for such transform is described in **Algorithm S.3**.

Algorithm S.3 Transform $E_k(q) \rightarrow E_2(q)$

1: Construct a list of substitution pair $sPair[col_i, col_j, col_k]$
2: $E_2(q) \leftarrow E_k(q)$
3: For all high order terms in $E_2(q)$ and based on $sPair$:
4: $\quad E_2(q) \leftarrow E_2(q)|_{q_i \cdot q_j \leftarrow q_k} + H_\wedge(q_i, q_j, q_k, \delta)$
5: Simplify $E_2(q)$

In the finding 4-order H-matrix case, the two-body q-domain energy function $E_2(q)$ will consist of 389 terms, which are given as the followings,

$$E_2(q) = 96 - 36q_0 + \cdots + 216q_{39} + 24q_0q_1 + \cdots + 32q_{38}q_{39} \tag{S.16}$$

The last stage of transformation $E_2(q) \rightarrow E_2(s)$ can be done by $q_i \leftarrow s_i$ substitution based on the **Algorithm S.4** .

Algorithm S.4 Transform $E_2(q) \rightarrow E_2(s)$

1: For all variables $\{q_i\}$:
2: $\quad E_2(s) \leftarrow E_2(q)\big|_{q_i \leftarrow \left(\frac{1-s_i}{2}\right)}$

Finally, by combining all of the these algorithms, we obtain will Algorithm 1 of the main text.

The final form of energy function $E_2(s)$ also consists of 389 terms can be expressed as the followings,

$$E_2(s) = 1,248 + 66s_0 + \cdots - 44s_{39} + 6s_0s_1 + \cdots + 8s_{38}s_{39} \tag{S.17}$$

The Hamiltonian of the problem can be obtained directly from Eq.(S.17) by replacing the binary variables by the corresponding operators $s_i \leftarrow \hat{\sigma}_i^z$. The result is given by Eq.(12), which is the desired Hamiltonian of the 4-order H-SEARCH problem, which will become $\hat{H}_{pot}(\hat{\sigma})$ in the quantum annealing process given by Eq.(3).

Sub-Problem-1 (Problem-1 in the main text):Finding a Set of $N < M$ Orthogonal Binary Vectors

In this sub-problem, we want to find N number of M-length binary vectors, where $N < M$. The initial values of the binary variables of the N-vectors, which generally non-orthogonal to each other, can be set to either particular values or at random; therefore, this process of obtaining N-orthogonal vectors from the given initial vectors will also be called *orthogonalization.* We arrange the variables similarly as before, but now with less number of variables. The number of ancillas is also reduced to $N \times N \times (N-1)/2$. We start with the following arrangement of s-variables

main variables					ancillas			
s_0	s_M	s_{2M}	$\cdots$	$s_{(N-1)M}$	s_{NM}	s_{M^2+M}	$\cdots$	$s_{NM+NM(M-1)/2-M+1}$
s_1	s_{M+1}	s_{2M+1}	$\cdots$	$s_{(N-1)M+1}$	s_{NM+1}	s_{M^2+M+1}	$\cdots$	$s_{NM+MN(N-1)/2-M+2}$
$\cdots$	$\cdots$	$\cdots$	$\cdots$	$\cdots$	$\cdots$	$\cdots$	$\cdots$	$\cdots$
s_{M-1}	s_{2M-1}	s_{3M-1}	$\cdots$	$s_{(N-1)(M-1)}$	s_{NM+M-1}	s_{M^2+2M-1}	$\cdots$	$s_{NM+MN(N-1)/2}$

For a concrete illustration, consider $M = 4$ and $N = 3$, i.e., finding a set of 3 binary ortho-vectors of order 4. The arrangement of variables becomes as follows

main variables			ancillas		
s_0	s_4	s_8	s_{12}	s_{16}	s_{20}
s_1	s_5	s_9	s_{13}	s_{17}	s_{21}
s_2	s_6	s_{10}	s_{14}	s_{18}	s_{22}
s_3	s_7	s_{11}	s_{15}	s_{19}	s_{23}

Compared to finding 4-order H-matrix problem, after performing the process described by the construction diagram, we found that the number of terms in $E_k(s)$ has been reduced to 19, whereas there are 169 number of terms in $E_k(q)$, and 205 terms in each of $E_2(q)$ and $E_2(s)$. The Hamiltonian of the system with 205 terms is given by Eq.(13).

Sub-Problem-2 (Problem-3 in the main text): Hadamard-Matrix Completion

In the H-matrix completion problem, the task is to find N-number of missing vectors of an M-order H-matrix. This means that $(M - N)$ number of (column) vectors are known. Construction of a

H-matrix by random generation of M-order binary vector followed by orthogonality testing implies that finding the last vectors of a H-matrix become increasingly difficult. It can be understood considering the orthogonality of a candidate vector should be tested to previously found vectors. Interestingly, in this sub-problem, we can use the known vectors as a constraint which further reduce the number of variables and therefore the number of qubits needed in the implementation of the problem in a QAM.

Consider the problem of finding 1 missing vector in a 2-order H-matrix. When it is a seminormalized one, all elements in the first columns are 1's. Then, we have the following form of variable arrangements

main variables		ancillas
1	s_0	*
1	s_1	*

Note that in this case we do not need any ancilla, so that we put "*" to all of ancilla's positions in the table. The expression of $E_k(s)$, after substitution $s_0^2 \leftarrow 1$, becomes

$$E_k(s) = (s_0 + s_1)^2|_{s_i^2 \leftarrow 1} = 2 + 2s_0s_1 = 2(1 + s_0s_1)$$

It is easy to see that the minimum value of this energy function, which is 0, will be achieved when $s_0s_1 = -1$, i.e., either $s_0 = -1$ and $s_1 = 1$ or $s_0 = 1$ and $s_1 = -1$, which then gives the following solutions of the H-matrices

$$\begin{pmatrix} + & - \\ + & + \end{pmatrix} \; and \; \begin{pmatrix} + & + \\ + & - \end{pmatrix}$$

Note that for conciseness, we have represented the elements by their signs, i.e, -1 is displayed as $-$, whereas 1 is shown as $+$.

Higher order cases can be treated similarly. Consider the problem of finding 2 missing vectors in a 4-order H-matrix. Instead of writing the known vectors at the first columns, we have written them in the last ones for convenience of indexing the variables and ancillas. The arrangement will become as follows,

main variables				ancillas
s_0	s_4	+	+	s_8
s_1	s_5	-	+	s_9
s_2	s_6	+	+	s_{10}
s_3	s_7	-	+	s_{11}

By following the previously explained symbolic computational procedures, we will obtain 11 terms in $E_k(s)$, 67 terms in $E_k(q)$, and 79 terms in each of $E_2(q)$ and $E_2(s)$. The Hamiltonian of the system, which also consists of 79 terms is given by Eq.(14). Higher order case is discussed and tested in the experiment section of the main text. Considering the current number of available qubits and connections in DW2000Q, we will try to find 1-deleted vector of a 12-order H-matrix.

II. COMPLETE EXPRESSION OF ENERGY FUNCTIONS AND HAMILTONIANS

A complete expression of Eq.(S.14)

$$E_k(s) = 2s_0s_1s_{12}s_{13} + 2s_0s_1s_4s_5 + 2s_0s_1s_8s_9 + 2s_0s_{10}s_2s_8 + 2s_0s_{11}s_3s_8 + 2s_0s_{12}s_{14}s_2 + 2s_0s_{12}s_{15}s_3 + 2s_0s_2s_4s_6 + 2s_0s_3s_4s_7 + 2s_1s_{10}s_2s_9 + 2s_1s_{11}s_3s_9 + 2s_1s_{13}s_{14}s_2 + 2s_1s_{13}s_{15}s_3 + 2s_1s_2s_5s_6 + 2s_1s_3s_5s_7 + 2s_{10}s_{11}s_{14}s_{15} + 2s_{10}s_{11}s_2s_3 + 2s_{10}s_{11}s_6s_7 + 2s_{10}s_{12}s_{14}s_8 + 2s_{10}s_{13}s_{14}s_9 + 2s_{10}s_4s_6s_8 + 2s_{10}s_5s_6s_9 + 2s_{11}s_{12}s_{15}s_8 + 2s_{11}s_{13}s_{15}s_9 + 2s_{11}s_4s_7s_8 + 2s_{11}s_5s_7s_9 + 2s_{12}s_{13}s_4s_5 + 2s_{12}s_{13}s_8s_9 + 2s_{12}s_{14}s_4s_6 + 2s_{12}s_{15}s_4s_7 + 2s_{13}s_{14}s_5s_6 + 2s_{13}s_{15}s_5s_7 + 2s_{14}s_{15}s_2s_3 + 2s_{14}s_{15}s_6s_7 + 2s_2s_3s_6s_7 + 2s_4s_5s_8s_9 + 24$$

A complete expression of Eq.(S.15)

$$E_k(q) = 32q_0q_1q_{12}q_{13} - 16q_0q_1q_{12} - 16q_0q_1q_{13} + 32q_0q_1q_4q_5 - 16q_0q_1q_4 - 16q_0q_1q_5 + 32q_0q_1q_8q_9 - 16q_0q_1q_8 - 16q_0q_1q_9 + 24q_0q_1 + 32q_0q_{10}q_2q_8 - 16q_0q_{10}q_2 - 16q_0q_{10}q_8 + 8q_0q_{10} + 32q_0q_{11}q_3q_8 - 16q_0q_{11}q_3 - 16q_0q_{11}q_8 + 8q_0q_{11} - 16q_0q_{12}q_{13} + 32q_0q_{12}q_{14}q_2 - 16q_0q_{12}q_{14} + 32q_0q_{12}q_{15}q_3 - 16q_0q_{12}q_{15} - 16q_0q_{12}q_2 - 16q_0q_{12}q_3 + 24q_0q_{12} + 8q_0q_{13} - 16q_0q_{14}q_2 + 8q_0q_{14} - 16q_0q_{15}q_3 + 8q_0q_{15} + 32q_0q_2q_4q_6 - 16q_0q_2q_4 - 16q_0q_2q_6 - 16q_0q_2q_8 + 24q_0q_2 + 32q_0q_3q_4q_7 - 16q_0q_3q_4 - 16q_0q_3q_7 - 16q_0q_3q_8 + 24q_0q_3 - 16q_0q_4q_5 - 16q_0q_4q_6 - 16q_0q_4q_7 + 24q_0q_4 + 8q_0q_5 + 8q_0q_6 + 8q_0q_7 - 16q_0q_8q_9 + 24q_0q_8 + 8q_0q_9 - 36q_0 + 32q_1q_{10}q_2q_9 - 16q_1q_{10}q_2 - 16q_1q_{10}q_9 + 8q_1q_{10} + 32q_1q_{11}q_3q_9 - 16q_1q_{11}q_3 - 16q_1q_{11}q_9 + 8q_1q_{11} - 16q_1q_{12}q_{13} + 8q_1q_{12} + 32q_1q_{13}q_{14}q_2 - 16q_1q_{13}q_{14} + 32q_1q_{13}q_{15}q_3 - 16q_1q_{13}q_{15} - 16q_1q_{13}q_2 - 16q_1q_{13}q_3 + 24q_1q_{13} - 16q_1q_{14}q_2 + 8q_1q_{14} - 16q_1q_{15}q_3 + 8q_1q_{15} + 32q_1q_2q_5q_6 - 16q_1q_2q_5 - 16q_1q_2q_6 - 16q_1q_2q_9 + 24q_1q_2 + 32q_1q_3q_5q_7 - 16q_1q_3q_5 - 16q_1q_3q_7 - 16q_1q_3q_9 + 24q_1q_3 - 16q_1q_4q_5 + 8q_1q_4 - 16q_1q_5q_6 - 16q_1q_5q_7 + 24q_1q_5 + 8q_1q_6 + 8q_1q_7 - 16q_1q_8q_9 + 8q_1q_8 + 24q_1q_9 - 36q_1 + 32q_{10}q_{11}q_{14}q_{15} - 16q_{10}q_{11}q_{14} - 16q_{10}q_{11}q_{15} + 32q_{10}q_{11}q_2q_3 - 16q_{10}q_{11}q_2 - 16q_{10}q_{11}q_3 + 32q_{10}q_{11}q_6q_7 - 16q_{10}q_{11}q_6 - 16q_{10}q_{11}q_7 + 24q_{10}q_{11} + 32q_{10}q_{12}q_{14}q_8 - 16q_{10}q_{12}q_{14} - 16q_{10}q_{12}q_8 + 8q_{10}q_{12} + 32q_{10}q_{13}q_{14}q_9 - 16q_{10}q_{13}q_{14} - 16q_{10}q_{13}q_9 + 8q_{10}q_{13} - 16q_{10}q_{14}q_{15} - 16q_{10}q_{14}q_8 - 16q_{10}q_{14}q_9 + 24q_{10}q_{14} + 8q_{10}q_{15} - 16q_{10}q_2q_3 - 16q_{10}q_2q_8 - 16q_{10}q_2q_9 + 24q_{10}q_2 + 8q_{10}q_3 + 32q_{10}q_4q_6q_8 - 16q_{10}q_4q_6 - 16q_{10}q_4q_8 + 8q_{10}q_4 + 32q_{10}q_5q_6q_9 - 16q_{10}q_5q_6 - 16q_{10}q_5q_9 + 8q_{10}q_5 - 16q_{10}q_6q_7 - 16q_{10}q_6q_8 - 16q_{10}q_6q_9 + 24q_{10}q_6 + 8q_{10}q_7 + 24q_{10}q_8 + 24q_{10}q_9 - 36q_{10} + 32q_{11}q_{12}q_{15}q_8 - 16q_{11}q_{12}q_{15} - 16q_{11}q_{12}q_8 + 8q_{11}q_{12} + 32q_{11}q_{13}q_{15}q_9 - 16q_{11}q_{13}q_{15} - 16q_{11}q_{13}q_9 + 8q_{11}q_{13} - 16q_{11}q_{14}q_{15} + 8q_{11}q_{14} - 16q_{11}q_{15}q_8 - 16q_{11}q_{15}q_9 + 24q_{11}q_{15} - 16q_{11}q_2q_3 + 8q_{11}q_2 - 16q_{11}q_3q_8 - 16q_{11}q_3q_9 + 24q_{11}q_3 + 32q_{11}q_4q_7q_8 - 16q_{11}q_4q_7 - 16q_{11}q_4q_8 + 8q_{11}q_4 + 32q_{11}q_5q_7q_9 - 16q_{11}q_5q_7 - 16q_{11}q_5q_9 + 8q_{11}q_5 - 16q_{11}q_6q_7 + 8q_{11}q_6 - 16q_{11}q_7q_8 - 16q_{11}q_7q_9 + 24q_{11}q_7 + 24q_{11}q_8 + 24q_{11}q_9 - 36q_{11} + 32q_{12}q_{13}q_4q_5 - 16q_{12}q_{13}q_4 - 16q_{12}q_{13}q_5 + 32q_{12}q_{13}q_8q_9 - 16q_{12}q_{13}q_8 - 16q_{12}q_{13}q_9 + 24q_{12}q_{13} - 16q_{12}q_{14}q_2 + 32q_{12}q_{14}q_4q_6 - 16q_{12}q_{14}q_4 - 16q_{12}q_{14}q_6 - 16q_{12}q_{14}q_8 + 24q_{12}q_{14} - 16q_{12}q_{15}q_3 + 32q_{12}q_{15}q_4q_7 - 16q_{12}q_{15}q_4 - 16q_{12}q_{15}q_7 - 16q_{12}q_{15}q_8 + 24q_{12}q_{15} + 8q_{12}q_2 + 8q_{12}q_3 - 16q_{12}q_4q_5 - 16q_{12}q_4q_6 - 16q_{12}q_4q_7 + 24q_{12}q_4 + 8q_{12}q_5 + 8q_{12}q_6 + 8q_{12}q_7 - 16q_{12}q_8q_9 + 24q_{12}q_8 + 8q_{12}q_9 - 36q_{12} - 16q_{13}q_{14}q_2 + 32q_{13}q_{14}q_5q_6 - 16q_{13}q_{14}q_5 - 16q_{13}q_{14}q_6 - 16q_{13}q_{14}q_9 + 24q_{13}q_{14} - 16q_{13}q_{15}q_3 + 32q_{13}q_{15}q_5q_7 - 16q_{13}q_{15}q_5 - 16q_{13}q_{15}q_7 - 16q_{13}q_{15}q_9 + 24q_{13}q_{15} + 8q_{13}q_2 + 8q_{13}q_3 - 16q_{13}q_4q_5 + 8q_{13}q_4 - 16q_{13}q_5q_6 - 16q_{13}q_5q_7 + 24q_{13}q_5 + 8q_{13}q_6 + 8q_{13}q_7 - 16q_{13}q_8q_9 + 8q_{13}q_8 + 24q_{13}q_9 - 36q_{13} + 32q_{14}q_{15}q_2q_3 - 16q_{14}q_{15}q_2 - 16q_{14}q_{15}q_3 + 32q_{14}q_{15}q_6q_7 - 16q_{14}q_{15}q_6 - 16q_{14}q_{15}q_7 + 24q_{14}q_{15} - 16q_{14}q_2q_3 + 24q_{14}q_2 + 8q_{14}q_3 - 16q_{14}q_4q_6 + 8q_{14}q_4 - 16q_{14}q_5q_6 + 8q_{14}q_5 - 16q_{14}q_6q_7 + 24q_{14}q_6 + 8q_{14}q_7 + 8q_{14}q_8 + 8q_{14}q_9 - 36q_{14} - 16q_{15}q_2q_3 + 8q_{15}q_2 + 24q_{15}q_3 - 16q_{15}q_4q_7 + 8q_{15}q_4 - 16q_{15}q_5q_7 + 8q_{15}q_5 - 16q_{15}q_6q_7 + 8q_{15}q_6 + 24q_{15}q_7 + 8q_{15}q_8 + 8q_{15}q_9 - 36q_{15} + 32q_2q_3q_6q_7 - 16q_2q_3q_6 - 16q_2q_3q_7 + 24q_2q_3 - 16q_2q_4q_6 + 8q_2q_4 - 16q_2q_5q_6 + 8q_2q_5 - 16q_2q_6q_7 + 24q_2q_6 + 8q_2q_7 + 8q_2q_8 + 8q_2q_9 - 36q_2 - 16q_3q_4q_7 + 8q_3q_4 - 16q_3q_5q_7 + 8q_3q_5 - 16q_3q_6q_7 + 8q_3q_6 + 24q_3q_7 + 8q_3q_8 + 8q_3q_9 - 36q_3 + 32q_4q_5q_8q_9 - 16q_4q_5q_8 - 16q_4q_5q_9 + 24q_4q_5 - 16q_4q_6q_8 + 24q_4q_6 - 16q_4q_7q_8 + 24q_4q_7 - 16q_4q_8q_9 + 24q_4q_8 + 8q_4q_9 - 36q_4 - 16q_5q_6q_9 + 24q_5q_6 - 16q_5q_7q_9 + 24q_5q_7 - 16q_5q_8q_9 + 8q_5q_8 + 24q_5q_9 - 36q_5 + 24q_6q_7 + 8q_6q_8 + 8q_6q_9 - 36q_6 + 8q_7q_8 + 8q_7q_9 - 36q_7 + 24q_8q_9 - 36q_8 - 36q_9 + 96$$

A complete expression of Eq.(S.16)

$$E_2(q) = 24q_0q_1 + 8q_0q_{10} + 8q_0q_{11} + 64q_0q_{12} + 8q_0q_{13} + 8q_0q_{14} + 8q_0q_{15} - 128q_0q_{16} - 16q_0q_{17} - 16q_0q_{18} - 16q_0q_{19} + 24q_0q_2 - 128q_0q_{20} - 16q_0q_{21} - 16q_0q_{22} - 16q_0q_{23} - 128q_0q_{24} - 16q_0q_{25} - 16q_0q_{26} - 16q_0q_{27} + 24q_0q_3 + 64q_0q_4 + 8q_0q_5 + 8q_0q_6 + 8q_0q_7 + 64q_0q_8 + 8q_0q_9 - 36q_0 + 8q_1q_{10} + 8q_1q_{11} + 8q_1q_{12} + 64q_1q_{13} + 8q_1q_{14} + 8q_1q_{15} - 16q_1q_{16} - 128q_1q_{17} - 16q_1q_{18} - 16q_1q_{19} + 24q_1q_2 - 16q_1q_{20} - 128q_1q_{21} - 16q_1q_{22} - 16q_1q_{23} - 16q_1q_{24} - 128q_1q_{25} - 16q_1q_{26} - 16q_1q_{27} + 24q_1q_3 + 8q_1q_4 + 64q_1q_5 + 8q_1q_6 + 8q_1q_7 + 8q_1q_8 + 64q_1q_9 - 36q_1 + 24q_{10}q_{11} + 8q_{10}q_{12} + 8q_{10}q_{13} + 64q_{10}q_{14} + 8q_{10}q_{15} + 64q_{10}q_2 - 16q_{10}q_{20} - 16q_{10}q_{21} - 128q_{10}q_{22} - 16q_{10}q_{23} - 16q_{10}q_{28} - 16q_{10}q_{29} + 8q_{10}q_3 - 128q_{10}q_{30} - 16q_{10}q_{31} - 16q_{10}q_{36} - 16q_{10}q_{37} - 128q_{10}q_{38} - 16q_{10}q_{39} + 8q_{10}q_4 + 8q_{10}q_5 + 64q_{10}q_6 + 8q_{10}q_7 + 24q_{10}q_8 + 24q_{10}q_9 - 36q_{10} + 8q_{11}q_{12} + 8q_{11}q_{13} + 8q_{11}q_{14} + 64q_{11}q_{15} + 8q_{11}q_2 - 16q_{11}q_{20} - 16q_{11}q_{21} - 16q_{11}q_{22} - 128q_{11}q_{23} - 16q_{11}q_{28} - 16q_{11}q_{29} + 64q_{11}q_3 - 16q_{11}q_{30} - 128q_{11}q_{31} - 16q_{11}q_{36} - 16q_{11}q_{37} - 16q_{11}q_{38} - 128q_{11}q_{39} + 8q_{11}q_4 + 8q_{11}q_5 + 8q_{11}q_6 + 64q_{11}q_7 + 24q_{11}q_8 + 24q_{11}q_9 - 36q_{11} + 24q_{12}q_{13} + 24q_{12}q_{14} + 24q_{12}q_{15} + 8q_{12}q_2 - 128q_{12}q_{24} - 16q_{12}q_{25} - 16q_{12}q_{26} - 16q_{12}q_{27} + 8q_{12}q_3 - 128q_{12}q_{32} - 16q_{12}q_{33} - 16q_{12}q_{34} - 16q_{12}q_{35} - 128q_{12}q_{36} - 16q_{12}q_{37} - 16q_{12}q_{38} - 16q_{12}q_{39} + 64q_{12}q_4 + 8q_{12}q_5 + 8q_{12}q_6 + 8q_{12}q_7 + 64q_{12}q_8 + 8q_{12}q_9 - 36q_{12} + 24q_{13}q_{14} + 24q_{13}q_{15} + 8q_{13}q_2 - 16q_{13}q_{24} - 128q_{13}q_{25} - 16q_{13}q_{26} - 16q_{13}q_{27} + 8q_{13}q_3 - 16q_{13}q_{32} - 128q_{13}q_{33} - 16q_{13}q_{34} - 16q_{13}q_{35} - 16q_{13}q_{36} - 128q_{13}q_{37} - 16q_{13}q_{38} - 16q_{13}q_{39} + 8q_{13}q_4 + 64q_{13}q_5 + 8q_{13}q_6 + 8q_{13}q_7 + 8q_{13}q_8 + 64q_{13}q_9 - 36q_{13} + 24q_{14}q_{15} + 64q_{14}q_2 - 16q_{14}q_{24} - 16q_{14}q_{25} - 128q_{14}q_{26} - 16q_{14}q_{27} + 8q_{14}q_3 - 16q_{14}q_{32} - 16q_{14}q_{33} - 128q_{14}q_{34} - 16q_{14}q_{35} - 16q_{14}q_{36} - 16q_{14}q_{37} - 128q_{14}q_{38} - 16q_{14}q_{39} + 8q_{14}q_4 + 8q_{14}q_5 + 64q_{14}q_6 + 8q_{14}q_7 + 8q_{14}q_8 + 8q_{14}q_9 - 36q_{14} + 8q_{15}q_2 - 16q_{15}q_{24} - 16q_{15}q_{25} - 16q_{15}q_{26} - 128q_{15}q_{27} + 64q_{15}q_3 - 16q_{15}q_{32} - 16q_{15}q_{33} - 16q_{15}q_{34} - 128q_{15}q_{35} - 16q_{15}q_{36} - 16q_{15}q_{37} - 16q_{15}q_{38} - 128q_{15}q_{39} + 8q_{15}q_4 + 8q_{15}q_5 + 8q_{15}q_6 + 64q_{15}q_7 + 8q_{15}q_8 + 8q_{15}q_9 - 36q_{15} + 32q_{16}q_{17} + 32q_{16}q_{18} + 32q_{16}q_{19} - 16q_{16}q_2 - 16q_{16}q_3 - 128q_{16}q_4 - 16q_{16}q_5 - 16q_{16}q_6 - 16q_{16}q_7 + 216q_{16} + 32q_{17}q_{18} + 32q_{17}q_{19} - 16q_{17}q_2 - 16q_{17}q_3 - 16q_{17}q_4 - 128q_{17}q_5 - 16q_{17}q_6 - 16q_{17}q_7 + 216q_{17} + 32q_{18}q_{19} - 128q_{18}q_2 - 16q_{18}q_3 - 16q_{18}q_4 - 16q_{18}q_5 - 128q_{18}q_6 - 16q_{18}q_7 + 216q_{18} - 16q_{19}q_2 - 128q_{19}q_3 - 16q_{19}q_4 - 16q_{19}q_5 - 16q_{19}q_6 - 128q_{19}q_7 + 216q_{19} - 16q_2q_{20} - 16q_2q_{21} - 128q_2q_{22} - 16q_2q_{23} - 16q_2q_{24} - 16q_2q_{25} - 128q_2q_{26} - 16q_2q_{27} + 24q_2q_3 + 8q_2q_4 + 8q_2q_5 + 64q_2q_6 + 8q_2q_7 + 8q_2q_8 + 8q_2q_9 - 36q_2 + 32q_{20}q_{21} + 32q_{20}q_{22} + 32q_{20}q_{23} - 16q_{20}q_3 - 128q_{20}q_8 - 16q_{20}q_9 + 216q_{20} + 32q_{21}q_{22} + 32q_{21}q_{23} - 16q_{21}q_3 - 16q_{21}q_8 - 128q_{21}q_9 + 216q_{21} + 32q_{22}q_{23} - 16q_{22}q_3 - 16q_{22}q_8 - 16q_{22}q_9 + 216q_{22} - 128q_{23}q_3 - 16q_{23}q_8 - 16q_{23}q_9 + 216q_{23} + 32q_{24}q_{25} + 32q_{24}q_{26} + 32q_{24}q_{27} - 16q_{24}q_3 + 216q_{24} + 32q_{25}q_{26} + 32q_{25}q_{27} - 16q_{25}q_3 + 216q_{25} + 32q_{26}q_{27} - 16q_{26}q_3 + 216q_{26} - 128q_{27}q_3 + 216q_{27} + 32q_{28}q_{29} + 32q_{28}q_{30} + 32q_{28}q_{31} - 128q_{28}q_4 - 16q_{28}q_5 - 16q_{28}q_6 - 16q_{28}q_7 - 128q_{28}q_8 - 16q_{28}q_9 + 216q_{28} + 32q_{29}q_{30} + 32q_{29}q_{31} - 16q_{29}q_4 - 128q_{29}q_5 - 16q_{29}q_6 - 16q_{29}q_7 - 16q_{29}q_8 - 128q_{29}q_9 + 216q_{29} + 8q_3q_4 + 8q_3q_5 + 8q_3q_6 + 64q_3q_7 + 8q_3q_8 + 8q_3q_9 - 36q_3 + 32q_{30}q_{31} - 16q_{30}q_4 - 16q_{30}q_5 - 128q_{30}q_6 - 16q_{30}q_7 - 16q_{30}q_8 - 16q_{30}q_9 + 216q_{30} - 16q_{31}q_4 - 16q_{31}q_5 - 16q_{31}q_6 - 128q_{31}q_7 - 16q_{31}q_8 - 16q_{31}q_9 + 216q_{31} + 32q_{32}q_{33} + 32q_{32}q_{34} + 32q_{32}q_{35} - 128q_{32}q_4 - 16q_{32}q_5 - 16q_{32}q_6 - 16q_{32}q_7 + 216q_{32} + 32q_{33}q_{34} + 32q_{33}q_{35} - 16q_{33}q_4 - 128q_{33}q_5 - 16q_{33}q_6 - 16q_{33}q_7 + 216q_{33} + 32q_{34}q_{35} - 16q_{34}q_4 - 16q_{34}q_5 - 128q_{34}q_6 - 16q_{34}q_7 + 216q_{34} - 16q_{35}q_4 - 16q_{35}q_5 - 16q_{35}q_6 - 128q_{35}q_7 + 216q_{35} + 32q_{36}q_{37} + 32q_{36}q_{38} + 32q_{36}q_{39} - 128q_{36}q_8 - 16q_{36}q_9 + 216q_{36} + 32q_{37}q_{38} + 32q_{37}q_{39} - 16q_{37}q_8 - 128q_{37}q_9 + 216q_{37} + 32q_{38}q_{39} - 16q_{38}q_8 - 16q_{38}q_9 + 216q_{38} - 16q_{39}q_8 - 16q_{39}q_9 + 216q_{39} + 24q_4q_5 + 24q_4q_6 + 24q_4q_7 + 64q_4q_8 + 8q_4q_9 - 36q_4 + 24q_5q_6 + 24q_5q_7 + 8q_5q_8 + 64q_5q_9 - 36q_5 + 24q_6q_7 + 8q_6q_8 + 8q_6q_9 - 36q_6 + 8q_7q_8 + 8q_7q_9 - 36q_7 + 24q_8q_9 - 36q_8 - 36q_9 + 96$$

A complete expression of Eq.(S.17)

$$E_2(s) = 6s_0s_1 + 2s_0s_{10} + 2s_0s_{11} + 16s_0s_{12} + 2s_0s_{13} + 2s_0s_{14} + 2s_0s_{15} - 32s_0s_{16} - 4s_0s_{17} - 4s_0s_{18} - 4s_0s_{19} + 6s_0s_2 - 32s_0s_{20} - 4s_0s_{21} - 4s_0s_{22} - 4s_0s_{23} - 32s_0s_{24} - 4s_0s_{25} - 4s_0s_{26} - 4s_0s_{27} + 6s_0s_3 + 16s_0s_4 + 2s_0s_5 + 2s_0s_6 + 2s_0s_7 + 16s_0s_8 + 2s_0s_9 + 66s_0 + 2s_1s_{10} + 2s_1s_{11} + 2s_1s_{12} + 16s_1s_{13} + 2s_1s_{14} + 2s_1s_{15} - 4s_1s_{16} - 32s_1s_{17} - 4s_1s_{18} - 4s_1s_{19} + 6s_1s_2 - 4s_1s_{20} - 32s_1s_{21} - 4s_1s_{22} - 4s_1s_{23} - 4s_1s_{24} - 32s_1s_{25} - 4s_1s_{26} - 4s_1s_{27} + 6s_1s_3 + 2s_1s_4 + 16s_1s_5 + 2s_1s_6 + 2s_1s_7 + 2s_1s_8 + 16s_1s_9 + 66s_1 + 6s_{10}s_{11} + 2s_{10}s_{12} + 2s_{10}s_{13} + 16s_{10}s_{14} + 2s_{10}s_{15} + 16s_{10}s_2 - 4s_{10}s_{20} - 4s_{10}s_{21} - 32s_{10}s_{22} - 4s_{10}s_{23} - 4s_{10}s_{28} - 4s_{10}s_{29} + 2s_{10}s_3 - 32s_{10}s_{30} - 4s_{10}s_{31} - 4s_{10}s_{36} - 4s_{10}s_{37} - 32s_{10}s_{38} - 4s_{10}s_{39} +$$

$$2s_{10}s_4 + 2s_{10}s_5 + 16s_{10}s_6 + 2s_{10}s_7 + 6s_{10}s_8 + 6s_{10}s_9 + 66s_{10} + 2s_{11}s_{12} + 2s_{11}s_{13} + 2s_{11}s_{14} + 16s_{11}s_{15} + 2s_{11}s_2 -$$
$$4s_{11}s_{20} - 4s_{11}s_{21} - 4s_{11}s_{22} - 32s_{11}s_{23} - 4s_{11}s_{28} - 4s_{11}s_{29} + 16s_{11}s_3 - 4s_{11}s_{30} - 32s_{11}s_{31} - 4s_{11}s_{36} - 4s_{11}s_{37} -$$
$$4s_{11}s_{38} - 32s_{11}s_{39} + 2s_{11}s_4 + 2s_{11}s_5 + 2s_{11}s_6 + 16s_{11}s_7 + 6s_{11}s_8 + 6s_{11}s_9 + 66s_{11} + 6s_{12}s_{13} + 6s_{12}s_{14} + 6s_{12}s_{15} +$$
$$2s_{12}s_2 - 32s_{12}s_{24} - 4s_{12}s_{25} - 4s_{12}s_{26} - 4s_{12}s_{27} + 2s_{12}s_3 - 32s_{12}s_{32} - 4s_{12}s_{33} - 4s_{12}s_{34} - 4s_{12}s_{35} - 32s_{12}s_{36} -$$
$$4s_{12}s_{37} - 4s_{12}s_{38} - 4s_{12}s_{39} + 16s_{12}s_4 + 2s_{12}s_5 + 2s_{12}s_6 + 2s_{12}s_7 + 16s_{12}s_8 + 2s_{12}s_9 + 66s_{12} + 6s_{13}s_{14} + 6s_{13}s_{15} +$$
$$2s_{13}s_2 - 4s_{13}s_{24} - 32s_{13}s_{25} - 4s_{13}s_{26} - 4s_{13}s_{27} + 2s_{13}s_3 - 4s_{13}s_{32} - 32s_{13}s_{33} - 4s_{13}s_{34} - 4s_{13}s_{35} - 4s_{13}s_{36} -$$
$$32s_{13}s_{37} - 4s_{13}s_{38} - 4s_{13}s_{39} + 2s_{13}s_4 + 16s_{13}s_5 + 2s_{13}s_6 + 2s_{13}s_7 + 2s_{13}s_8 + 16s_{13}s_9 + 66s_{13} + 6s_{14}s_{15} + 16s_{14}s_2 -$$
$$4s_{14}s_{24} - 4s_{14}s_{25} - 32s_{14}s_{26} - 4s_{14}s_{27} + 2s_{14}s_3 - 4s_{14}s_{32} - 4s_{14}s_{33} - 32s_{14}s_{34} - 4s_{14}s_{35} - 4s_{14}s_{36} - 4s_{14}s_{37} -$$
$$32s_{14}s_{38} - 4s_{14}s_{39} + 2s_{14}s_4 + 2s_{14}s_5 + 16s_{14}s_6 + 2s_{14}s_7 + 2s_{14}s_8 + 2s_{14}s_9 + 66s_{14} + 2s_{15}s_2 - 4s_{15}s_{24} - 4s_{15}s_{25} -$$
$$4s_{15}s_{26} - 32s_{15}s_{27} + 16s_{15}s_3 - 4s_{15}s_{32} - 4s_{15}s_{33} - 4s_{15}s_{34} - 32s_{15}s_{35} - 4s_{15}s_{36} - 4s_{15}s_{37} - 4s_{15}s_{38} - 32s_{15}s_{39} +$$
$$2s_{15}s_4 + 2s_{15}s_5 + 2s_{15}s_6 + 16s_{15}s_7 + 2s_{15}s_8 + 2s_{15}s_9 + 66s_{15} + 8s_{16}s_{17} + 8s_{16}s_{18} + 8s_{16}s_{19} - 4s_{16}s_2 - 4s_{16}s_3 -$$
$$32s_{16}s_4 - 4s_{16}s_5 - 4s_{16}s_6 - 4s_{16}s_7 - 44s_{16} + 8s_{17}s_{18} + 8s_{17}s_{19} - 4s_{17}s_2 - 4s_{17}s_3 - 4s_{17}s_4 - 32s_{17}s_5 - 4s_{17}s_6 -$$
$$4s_{17}s_7 - 44s_{17} + 8s_{18}s_{19} - 32s_{18}s_2 - 4s_{18}s_3 - 4s_{18}s_4 - 4s_{18}s_5 - 32s_{18}s_6 - 4s_{18}s_7 - 44s_{18} - 4s_{19}s_2 - 32s_{19}s_3 -$$
$$4s_{19}s_4 - 4s_{19}s_5 - 4s_{19}s_6 - 32s_{19}s_7 - 44s_{19} - 4s_2s_{20} - 4s_2s_{21} - 32s_2s_{22} - 4s_2s_{23} - 4s_2s_{24} - 4s_2s_{25} - 32s_2s_{26} -$$
$$4s_2s_{27} + 6s_2s_3 + 2s_2s_4 + 2s_2s_5 + 16s_2s_6 + 2s_2s_7 + 2s_2s_8 + 2s_2s_9 + 66s_2 + 8s_{20}s_{21} + 8s_{20}s_{22} + 8s_{20}s_{23} - 4s_{20}s_3 -$$
$$32s_{20}s_8 - 4s_{20}s_9 - 44s_{20} + 8s_{21}s_{22} + 8s_{21}s_{23} - 4s_{21}s_3 - 4s_{21}s_8 - 32s_{21}s_9 - 44s_{21} + 8s_{22}s_{23} - 4s_{22}s_3 - 4s_{22}s_8 -$$
$$4s_{22}s_9 - 44s_{22} - 32s_{23}s_3 - 4s_{23}s_8 - 4s_{23}s_9 - 44s_{23} + 8s_{24}s_{25} + 8s_{24}s_{26} + 8s_{24}s_{27} - 4s_{24}s_3 - 44s_{24} + 8s_{25}s_{26} +$$
$$8s_{25}s_{27} - 4s_{25}s_3 - 44s_{25} + 8s_{26}s_{27} - 4s_{26}s_3 - 44s_{26} - 32s_{27}s_3 - 44s_{27} + 8s_{28}s_{29} + 8s_{28}s_{30} + 8s_{28}s_{31} - 32s_{28}s_4 -$$
$$4s_{28}s_5 - 4s_{28}s_6 - 4s_{28}s_7 - 32s_{28}s_8 - 4s_{28}s_9 - 44s_{28} + 8s_{29}s_{30} + 8s_{29}s_{31} - 4s_{29}s_4 - 32s_{29}s_5 - 4s_{29}s_6 - 4s_{29}s_7 -$$
$$4s_{29}s_8 - 32s_{29}s_9 - 44s_{29} + 2s_3s_4 + 2s_3s_5 + 2s_3s_6 + 16s_3s_7 + 2s_3s_8 + 2s_3s_9 + 66s_3 + 8s_{30}s_{31} - 4s_{30}s_4 - 4s_{30}s_5 -$$
$$32s_{30}s_6 - 4s_{30}s_7 - 4s_{30}s_8 - 4s_{30}s_9 - 44s_{30} - 4s_{31}s_4 - 4s_{31}s_5 - 4s_{31}s_6 - 32s_{31}s_7 - 4s_{31}s_8 - 4s_{31}s_9 - 44s_{31} + 8s_{32}s_{33} +$$
$$8s_{32}s_{34} + 8s_{32}s_{35} - 32s_{32}s_4 - 4s_{32}s_5 - 4s_{32}s_6 - 4s_{32}s_7 - 44s_{32} + 8s_{33}s_{34} + 8s_{33}s_{35} - 4s_{33}s_4 - 32s_{33}s_5 - 4s_{33}s_6 -$$
$$4s_{33}s_7 - 44s_{33} + 8s_{34}s_{35} - 4s_{34}s_4 - 4s_{34}s_5 - 32s_{34}s_6 - 4s_{34}s_7 - 44s_{34} - 4s_{35}s_4 - 4s_{35}s_5 - 4s_{35}s_6 - 32s_{35}s_7 - 44s_{35} +$$
$$8s_{36}s_{37} + 8s_{36}s_{38} + 8s_{36}s_{39} - 32s_{36}s_8 - 4s_{36}s_9 - 44s_{36} + 8s_{37}s_{38} + 8s_{37}s_{39} - 4s_{37}s_8 - 32s_{37}s_9 - 44s_{37} + 8s_{38}s_{39} -$$
$$4s_{38}s_8 - 4s_{38}s_9 - 44s_{38} - 4s_{39}s_8 - 4s_{39}s_9 - 44s_{39} + 6s_4s_5 + 6s_4s_6 + 6s_4s_7 + 16s_4s_8 + 2s_4s_9 + 66s_4 + 6s_5s_6 +$$
$$6s_5s_7 + 2s_5s_8 + 16s_5s_9 + 66s_5 + 6s_6s_7 + 2s_6s_8 + 2s_6s_9 + 66s_6 + 2s_7s_8 + 2s_7s_9 + 66s_7 + 6s_8s_9 + 66s_8 + 66s_9 + 1,248$$

A complete expression of Eq.(12)

$$E_2(\hat{\sigma}^z) = 6\hat{\sigma}^z_0\hat{\sigma}^z_1 + 2\hat{\sigma}^z_0\hat{\sigma}^z_{10} + 2\hat{\sigma}^z_0\hat{\sigma}^z_{11} + 16\hat{\sigma}^z_0\hat{\sigma}^z_{12} + 2\hat{\sigma}^z_0\hat{\sigma}^z_{13} + 2\hat{\sigma}^z_0\hat{\sigma}^z_{14} + 2\hat{\sigma}^z_0\hat{\sigma}^z_{15} - 32\hat{\sigma}^z_0\hat{\sigma}^z_{16} - 4\hat{\sigma}^z_0\hat{\sigma}^z_{17} - 4\hat{\sigma}^z_0\hat{\sigma}^z_{18} -$$
$$4\hat{\sigma}^z_0\hat{\sigma}^z_{19} + 6\hat{\sigma}^z_0\hat{\sigma}^z_2 - 32\hat{\sigma}^z_0\hat{\sigma}^z_{20} - 4\hat{\sigma}^z_0\hat{\sigma}^z_{21} - 4\hat{\sigma}^z_0\hat{\sigma}^z_{22} - 4\hat{\sigma}^z_0\hat{\sigma}^z_{23} - 32\hat{\sigma}^z_0\hat{\sigma}^z_{24} - 4\hat{\sigma}^z_0\hat{\sigma}^z_{25} - 4\hat{\sigma}^z_0\hat{\sigma}^z_{26} - 4\hat{\sigma}^z_0\hat{\sigma}^z_{27} + 6\hat{\sigma}^z_0\hat{\sigma}^z_3 +$$
$$16\hat{\sigma}^z_0\hat{\sigma}^z_4 + 2\hat{\sigma}^z_0\hat{\sigma}^z_5 + 2\hat{\sigma}^z_0\hat{\sigma}^z_6 + 2\hat{\sigma}^z_0\hat{\sigma}^z_7 + 16\hat{\sigma}^z_0\hat{\sigma}^z_8 + 2\hat{\sigma}^z_0\hat{\sigma}^z_9 + 66\hat{\sigma}^z_0 + 2\hat{\sigma}^z_1\hat{\sigma}^z_{10} + 2\hat{\sigma}^z_1\hat{\sigma}^z_{11} + 2\hat{\sigma}^z_1\hat{\sigma}^z_{12} + 16\hat{\sigma}^z_1\hat{\sigma}^z_{13} +$$
$$2\hat{\sigma}^z_1\hat{\sigma}^z_{14} + 2\hat{\sigma}^z_1\hat{\sigma}^z_{15} - 4\hat{\sigma}^z_1\hat{\sigma}^z_{16} - 32\hat{\sigma}^z_1\hat{\sigma}^z_{17} - 4\hat{\sigma}^z_1\hat{\sigma}^z_{18} - 4\hat{\sigma}^z_1\hat{\sigma}^z_{19} + 6\hat{\sigma}^z_1\hat{\sigma}^z_2 - 4\hat{\sigma}^z_1\hat{\sigma}^z_{20} - 32\hat{\sigma}^z_1\hat{\sigma}^z_{21} - 4\hat{\sigma}^z_1\hat{\sigma}^z_{22} - 4\hat{\sigma}^z_1\hat{\sigma}^z_{23} -$$
$$4\hat{\sigma}^z_1\hat{\sigma}^z_{24} - 32\hat{\sigma}^z_1\hat{\sigma}^z_{25} - 4\hat{\sigma}^z_1\hat{\sigma}^z_{26} - 4\hat{\sigma}^z_1\hat{\sigma}^z_{27} + 6\hat{\sigma}^z_1\hat{\sigma}^z_3 + 2\hat{\sigma}^z_1\hat{\sigma}^z_4 + 16\hat{\sigma}^z_1\hat{\sigma}^z_5 + 2\hat{\sigma}^z_1\hat{\sigma}^z_6 + 2\hat{\sigma}^z_1\hat{\sigma}^z_7 + 2\hat{\sigma}^z_1\hat{\sigma}^z_8 + 16\hat{\sigma}^z_1\hat{\sigma}^z_9 +$$
$$66\hat{\sigma}^z_1 + 6\hat{\sigma}^z_{10}\hat{\sigma}^z_{11} + 2\hat{\sigma}^z_{10}\hat{\sigma}^z_{12} + 2\hat{\sigma}^z_{10}\hat{\sigma}^z_{13} + 16\hat{\sigma}^z_{10}\hat{\sigma}^z_{14} + 2\hat{\sigma}^z_{10}\hat{\sigma}^z_{15} + 16\hat{\sigma}^z_{10}\hat{\sigma}^z_2 - 4\hat{\sigma}^z_{10}\hat{\sigma}^z_{20} - 4\hat{\sigma}^z_{10}\hat{\sigma}^z_{21} - 32\hat{\sigma}^z_{10}\hat{\sigma}^z_{22} -$$
$$4\hat{\sigma}^z_{10}\hat{\sigma}^z_{23} - 4\hat{\sigma}^z_{10}\hat{\sigma}^z_{28} - 4\hat{\sigma}^z_{10}\hat{\sigma}^z_{29} + 2\hat{\sigma}^z_{10}\hat{\sigma}^z_3 - 32\hat{\sigma}^z_{10}\hat{\sigma}^z_{30} - 4\hat{\sigma}^z_{10}\hat{\sigma}^z_{31} - 4\hat{\sigma}^z_{10}\hat{\sigma}^z_{36} - 4\hat{\sigma}^z_{10}\hat{\sigma}^z_{37} - 32\hat{\sigma}^z_{10}\hat{\sigma}^z_{38} - 4\hat{\sigma}^z_{10}\hat{\sigma}^z_{39} +$$
$$2\hat{\sigma}^z_{10}\hat{\sigma}^z_4 + 2\hat{\sigma}^z_{10}\hat{\sigma}^z_5 + 16\hat{\sigma}^z_{10}\hat{\sigma}^z_6 + 2\hat{\sigma}^z_{10}\hat{\sigma}^z_7 + 6\hat{\sigma}^z_{10}\hat{\sigma}^z_8 + 6\hat{\sigma}^z_{10}\hat{\sigma}^z_9 + 66\hat{\sigma}^z_{10} + 2\hat{\sigma}^z_{11}\hat{\sigma}^z_{12} + 2\hat{\sigma}^z_{11}\hat{\sigma}^z_{13} + 2\hat{\sigma}^z_{11}\hat{\sigma}^z_{14} + 16\hat{\sigma}^z_{11}\hat{\sigma}^z_{15} +$$
$$2\hat{\sigma}^z_{11}\hat{\sigma}^z_2 - 4\hat{\sigma}^z_{11}\hat{\sigma}^z_{20} - 4\hat{\sigma}^z_{11}\hat{\sigma}^z_{21} - 4\hat{\sigma}^z_{11}\hat{\sigma}^z_{22} - 32\hat{\sigma}^z_{11}\hat{\sigma}^z_{23} - 4\hat{\sigma}^z_{11}\hat{\sigma}^z_{28} - 4\hat{\sigma}^z_{11}\hat{\sigma}^z_{29} + 16\hat{\sigma}^z_{11}\hat{\sigma}^z_3 - 4\hat{\sigma}^z_{11}\hat{\sigma}^z_{30} - 32\hat{\sigma}^z_{11}\hat{\sigma}^z_{31} -$$
$$4\hat{\sigma}^z_{11}\hat{\sigma}^z_{36} - 4\hat{\sigma}^z_{11}\hat{\sigma}^z_{37} - 4\hat{\sigma}^z_{11}\hat{\sigma}^z_{38} - 32\hat{\sigma}^z_{11}\hat{\sigma}^z_{39} + 2\hat{\sigma}^z_{11}\hat{\sigma}^z_4 + 2\hat{\sigma}^z_{11}\hat{\sigma}^z_5 + 2\hat{\sigma}^z_{11}\hat{\sigma}^z_6 + 16\hat{\sigma}^z_{11}\hat{\sigma}^z_7 + 6\hat{\sigma}^z_{11}\hat{\sigma}^z_8 + 6\hat{\sigma}^z_{11}\hat{\sigma}^z_9 + 66\hat{\sigma}^z_{11} +$$
$$6\hat{\sigma}^z_{12}\hat{\sigma}^z_{13} + 6\hat{\sigma}^z_{12}\hat{\sigma}^z_{14} + 6\hat{\sigma}^z_{12}\hat{\sigma}^z_{15} + 2\hat{\sigma}^z_{12}\hat{\sigma}^z_2 - 32\hat{\sigma}^z_{12}\hat{\sigma}^z_{24} - 4\hat{\sigma}^z_{12}\hat{\sigma}^z_{25} - 4\hat{\sigma}^z_{12}\hat{\sigma}^z_{26} - 4\hat{\sigma}^z_{12}\hat{\sigma}^z_{27} + 2\hat{\sigma}^z_{12}\hat{\sigma}^z_3 - 32\hat{\sigma}^z_{12}\hat{\sigma}^z_{32} -$$
$$4\hat{\sigma}^z_{12}\hat{\sigma}^z_{33} - 4\hat{\sigma}^z_{12}\hat{\sigma}^z_{34} - 4\hat{\sigma}^z_{12}\hat{\sigma}^z_{35} - 32\hat{\sigma}^z_{12}\hat{\sigma}^z_{36} - 4\hat{\sigma}^z_{12}\hat{\sigma}^z_{37} - 4\hat{\sigma}^z_{12}\hat{\sigma}^z_{38} - 4\hat{\sigma}^z_{12}\hat{\sigma}^z_{39} + 16\hat{\sigma}^z_{12}\hat{\sigma}^z_4 + 2\hat{\sigma}^z_{12}\hat{\sigma}^z_5 + 2\hat{\sigma}^z_{12}\hat{\sigma}^z_6 +$$
$$2\hat{\sigma}^z_{12}\hat{\sigma}^z_7 + 16\hat{\sigma}^z_{12}\hat{\sigma}^z_8 + 2\hat{\sigma}^z_{12}\hat{\sigma}^z_9 + 66\hat{\sigma}^z_{12} + 6\hat{\sigma}^z_{13}\hat{\sigma}^z_{14} + 6\hat{\sigma}^z_{13}\hat{\sigma}^z_{15} + 2\hat{\sigma}^z_{13}\hat{\sigma}^z_2 - 4\hat{\sigma}^z_{13}\hat{\sigma}^z_{24} - 32\hat{\sigma}^z_{13}\hat{\sigma}^z_{25} - 4\hat{\sigma}^z_{13}\hat{\sigma}^z_{26} -$$
$$4\hat{\sigma}^z_{13}\hat{\sigma}^z_{27} + 2\hat{\sigma}^z_{13}\hat{\sigma}^z_3 - 4\hat{\sigma}^z_{13}\hat{\sigma}^z_{32} - 32\hat{\sigma}^z_{13}\hat{\sigma}^z_{33} - 4\hat{\sigma}^z_{13}\hat{\sigma}^z_{34} - 4\hat{\sigma}^z_{13}\hat{\sigma}^z_{35} - 4\hat{\sigma}^z_{13}\hat{\sigma}^z_{36} - 32\hat{\sigma}^z_{13}\hat{\sigma}^z_{37} - 4\hat{\sigma}^z_{13}\hat{\sigma}^z_{38} - 4\hat{\sigma}^z_{13}\hat{\sigma}^z_{39} +$$
$$2\hat{\sigma}^z_{13}\hat{\sigma}^z_4 + 16\hat{\sigma}^z_{13}\hat{\sigma}^z_5 + 2\hat{\sigma}^z_{13}\hat{\sigma}^z_6 + 2\hat{\sigma}^z_{13}\hat{\sigma}^z_7 + 2\hat{\sigma}^z_{13}\hat{\sigma}^z_8 + 16\hat{\sigma}^z_{13}\hat{\sigma}^z_9 + 66\hat{\sigma}^z_{13} + 6\hat{\sigma}^z_{14}\hat{\sigma}^z_{15} + 16\hat{\sigma}^z_{14}\hat{\sigma}^z_2 - 4\hat{\sigma}^z_{14}\hat{\sigma}^z_{24} - 4\hat{\sigma}^z_{14}\hat{\sigma}^z_{25} -$$
$$32\hat{\sigma}^z_{14}\hat{\sigma}^z_{26} - 4\hat{\sigma}^z_{14}\hat{\sigma}^z_{27} + 2\hat{\sigma}^z_{14}\hat{\sigma}^z_3 - 4\hat{\sigma}^z_{14}\hat{\sigma}^z_{32} - 4\hat{\sigma}^z_{14}\hat{\sigma}^z_{33} - 32\hat{\sigma}^z_{14}\hat{\sigma}^z_{34} - 4\hat{\sigma}^z_{14}\hat{\sigma}^z_{35} - 4\hat{\sigma}^z_{14}\hat{\sigma}^z_{36} - 4\hat{\sigma}^z_{14}\hat{\sigma}^z_{37} - 32\hat{\sigma}^z_{14}\hat{\sigma}^z_{38} -$$
$$4\hat{\sigma}^z_{14}\hat{\sigma}^z_{39} + 2\hat{\sigma}^z_{14}\hat{\sigma}^z_4 + 2\hat{\sigma}^z_{14}\hat{\sigma}^z_5 + 16\hat{\sigma}^z_{14}\hat{\sigma}^z_6 + 2\hat{\sigma}^z_{14}\hat{\sigma}^z_7 + 2\hat{\sigma}^z_{14}\hat{\sigma}^z_8 + 2\hat{\sigma}^z_{14}\hat{\sigma}^z_9 + 66\hat{\sigma}^z_{14} + 2\hat{\sigma}^z_{15}\hat{\sigma}^z_2 - 4\hat{\sigma}^z_{15}\hat{\sigma}^z_{24} - 4\hat{\sigma}^z_{15}\hat{\sigma}^z_{25} -$$
$$4\hat{\sigma}^z_{15}\hat{\sigma}^z_{26} - 32\hat{\sigma}^z_{15}\hat{\sigma}^z_{27} + 16\hat{\sigma}^z_{15}\hat{\sigma}^z_3 - 4\hat{\sigma}^z_{15}\hat{\sigma}^z_{32} - 4\hat{\sigma}^z_{15}\hat{\sigma}^z_{33} - 4\hat{\sigma}^z_{15}\hat{\sigma}^z_{34} - 32\hat{\sigma}^z_{15}\hat{\sigma}^z_{35} - 4\hat{\sigma}^z_{15}\hat{\sigma}^z_{36} - 4\hat{\sigma}^z_{15}\hat{\sigma}^z_{37} - 4\hat{\sigma}^z_{15}\hat{\sigma}^z_{38} -$$
$$32\hat{\sigma}^z_{15}\hat{\sigma}^z_{39} + 2\hat{\sigma}^z_{15}\hat{\sigma}^z_4 + 2\hat{\sigma}^z_{15}\hat{\sigma}^z_5 + 2\hat{\sigma}^z_{15}\hat{\sigma}^z_6 + 16\hat{\sigma}^z_{15}\hat{\sigma}^z_7 + 2\hat{\sigma}^z_{15}\hat{\sigma}^z_8 + 2\hat{\sigma}^z_{15}\hat{\sigma}^z_9 + 66\hat{\sigma}^z_{15} + 8\hat{\sigma}^z_{16}\hat{\sigma}^z_{17} + 8\hat{\sigma}^z_{16}\hat{\sigma}^z_{18} + 8\hat{\sigma}^z_{16}\hat{\sigma}^z_{19} -$$
$$4\hat{\sigma}^z_{16}\hat{\sigma}^z_2 - 4\hat{\sigma}^z_{16}\hat{\sigma}^z_3 - 32\hat{\sigma}^z_{16}\hat{\sigma}^z_4 - 4\hat{\sigma}^z_{16}\hat{\sigma}^z_5 - 4\hat{\sigma}^z_{16}\hat{\sigma}^z_6 - 4\hat{\sigma}^z_{16}\hat{\sigma}^z_7 - 44\hat{\sigma}^z_{16} + 8\hat{\sigma}^z_{17}\hat{\sigma}^z_{18} + 8\hat{\sigma}^z_{17}\hat{\sigma}^z_{19} - 4\hat{\sigma}^z_{17}\hat{\sigma}^z_2 - 4\hat{\sigma}^z_{17}\hat{\sigma}^z_3 -$$

$4\hat{\sigma}_{17}^{z}\hat{\sigma}_{4}^{z}-32\hat{\sigma}_{17}^{z}\hat{\sigma}_{5}^{z}-4\hat{\sigma}_{17}^{z}\hat{\sigma}_{6}^{z}-4\hat{\sigma}_{17}^{z}\hat{\sigma}_{7}^{z}-44\hat{\sigma}_{17}^{z}+8\hat{\sigma}_{18}^{z}\hat{\sigma}_{19}^{z}-32\hat{\sigma}_{18}^{z}\hat{\sigma}_{2}^{z}-4\hat{\sigma}_{18}^{z}\hat{\sigma}_{3}^{z}-4\hat{\sigma}_{18}^{z}\hat{\sigma}_{4}^{z}-4\hat{\sigma}_{18}^{z}\hat{\sigma}_{5}^{z}-32\hat{\sigma}_{18}^{z}\hat{\sigma}_{6}^{z}-$
$4\hat{\sigma}_{18}^{z}\hat{\sigma}_{7}^{z}-44\hat{\sigma}_{18}^{z}-4\hat{\sigma}_{19}^{z}\hat{\sigma}_{2}^{z}-32\hat{\sigma}_{19}^{z}\hat{\sigma}_{3}^{z}-4\hat{\sigma}_{19}^{z}\hat{\sigma}_{4}^{z}-4\hat{\sigma}_{19}^{z}\hat{\sigma}_{5}^{z}-4\hat{\sigma}_{19}^{z}\hat{\sigma}_{6}^{z}-32\hat{\sigma}_{19}^{z}\hat{\sigma}_{7}^{z}-44\hat{\sigma}_{19}^{z}-4\hat{\sigma}_{2}^{z}\hat{\sigma}_{20}^{z}-4\hat{\sigma}_{2}^{z}\hat{\sigma}_{21}^{z}-$
$32\hat{\sigma}_{2}^{z}\hat{\sigma}_{22}^{z}-4\hat{\sigma}_{2}^{z}\hat{\sigma}_{23}^{z}-4\hat{\sigma}_{2}^{z}\hat{\sigma}_{24}^{z}-4\hat{\sigma}_{2}^{z}\hat{\sigma}_{25}^{z}-32\hat{\sigma}_{2}^{z}\hat{\sigma}_{26}^{z}-4\hat{\sigma}_{2}^{z}\hat{\sigma}_{27}^{z}+6\hat{\sigma}_{2}^{z}\hat{\sigma}_{3}^{z}+2\hat{\sigma}_{2}^{z}\hat{\sigma}_{4}^{z}+2\hat{\sigma}_{2}^{z}\hat{\sigma}_{5}^{z}+16\hat{\sigma}_{2}^{z}\hat{\sigma}_{6}^{z}+2\hat{\sigma}_{2}^{z}\hat{\sigma}_{7}^{z}+$
$2\hat{\sigma}_{2}^{z}\hat{\sigma}_{8}^{z}+2\hat{\sigma}_{2}^{z}\hat{\sigma}_{9}^{z}+66\hat{\sigma}_{2}^{z}+8\hat{\sigma}_{20}^{z}\hat{\sigma}_{21}^{z}+8\hat{\sigma}_{20}^{z}\hat{\sigma}_{22}^{z}+8\hat{\sigma}_{20}^{z}\hat{\sigma}_{23}^{z}-4\hat{\sigma}_{20}^{z}\hat{\sigma}_{3}^{z}-32\hat{\sigma}_{20}^{z}\hat{\sigma}_{8}^{z}-4\hat{\sigma}_{20}^{z}\hat{\sigma}_{9}^{z}-44\hat{\sigma}_{20}^{z}+8\hat{\sigma}_{21}^{z}\hat{\sigma}_{22}^{z}+$
$8\hat{\sigma}_{21}^{z}\hat{\sigma}_{23}^{z}-4\hat{\sigma}_{21}^{z}\hat{\sigma}_{3}^{z}-4\hat{\sigma}_{21}^{z}\hat{\sigma}_{8}^{z}-32\hat{\sigma}_{21}^{z}\hat{\sigma}_{9}^{z}-44\hat{\sigma}_{21}^{z}+8\hat{\sigma}_{22}^{z}\hat{\sigma}_{23}^{z}-4\hat{\sigma}_{22}^{z}\hat{\sigma}_{3}^{z}-4\hat{\sigma}_{22}^{z}\hat{\sigma}_{8}^{z}-4\hat{\sigma}_{22}^{z}\hat{\sigma}_{9}^{z}-44\hat{\sigma}_{22}^{z}-32\hat{\sigma}_{23}^{z}\hat{\sigma}_{3}^{z}-$
$4\hat{\sigma}_{23}^{z}\hat{\sigma}_{8}^{z}-4\hat{\sigma}_{23}^{z}\hat{\sigma}_{9}^{z}-44\hat{\sigma}_{23}^{z}+8\hat{\sigma}_{24}^{z}\hat{\sigma}_{25}^{z}+8\hat{\sigma}_{24}^{z}\hat{\sigma}_{26}^{z}+8\hat{\sigma}_{24}^{z}\hat{\sigma}_{27}^{z}-4\hat{\sigma}_{24}^{z}\hat{\sigma}_{3}^{z}-44\hat{\sigma}_{24}^{z}+8\hat{\sigma}_{25}^{z}\hat{\sigma}_{26}^{z}+8\hat{\sigma}_{25}^{z}\hat{\sigma}_{27}^{z}-4\hat{\sigma}_{25}^{z}\hat{\sigma}_{3}^{z}-$
$44\hat{\sigma}_{25}^{z}+8\hat{\sigma}_{26}^{z}\hat{\sigma}_{27}^{z}-4\hat{\sigma}_{26}^{z}\hat{\sigma}_{3}^{z}-44\hat{\sigma}_{26}^{z}-32\hat{\sigma}_{27}^{z}\hat{\sigma}_{3}^{z}-44\hat{\sigma}_{27}^{z}+8\hat{\sigma}_{28}^{z}\hat{\sigma}_{29}^{z}+8\hat{\sigma}_{28}^{z}\hat{\sigma}_{30}^{z}+8\hat{\sigma}_{28}^{z}\hat{\sigma}_{31}^{z}-32\hat{\sigma}_{28}^{z}\hat{\sigma}_{4}^{z}-4\hat{\sigma}_{28}^{z}\hat{\sigma}_{5}^{z}-$
$4\hat{\sigma}_{28}^{z}\hat{\sigma}_{6}^{z}-4\hat{\sigma}_{28}^{z}\hat{\sigma}_{7}^{z}-32\hat{\sigma}_{28}^{z}\hat{\sigma}_{8}^{z}-4\hat{\sigma}_{28}^{z}\hat{\sigma}_{9}^{z}-44\hat{\sigma}_{28}^{z}+8\hat{\sigma}_{29}^{z}\hat{\sigma}_{30}^{z}+8\hat{\sigma}_{29}^{z}\hat{\sigma}_{31}^{z}-4\hat{\sigma}_{29}^{z}\hat{\sigma}_{4}^{z}-32\hat{\sigma}_{29}^{z}\hat{\sigma}_{5}^{z}-4\hat{\sigma}_{29}^{z}\hat{\sigma}_{6}^{z}-4\hat{\sigma}_{29}^{z}\hat{\sigma}_{7}^{z}-$
$4\hat{\sigma}_{29}^{z}\hat{\sigma}_{8}^{z}-32\hat{\sigma}_{29}^{z}\hat{\sigma}_{9}^{z}-44\hat{\sigma}_{29}^{z}+2\hat{\sigma}_{3}^{z}\hat{\sigma}_{4}^{z}+2\hat{\sigma}_{3}^{z}\hat{\sigma}_{5}^{z}+2\hat{\sigma}_{3}^{z}\hat{\sigma}_{6}^{z}+16\hat{\sigma}_{3}^{z}\hat{\sigma}_{7}^{z}+2\hat{\sigma}_{3}^{z}\hat{\sigma}_{8}^{z}+2\hat{\sigma}_{3}^{z}\hat{\sigma}_{9}^{z}+66\hat{\sigma}_{3}^{z}+8\hat{\sigma}_{30}^{z}\hat{\sigma}_{31}^{z}-$
$4\hat{\sigma}_{30}^{z}\hat{\sigma}_{4}^{z}-4\hat{\sigma}_{30}^{z}\hat{\sigma}_{5}^{z}-32\hat{\sigma}_{30}^{z}\hat{\sigma}_{6}^{z}-4\hat{\sigma}_{30}^{z}\hat{\sigma}_{7}^{z}-4\hat{\sigma}_{30}^{z}\hat{\sigma}_{8}^{z}-4\hat{\sigma}_{30}^{z}\hat{\sigma}_{9}^{z}-44\hat{\sigma}_{30}^{z}-4\hat{\sigma}_{31}^{z}\hat{\sigma}_{4}^{z}-4\hat{\sigma}_{31}^{z}\hat{\sigma}_{5}^{z}-4\hat{\sigma}_{31}^{z}\hat{\sigma}_{6}^{z}-32\hat{\sigma}_{31}^{z}\hat{\sigma}_{7}^{z}-$
$4\hat{\sigma}_{31}^{z}\hat{\sigma}_{8}^{z}-4\hat{\sigma}_{31}^{z}\hat{\sigma}_{9}^{z}-44\hat{\sigma}_{31}^{z}+8\hat{\sigma}_{32}^{z}\hat{\sigma}_{33}^{z}+8\hat{\sigma}_{32}^{z}\hat{\sigma}_{34}^{z}+8\hat{\sigma}_{32}^{z}\hat{\sigma}_{35}^{z}-32\hat{\sigma}_{32}^{z}\hat{\sigma}_{4}^{z}-4\hat{\sigma}_{32}^{z}\hat{\sigma}_{5}^{z}-4\hat{\sigma}_{32}^{z}\hat{\sigma}_{6}^{z}-4\hat{\sigma}_{32}^{z}\hat{\sigma}_{7}^{z}-44\hat{\sigma}_{32}^{z}+$
$8\hat{\sigma}_{33}^{z}\hat{\sigma}_{34}^{z}+8\hat{\sigma}_{33}^{z}\hat{\sigma}_{35}^{z}-4\hat{\sigma}_{33}^{z}\hat{\sigma}_{4}^{z}-32\hat{\sigma}_{33}^{z}\hat{\sigma}_{5}^{z}-4\hat{\sigma}_{33}^{z}\hat{\sigma}_{6}^{z}-4\hat{\sigma}_{33}^{z}\hat{\sigma}_{7}^{z}-44\hat{\sigma}_{33}^{z}+8\hat{\sigma}_{34}^{z}\hat{\sigma}_{35}^{z}-4\hat{\sigma}_{34}^{z}\hat{\sigma}_{4}^{z}-4\hat{\sigma}_{34}^{z}\hat{\sigma}_{5}^{z}-32\hat{\sigma}_{34}^{z}\hat{\sigma}_{6}^{z}-$
$4\hat{\sigma}_{34}^{z}\hat{\sigma}_{7}^{z}-44\hat{\sigma}_{34}^{z}-4\hat{\sigma}_{35}^{z}\hat{\sigma}_{4}^{z}-4\hat{\sigma}_{35}^{z}\hat{\sigma}_{5}^{z}-4\hat{\sigma}_{35}^{z}\hat{\sigma}_{6}^{z}-32\hat{\sigma}_{35}^{z}\hat{\sigma}_{7}^{z}-44\hat{\sigma}_{35}^{z}+8\hat{\sigma}_{36}^{z}\hat{\sigma}_{37}^{z}+8\hat{\sigma}_{36}^{z}\hat{\sigma}_{38}^{z}+8\hat{\sigma}_{36}^{z}\hat{\sigma}_{39}^{z}-32\hat{\sigma}_{36}^{z}\hat{\sigma}_{8}^{z}-$
$4\hat{\sigma}_{36}^{z}\hat{\sigma}_{9}^{z}-44\hat{\sigma}_{36}^{z}+8\hat{\sigma}_{37}^{z}\hat{\sigma}_{38}^{z}+8\hat{\sigma}_{37}^{z}\hat{\sigma}_{39}^{z}-4\hat{\sigma}_{37}^{z}\hat{\sigma}_{8}^{z}-32\hat{\sigma}_{37}^{z}\hat{\sigma}_{9}^{z}-44\hat{\sigma}_{37}^{z}+8\hat{\sigma}_{38}^{z}\hat{\sigma}_{39}^{z}-4\hat{\sigma}_{38}^{z}\hat{\sigma}_{8}^{z}-4\hat{\sigma}_{38}^{z}\hat{\sigma}_{9}^{z}-44\hat{\sigma}_{38}^{z}-$
$4\hat{\sigma}_{39}^{z}\hat{\sigma}_{8}^{z}-4\hat{\sigma}_{39}^{z}\hat{\sigma}_{9}^{z}-44\hat{\sigma}_{39}^{z}+6\hat{\sigma}_{4}^{z}\hat{\sigma}_{5}^{z}+6\hat{\sigma}_{4}^{z}\hat{\sigma}_{6}^{z}+6\hat{\sigma}_{4}^{z}\hat{\sigma}_{7}^{z}+16\hat{\sigma}_{4}^{z}\hat{\sigma}_{8}^{z}+2\hat{\sigma}_{4}^{z}\hat{\sigma}_{9}^{z}+66\hat{\sigma}_{4}^{z}+6\hat{\sigma}_{5}^{z}\hat{\sigma}_{6}^{z}+6\hat{\sigma}_{5}^{z}\hat{\sigma}_{7}^{z}+2\hat{\sigma}_{5}^{z}\hat{\sigma}_{8}^{z}+$
$16\hat{\sigma}_{5}^{z}\hat{\sigma}_{9}^{z}+66\hat{\sigma}_{5}^{z}+6\hat{\sigma}_{6}^{z}\hat{\sigma}_{7}^{z}+2\hat{\sigma}_{6}^{z}\hat{\sigma}_{8}^{z}+2\hat{\sigma}_{6}^{z}\hat{\sigma}_{9}^{z}+66\hat{\sigma}_{6}^{z}+2\hat{\sigma}_{7}^{z}\hat{\sigma}_{8}^{z}+2\hat{\sigma}_{7}^{z}\hat{\sigma}_{9}^{z}+66\hat{\sigma}_{7}^{z}+6\hat{\sigma}_{8}^{z}\hat{\sigma}_{9}^{z}+66\hat{\sigma}_{8}^{z}+66\hat{\sigma}_{9}^{z}+1,248$

A complete expression of the Eq.(13)

$\hat{H}_2\left(\hat{\sigma}\right)=4\hat{\sigma}_{0}^{z}\hat{\sigma}_{1}^{z}+2\hat{\sigma}_{0}^{z}\hat{\sigma}_{10}^{z}+2\hat{\sigma}_{0}^{z}\hat{\sigma}_{11}^{z}-40\hat{\sigma}_{0}^{z}\hat{\sigma}_{12}^{z}-4\hat{\sigma}_{0}^{z}\hat{\sigma}_{13}^{z}-4\hat{\sigma}_{0}^{z}\hat{\sigma}_{14}^{z}-4\hat{\sigma}_{0}^{z}\hat{\sigma}_{15}^{z}-40\hat{\sigma}_{0}^{z}\hat{\sigma}_{16}^{z}-4\hat{\sigma}_{0}^{z}\hat{\sigma}_{17}^{z}-$
$4\hat{\sigma}_{0}^{z}\hat{\sigma}_{18}^{z}-4\hat{\sigma}_{0}^{z}\hat{\sigma}_{19}^{z}+4\hat{\sigma}_{0}^{z}\hat{\sigma}_{2}^{z}+4\hat{\sigma}_{0}^{z}\hat{\sigma}_{3}^{z}+20\hat{\sigma}_{0}^{z}\hat{\sigma}_{4}^{z}+2\hat{\sigma}_{0}^{z}\hat{\sigma}_{5}^{z}+2\hat{\sigma}_{0}^{z}\hat{\sigma}_{6}^{z}+2\hat{\sigma}_{0}^{z}\hat{\sigma}_{7}^{z}+20\hat{\sigma}_{0}^{z}\hat{\sigma}_{8}^{z}+2\hat{\sigma}_{0}^{z}\hat{\sigma}_{9}^{z}+52\hat{\sigma}_{0}^{z}+$
$2\hat{\sigma}_{1}^{z}\hat{\sigma}_{10}^{z}+2\hat{\sigma}_{1}^{z}\hat{\sigma}_{11}^{z}-4\hat{\sigma}_{1}^{z}\hat{\sigma}_{12}^{z}-40\hat{\sigma}_{1}^{z}\hat{\sigma}_{13}^{z}-4\hat{\sigma}_{1}^{z}\hat{\sigma}_{14}^{z}-4\hat{\sigma}_{1}^{z}\hat{\sigma}_{15}^{z}-4\hat{\sigma}_{1}^{z}\hat{\sigma}_{16}^{z}-40\hat{\sigma}_{1}^{z}\hat{\sigma}_{17}^{z}-4\hat{\sigma}_{1}^{z}\hat{\sigma}_{18}^{z}-4\hat{\sigma}_{1}^{z}\hat{\sigma}_{19}^{z}+4\hat{\sigma}_{1}^{z}\hat{\sigma}_{2}^{z}+$
$4\hat{\sigma}_{1}^{z}\hat{\sigma}_{3}^{z}+2\hat{\sigma}_{1}^{z}\hat{\sigma}_{4}^{z}+20\hat{\sigma}_{1}^{z}\hat{\sigma}_{5}^{z}+2\hat{\sigma}_{1}^{z}\hat{\sigma}_{6}^{z}+2\hat{\sigma}_{1}^{z}\hat{\sigma}_{7}^{z}+2\hat{\sigma}_{1}^{z}\hat{\sigma}_{8}^{z}+20\hat{\sigma}_{1}^{z}\hat{\sigma}_{9}^{z}+52\hat{\sigma}_{1}^{z}+4\hat{\sigma}_{10}^{z}\hat{\sigma}_{11}^{z}-4\hat{\sigma}_{10}^{z}\hat{\sigma}_{16}^{z}-4\hat{\sigma}_{10}^{z}\hat{\sigma}_{17}^{z}-$
$40\hat{\sigma}_{10}^{z}\hat{\sigma}_{18}^{z}-4\hat{\sigma}_{10}^{z}\hat{\sigma}_{19}^{z}+20\hat{\sigma}_{10}^{z}\hat{\sigma}_{2}^{z}-4\hat{\sigma}_{10}^{z}\hat{\sigma}_{20}^{z}-4\hat{\sigma}_{10}^{z}\hat{\sigma}_{21}^{z}-40\hat{\sigma}_{10}^{z}\hat{\sigma}_{22}^{z}-4\hat{\sigma}_{10}^{z}\hat{\sigma}_{23}^{z}+2\hat{\sigma}_{10}^{z}\hat{\sigma}_{3}^{z}+2\hat{\sigma}_{10}^{z}\hat{\sigma}_{4}^{z}+2\hat{\sigma}_{10}^{z}\hat{\sigma}_{5}^{z}+$
$20\hat{\sigma}_{10}^{z}\hat{\sigma}_{6}^{z}+2\hat{\sigma}_{10}^{z}\hat{\sigma}_{7}^{z}+4\hat{\sigma}_{10}^{z}\hat{\sigma}_{8}^{z}+4\hat{\sigma}_{10}^{z}\hat{\sigma}_{9}^{z}+52\hat{\sigma}_{10}^{z}-4\hat{\sigma}_{11}^{z}\hat{\sigma}_{16}^{z}-4\hat{\sigma}_{11}^{z}\hat{\sigma}_{17}^{z}-4\hat{\sigma}_{11}^{z}\hat{\sigma}_{18}^{z}-40\hat{\sigma}_{11}^{z}\hat{\sigma}_{19}^{z}+2\hat{\sigma}_{11}^{z}\hat{\sigma}_{2}^{z}-$
$4\hat{\sigma}_{11}^{z}\hat{\sigma}_{20}^{z}-4\hat{\sigma}_{11}^{z}\hat{\sigma}_{21}^{z}-4\hat{\sigma}_{11}^{z}\hat{\sigma}_{22}^{z}-40\hat{\sigma}_{11}^{z}\hat{\sigma}_{23}^{z}+20\hat{\sigma}_{11}^{z}\hat{\sigma}_{3}^{z}+2\hat{\sigma}_{11}^{z}\hat{\sigma}_{4}^{z}+2\hat{\sigma}_{11}^{z}\hat{\sigma}_{5}^{z}+2\hat{\sigma}_{11}^{z}\hat{\sigma}_{6}^{z}+20\hat{\sigma}_{11}^{z}\hat{\sigma}_{7}^{z}+4\hat{\sigma}_{11}^{z}\hat{\sigma}_{8}^{z}+$
$4\hat{\sigma}_{11}^{z}\hat{\sigma}_{9}^{z}+52\hat{\sigma}_{11}^{z}+8\hat{\sigma}_{12}^{z}\hat{\sigma}_{13}^{z}+8\hat{\sigma}_{12}^{z}\hat{\sigma}_{14}^{z}+8\hat{\sigma}_{12}^{z}\hat{\sigma}_{15}^{z}-4\hat{\sigma}_{12}^{z}\hat{\sigma}_{2}^{z}-4\hat{\sigma}_{12}^{z}\hat{\sigma}_{3}^{z}-40\hat{\sigma}_{12}^{z}\hat{\sigma}_{4}^{z}-4\hat{\sigma}_{12}^{z}\hat{\sigma}_{5}^{z}-4\hat{\sigma}_{12}^{z}\hat{\sigma}_{6}^{z}-4\hat{\sigma}_{12}^{z}\hat{\sigma}_{7}^{z}-$
$52\hat{\sigma}_{12}^{z}+8\hat{\sigma}_{13}^{z}\hat{\sigma}_{14}^{z}+8\hat{\sigma}_{13}^{z}\hat{\sigma}_{15}^{z}-4\hat{\sigma}_{13}^{z}\hat{\sigma}_{2}^{z}-4\hat{\sigma}_{13}^{z}\hat{\sigma}_{3}^{z}-4\hat{\sigma}_{13}^{z}\hat{\sigma}_{4}^{z}-40\hat{\sigma}_{13}^{z}\hat{\sigma}_{5}^{z}-4\hat{\sigma}_{13}^{z}\hat{\sigma}_{6}^{z}-4\hat{\sigma}_{13}^{z}\hat{\sigma}_{7}^{z}-52\hat{\sigma}_{13}^{z}+8\hat{\sigma}_{14}^{z}\hat{\sigma}_{15}^{z}-$
$40\hat{\sigma}_{14}^{z}\hat{\sigma}_{2}^{z}-4\hat{\sigma}_{14}^{z}\hat{\sigma}_{3}^{z}-4\hat{\sigma}_{14}^{z}\hat{\sigma}_{4}^{z}-4\hat{\sigma}_{14}^{z}\hat{\sigma}_{5}^{z}-40\hat{\sigma}_{14}^{z}\hat{\sigma}_{6}^{z}-4\hat{\sigma}_{14}^{z}\hat{\sigma}_{7}^{z}-52\hat{\sigma}_{14}^{z}-4\hat{\sigma}_{15}^{z}\hat{\sigma}_{2}^{z}-40\hat{\sigma}_{15}^{z}\hat{\sigma}_{3}^{z}-4\hat{\sigma}_{15}^{z}\hat{\sigma}_{4}^{z}-$
$4\hat{\sigma}_{15}^{z}\hat{\sigma}_{5}^{z}-4\hat{\sigma}_{15}^{z}\hat{\sigma}_{6}^{z}-40\hat{\sigma}_{15}^{z}\hat{\sigma}_{7}^{z}-52\hat{\sigma}_{15}^{z}+8\hat{\sigma}_{16}^{z}\hat{\sigma}_{17}^{z}+8\hat{\sigma}_{16}^{z}\hat{\sigma}_{18}^{z}+8\hat{\sigma}_{16}^{z}\hat{\sigma}_{19}^{z}-4\hat{\sigma}_{16}^{z}\hat{\sigma}_{2}^{z}-4\hat{\sigma}_{16}^{z}\hat{\sigma}_{3}^{z}-40\hat{\sigma}_{16}^{z}\hat{\sigma}_{8}^{z}-$
$4\hat{\sigma}_{16}^{z}\hat{\sigma}_{9}^{z}-52\hat{\sigma}_{16}^{z}+8\hat{\sigma}_{17}^{z}\hat{\sigma}_{18}^{z}+8\hat{\sigma}_{17}^{z}\hat{\sigma}_{19}^{z}-4\hat{\sigma}_{17}^{z}\hat{\sigma}_{2}^{z}-4\hat{\sigma}_{17}^{z}\hat{\sigma}_{3}^{z}-4\hat{\sigma}_{17}^{z}\hat{\sigma}_{8}^{z}-40\hat{\sigma}_{17}^{z}\hat{\sigma}_{9}^{z}-52\hat{\sigma}_{17}^{z}+8\hat{\sigma}_{18}^{z}\hat{\sigma}_{19}^{z}-40\hat{\sigma}_{18}^{z}\hat{\sigma}_{2}^{z}-$
$4\hat{\sigma}_{18}^{z}\hat{\sigma}_{3}^{z}-4\hat{\sigma}_{18}^{z}\hat{\sigma}_{8}^{z}-4\hat{\sigma}_{18}^{z}\hat{\sigma}_{9}^{z}-52\hat{\sigma}_{18}^{z}-4\hat{\sigma}_{19}^{z}\hat{\sigma}_{2}^{z}-40\hat{\sigma}_{19}^{z}\hat{\sigma}_{3}^{z}-4\hat{\sigma}_{19}^{z}\hat{\sigma}_{8}^{z}-4\hat{\sigma}_{19}^{z}\hat{\sigma}_{9}^{z}-52\hat{\sigma}_{19}^{z}+4\hat{\sigma}_{2}^{z}\hat{\sigma}_{3}^{z}+2\hat{\sigma}_{2}^{z}\hat{\sigma}_{4}^{z}+$
$2\hat{\sigma}_{2}^{z}\hat{\sigma}_{5}^{z}+20\hat{\sigma}_{2}^{z}\hat{\sigma}_{6}^{z}+2\hat{\sigma}_{2}^{z}\hat{\sigma}_{7}^{z}+2\hat{\sigma}_{2}^{z}\hat{\sigma}_{8}^{z}+2\hat{\sigma}_{2}^{z}\hat{\sigma}_{9}^{z}+52\hat{\sigma}_{2}^{z}+8\hat{\sigma}_{20}^{z}\hat{\sigma}_{21}^{z}+8\hat{\sigma}_{20}^{z}\hat{\sigma}_{22}^{z}+8\hat{\sigma}_{20}^{z}\hat{\sigma}_{23}^{z}-40\hat{\sigma}_{20}^{z}\hat{\sigma}_{4}^{z}-4\hat{\sigma}_{20}^{z}\hat{\sigma}_{5}^{z}-$
$4\hat{\sigma}_{20}^{z}\hat{\sigma}_{6}^{z}-4\hat{\sigma}_{20}^{z}\hat{\sigma}_{7}^{z}-40\hat{\sigma}_{20}^{z}\hat{\sigma}_{8}^{z}-4\hat{\sigma}_{20}^{z}\hat{\sigma}_{9}^{z}-52\hat{\sigma}_{20}^{z}+8\hat{\sigma}_{21}^{z}\hat{\sigma}_{22}^{z}+8\hat{\sigma}_{21}^{z}\hat{\sigma}_{23}^{z}-4\hat{\sigma}_{21}^{z}\hat{\sigma}_{4}^{z}-40\hat{\sigma}_{21}^{z}\hat{\sigma}_{5}^{z}-4\hat{\sigma}_{21}^{z}\hat{\sigma}_{6}^{z}-4\hat{\sigma}_{21}^{z}\hat{\sigma}_{7}^{z}-$
$4\hat{\sigma}_{21}^{z}\hat{\sigma}_{8}^{z}-40\hat{\sigma}_{21}^{z}\hat{\sigma}_{9}^{z}-52\hat{\sigma}_{21}^{z}+8\hat{\sigma}_{22}^{z}\hat{\sigma}_{23}^{z}-4\hat{\sigma}_{22}^{z}\hat{\sigma}_{4}^{z}-4\hat{\sigma}_{22}^{z}\hat{\sigma}_{5}^{z}-40\hat{\sigma}_{22}^{z}\hat{\sigma}_{6}^{z}-4\hat{\sigma}_{22}^{z}\hat{\sigma}_{7}^{z}-4\hat{\sigma}_{22}^{z}\hat{\sigma}_{8}^{z}-4\hat{\sigma}_{22}^{z}\hat{\sigma}_{9}^{z}-52\hat{\sigma}_{22}^{z}-$
$4\hat{\sigma}_{23}^{z}\hat{\sigma}_{4}^{z}-4\hat{\sigma}_{23}^{z}\hat{\sigma}_{5}^{z}-4\hat{\sigma}_{23}^{z}\hat{\sigma}_{6}^{z}-40\hat{\sigma}_{23}^{z}\hat{\sigma}_{7}^{z}-4\hat{\sigma}_{23}^{z}\hat{\sigma}_{8}^{z}-4\hat{\sigma}_{23}^{z}\hat{\sigma}_{9}^{z}-52\hat{\sigma}_{23}^{z}+2\hat{\sigma}_{3}^{z}\hat{\sigma}_{4}^{z}+2\hat{\sigma}_{3}^{z}\hat{\sigma}_{5}^{z}+2\hat{\sigma}_{3}^{z}\hat{\sigma}_{6}^{z}+20\hat{\sigma}_{3}^{z}\hat{\sigma}_{7}^{z}+$
$2\hat{\sigma}_{3}^{z}\hat{\sigma}_{8}^{z}+2\hat{\sigma}_{3}^{z}\hat{\sigma}_{9}^{z}+52\hat{\sigma}_{3}^{z}+4\hat{\sigma}_{4}^{z}\hat{\sigma}_{5}^{z}+4\hat{\sigma}_{4}^{z}\hat{\sigma}_{6}^{z}+4\hat{\sigma}_{4}^{z}\hat{\sigma}_{7}^{z}+20\hat{\sigma}_{4}^{z}\hat{\sigma}_{8}^{z}+2\hat{\sigma}_{4}^{z}\hat{\sigma}_{9}^{z}+52\hat{\sigma}_{4}^{z}+4\hat{\sigma}_{5}^{z}\hat{\sigma}_{6}^{z}+4\hat{\sigma}_{5}^{z}\hat{\sigma}_{7}^{z}+2\hat{\sigma}_{5}^{z}\hat{\sigma}_{8}^{z}+$
$20\hat{\sigma}_{5}^{z}\hat{\sigma}_{9}^{z}+52\hat{\sigma}_{5}^{z}+4\hat{\sigma}_{6}^{z}\hat{\sigma}_{7}^{z}+2\hat{\sigma}_{6}^{z}\hat{\sigma}_{8}^{z}+2\hat{\sigma}_{6}^{z}\hat{\sigma}_{9}^{z}+52\hat{\sigma}_{6}^{z}+2\hat{\sigma}_{7}^{z}\hat{\sigma}_{8}^{z}+2\hat{\sigma}_{7}^{z}\hat{\sigma}_{9}^{z}+52\hat{\sigma}_{7}^{z}+4\hat{\sigma}_{8}^{z}\hat{\sigma}_{9}^{z}+52\hat{\sigma}_{8}^{z}+52\hat{\sigma}_{9}^{z}+768$

A complete expression of Eq.(14)

$\hat{H}_2\left(\hat{\sigma}\right)=2\hat{\sigma}_{0}^{z}\hat{\sigma}_{1}^{z}-4\hat{\sigma}_{0}^{z}\hat{\sigma}_{10}^{z}-4\hat{\sigma}_{0}^{z}\hat{\sigma}_{11}^{z}+6\hat{\sigma}_{0}^{z}\hat{\sigma}_{2}^{z}+2\hat{\sigma}_{0}^{z}\hat{\sigma}_{3}^{z}+8\hat{\sigma}_{0}^{z}\hat{\sigma}_{4}^{z}+2\hat{\sigma}_{0}^{z}\hat{\sigma}_{5}^{z}+2\hat{\sigma}_{0}^{z}\hat{\sigma}_{6}^{z}+2\hat{\sigma}_{0}^{z}\hat{\sigma}_{7}^{z}-16\hat{\sigma}_{0}^{z}\hat{\sigma}_{8}^{z}-$
$4\hat{\sigma}_{0}^{z}\hat{\sigma}_{9}^{z}+14\hat{\sigma}_{0}^{z}-4\hat{\sigma}_{1}^{z}\hat{\sigma}_{10}^{z}-4\hat{\sigma}_{1}^{z}\hat{\sigma}_{11}^{z}+2\hat{\sigma}_{1}^{z}\hat{\sigma}_{2}^{z}+6\hat{\sigma}_{1}^{z}\hat{\sigma}_{3}^{z}+2\hat{\sigma}_{1}^{z}\hat{\sigma}_{4}^{z}+8\hat{\sigma}_{1}^{z}\hat{\sigma}_{5}^{z}+2\hat{\sigma}_{1}^{z}\hat{\sigma}_{6}^{z}+2\hat{\sigma}_{1}^{z}\hat{\sigma}_{7}^{z}-4\hat{\sigma}_{1}^{z}\hat{\sigma}_{8}^{z}-$
$16\hat{\sigma}_{1}^{z}\hat{\sigma}_{9}^{z}+14\hat{\sigma}_{1}^{z}+8\hat{\sigma}_{10}^{z}\hat{\sigma}_{11}^{z}-16\hat{\sigma}_{10}^{z}\hat{\sigma}_{2}^{z}-4\hat{\sigma}_{10}^{z}\hat{\sigma}_{3}^{z}-4\hat{\sigma}_{10}^{z}\hat{\sigma}_{4}^{z}-4\hat{\sigma}_{10}^{z}\hat{\sigma}_{5}^{z}-16\hat{\sigma}_{10}^{z}\hat{\sigma}_{6}^{z}-4\hat{\sigma}_{10}^{z}\hat{\sigma}_{7}^{z}+8\hat{\sigma}_{10}^{z}\hat{\sigma}_{8}^{z}+8\hat{\sigma}_{10}^{z}\hat{\sigma}_{9}^{z}-$
$28\hat{\sigma}_{10}^{z}-4\hat{\sigma}_{11}^{z}\hat{\sigma}_{2}^{z}-16\hat{\sigma}_{11}^{z}\hat{\sigma}_{3}^{z}-4\hat{\sigma}_{11}^{z}\hat{\sigma}_{4}^{z}-4\hat{\sigma}_{11}^{z}\hat{\sigma}_{5}^{z}-4\hat{\sigma}_{11}^{z}\hat{\sigma}_{6}^{z}-16\hat{\sigma}_{11}^{z}\hat{\sigma}_{7}^{z}+8\hat{\sigma}_{11}^{z}\hat{\sigma}_{8}^{z}+8\hat{\sigma}_{11}^{z}\hat{\sigma}_{9}^{z}-28\hat{\sigma}_{11}^{z}+2\hat{\sigma}_{2}^{z}\hat{\sigma}_{3}^{z}+$
$2\hat{\sigma}_{2}^{z}\hat{\sigma}_{4}^{z}+2\hat{\sigma}_{2}^{z}\hat{\sigma}_{5}^{z}+8\hat{\sigma}_{2}^{z}\hat{\sigma}_{6}^{z}+2\hat{\sigma}_{2}^{z}\hat{\sigma}_{7}^{z}-4\hat{\sigma}_{2}^{z}\hat{\sigma}_{8}^{z}-4\hat{\sigma}_{2}^{z}\hat{\sigma}_{9}^{z}+14\hat{\sigma}_{2}^{z}+2\hat{\sigma}_{3}^{z}\hat{\sigma}_{4}^{z}+2\hat{\sigma}_{3}^{z}\hat{\sigma}_{5}^{z}+2\hat{\sigma}_{3}^{z}\hat{\sigma}_{6}^{z}+8\hat{\sigma}_{3}^{z}\hat{\sigma}_{7}^{z}-$
$4\hat{\sigma}_{3}^{z}\hat{\sigma}_{8}^{z}-4\hat{\sigma}_{3}^{z}\hat{\sigma}_{9}^{z}+14\hat{\sigma}_{3}^{z}+2\hat{\sigma}_{4}^{z}\hat{\sigma}_{5}^{z}+6\hat{\sigma}_{4}^{z}\hat{\sigma}_{6}^{z}+2\hat{\sigma}_{4}^{z}\hat{\sigma}_{7}^{z}-16\hat{\sigma}_{4}^{z}\hat{\sigma}_{8}^{z}-4\hat{\sigma}_{4}^{z}\hat{\sigma}_{9}^{z}+14\hat{\sigma}_{4}^{z}+2\hat{\sigma}_{5}^{z}\hat{\sigma}_{6}^{z}+6\hat{\sigma}_{5}^{z}\hat{\sigma}_{7}^{z}-4\hat{\sigma}_{5}^{z}\hat{\sigma}_{8}^{z}-$
$16\hat{\sigma}_{5}^{z}\hat{\sigma}_{9}^{z}+14\hat{\sigma}_{5}^{z}+2\hat{\sigma}_{6}^{z}\hat{\sigma}_{7}^{z}-4\hat{\sigma}_{6}^{z}\hat{\sigma}_{8}^{z}-4\hat{\sigma}_{6}^{z}\hat{\sigma}_{9}^{z}+14\hat{\sigma}_{6}^{z}-4\hat{\sigma}_{7}^{z}\hat{\sigma}_{8}^{z}-4\hat{\sigma}_{7}^{z}\hat{\sigma}_{9}^{z}+14\hat{\sigma}_{7}^{z}+8\hat{\sigma}_{8}^{z}\hat{\sigma}_{9}^{z}-28\hat{\sigma}_{8}^{z}-28\hat{\sigma}_{9}^{z}+128$

A complete expression of Eq.(17)

$$\hat{H}_2(\hat{\sigma}) = 4\hat{\sigma}_{0}^{z}\hat{\sigma}_{1}^{z} + 4\hat{\sigma}_{0}^{z}\hat{\sigma}_{10}^{z} + 4\hat{\sigma}_{0}^{z}\hat{\sigma}_{11}^{z} + 180\hat{\sigma}_{0}^{z}\hat{\sigma}_{12}^{z} + 2\hat{\sigma}_{0}^{z}\hat{\sigma}_{13}^{z} + 2\hat{\sigma}_{0}^{z}\hat{\sigma}_{14}^{z} + 2\hat{\sigma}_{0}^{z}\hat{\sigma}_{15}^{z} + 2\hat{\sigma}_{0}^{z}\hat{\sigma}_{16}^{z} + 2\hat{\sigma}_{0}^{z}\hat{\sigma}_{17}^{z} + 2\hat{\sigma}_{0}^{z}\hat{\sigma}_{18}^{z} +$$
$$2\hat{\sigma}_{0}^{z}\hat{\sigma}_{19}^{z} + 4\hat{\sigma}_{0}^{z}\hat{\sigma}_{2}^{z} + 2\hat{\sigma}_{0}^{z}\hat{\sigma}_{20}^{z} + 2\hat{\sigma}_{0}^{z}\hat{\sigma}_{21}^{z} + 2\hat{\sigma}_{0}^{z}\hat{\sigma}_{22}^{z} + 2\hat{\sigma}_{0}^{z}\hat{\sigma}_{23}^{z} + 180\hat{\sigma}_{0}^{z}\hat{\sigma}_{24}^{z} + 2\hat{\sigma}_{0}^{z}\hat{\sigma}_{25}^{z} + 2\hat{\sigma}_{0}^{z}\hat{\sigma}_{26}^{z} + 2\hat{\sigma}_{0}^{z}\hat{\sigma}_{27}^{z} + 2\hat{\sigma}_{0}^{z}\hat{\sigma}_{28}^{z} +$$
$$2\hat{\sigma}_{0}^{z}\hat{\sigma}_{29}^{z} + 4\hat{\sigma}_{0}^{z}\hat{\sigma}_{3}^{z} + 2\hat{\sigma}_{0}^{z}\hat{\sigma}_{30}^{z} + 2\hat{\sigma}_{0}^{z}\hat{\sigma}_{31}^{z} + 2\hat{\sigma}_{0}^{z}\hat{\sigma}_{32}^{z} + 2\hat{\sigma}_{0}^{z}\hat{\sigma}_{33}^{z} + 2\hat{\sigma}_{0}^{z}\hat{\sigma}_{34}^{z} + 2\hat{\sigma}_{0}^{z}\hat{\sigma}_{35}^{z} - 360\hat{\sigma}_{0}^{z}\hat{\sigma}_{36}^{z} - 4\hat{\sigma}_{0}^{z}\hat{\sigma}_{37}^{z} - 4\hat{\sigma}_{0}^{z}\hat{\sigma}_{38}^{z} -$$
$$4\hat{\sigma}_{0}^{z}\hat{\sigma}_{39}^{z} + 4\hat{\sigma}_{0}^{z}\hat{\sigma}_{4}^{z} - 4\hat{\sigma}_{0}^{z}\hat{\sigma}_{40}^{z} - 4\hat{\sigma}_{0}^{z}\hat{\sigma}_{41}^{z} - 4\hat{\sigma}_{0}^{z}\hat{\sigma}_{42}^{z} - 4\hat{\sigma}_{0}^{z}\hat{\sigma}_{43}^{z} - 4\hat{\sigma}_{0}^{z}\hat{\sigma}_{44}^{z} - 4\hat{\sigma}_{0}^{z}\hat{\sigma}_{45}^{z} - 4\hat{\sigma}_{0}^{z}\hat{\sigma}_{46}^{z} - 4\hat{\sigma}_{0}^{z}\hat{\sigma}_{47}^{z} - 360\hat{\sigma}_{0}^{z}\hat{\sigma}_{48}^{z} -$$
$$4\hat{\sigma}_{0}^{z}\hat{\sigma}_{49}^{z} + 4\hat{\sigma}_{0}^{z}\hat{\sigma}_{5}^{z} - 4\hat{\sigma}_{0}^{z}\hat{\sigma}_{50}^{z} - 4\hat{\sigma}_{0}^{z}\hat{\sigma}_{51}^{z} - 4\hat{\sigma}_{0}^{z}\hat{\sigma}_{52}^{z} - 4\hat{\sigma}_{0}^{z}\hat{\sigma}_{53}^{z} - 4\hat{\sigma}_{0}^{z}\hat{\sigma}_{54}^{z} - 4\hat{\sigma}_{0}^{z}\hat{\sigma}_{55}^{z} - 4\hat{\sigma}_{0}^{z}\hat{\sigma}_{56}^{z} - 4\hat{\sigma}_{0}^{z}\hat{\sigma}_{57}^{z} - 4\hat{\sigma}_{0}^{z}\hat{\sigma}_{58}^{z} -$$
$$4\hat{\sigma}_{0}^{z}\hat{\sigma}_{59}^{z} + 4\hat{\sigma}_{0}^{z}\hat{\sigma}_{6}^{z} + 4\hat{\sigma}_{0}^{z}\hat{\sigma}_{7}^{z} + 4\hat{\sigma}_{0}^{z}\hat{\sigma}_{8}^{z} + 4\hat{\sigma}_{0}^{z}\hat{\sigma}_{9}^{z} + 404\hat{\sigma}_{0}^{z} + 4\hat{\sigma}_{1}^{z}\hat{\sigma}_{10}^{z} + 4\hat{\sigma}_{1}^{z}\hat{\sigma}_{11}^{z} + 2\hat{\sigma}_{1}^{z}\hat{\sigma}_{12}^{z} + 180\hat{\sigma}_{1}^{z}\hat{\sigma}_{13}^{z} + 2\hat{\sigma}_{1}^{z}\hat{\sigma}_{14}^{z} +$$
$$2\hat{\sigma}_{1}^{z}\hat{\sigma}_{15}^{z} + 2\hat{\sigma}_{1}^{z}\hat{\sigma}_{16}^{z} + 2\hat{\sigma}_{1}^{z}\hat{\sigma}_{17}^{z} + 2\hat{\sigma}_{1}^{z}\hat{\sigma}_{18}^{z} + 2\hat{\sigma}_{1}^{z}\hat{\sigma}_{19}^{z} + 4\hat{\sigma}_{1}^{z}\hat{\sigma}_{2}^{z} + 2\hat{\sigma}_{1}^{z}\hat{\sigma}_{20}^{z} + 2\hat{\sigma}_{1}^{z}\hat{\sigma}_{21}^{z} + 2\hat{\sigma}_{1}^{z}\hat{\sigma}_{22}^{z} + 2\hat{\sigma}_{1}^{z}\hat{\sigma}_{23}^{z} + 2\hat{\sigma}_{1}^{z}\hat{\sigma}_{24}^{z} +$$
$$180\hat{\sigma}_{1}^{z}\hat{\sigma}_{25}^{z} + 2\hat{\sigma}_{1}^{z}\hat{\sigma}_{26}^{z} + 2\hat{\sigma}_{1}^{z}\hat{\sigma}_{27}^{z} + 2\hat{\sigma}_{1}^{z}\hat{\sigma}_{28}^{z} + 2\hat{\sigma}_{1}^{z}\hat{\sigma}_{29}^{z} + 4\hat{\sigma}_{1}^{z}\hat{\sigma}_{3}^{z} + 2\hat{\sigma}_{1}^{z}\hat{\sigma}_{30}^{z} + 2\hat{\sigma}_{1}^{z}\hat{\sigma}_{31}^{z} + 2\hat{\sigma}_{1}^{z}\hat{\sigma}_{32}^{z} + 2\hat{\sigma}_{1}^{z}\hat{\sigma}_{33}^{z} + 2\hat{\sigma}_{1}^{z}\hat{\sigma}_{34}^{z} +$$
$$2\hat{\sigma}_{1}^{z}\hat{\sigma}_{35}^{z} - 4\hat{\sigma}_{1}^{z}\hat{\sigma}_{36}^{z} - 360\hat{\sigma}_{1}^{z}\hat{\sigma}_{37}^{z} - 4\hat{\sigma}_{1}^{z}\hat{\sigma}_{38}^{z} - 4\hat{\sigma}_{1}^{z}\hat{\sigma}_{39}^{z} + 4\hat{\sigma}_{1}^{z}\hat{\sigma}_{4}^{z} - 4\hat{\sigma}_{1}^{z}\hat{\sigma}_{40}^{z} - 4\hat{\sigma}_{1}^{z}\hat{\sigma}_{41}^{z} - 4\hat{\sigma}_{1}^{z}\hat{\sigma}_{42}^{z} - 4\hat{\sigma}_{1}^{z}\hat{\sigma}_{43}^{z} - 4\hat{\sigma}_{1}^{z}\hat{\sigma}_{44}^{z} -$$
$$4\hat{\sigma}_{1}^{z}\hat{\sigma}_{45}^{z} - 4\hat{\sigma}_{1}^{z}\hat{\sigma}_{46}^{z} - 4\hat{\sigma}_{1}^{z}\hat{\sigma}_{47}^{z} - 4\hat{\sigma}_{1}^{z}\hat{\sigma}_{48}^{z} - 360\hat{\sigma}_{1}^{z}\hat{\sigma}_{49}^{z} + 4\hat{\sigma}_{1}^{z}\hat{\sigma}_{5}^{z} - 4\hat{\sigma}_{1}^{z}\hat{\sigma}_{50}^{z} - 4\hat{\sigma}_{1}^{z}\hat{\sigma}_{51}^{z} - 4\hat{\sigma}_{1}^{z}\hat{\sigma}_{52}^{z} - 4\hat{\sigma}_{1}^{z}\hat{\sigma}_{53}^{z} - 4\hat{\sigma}_{1}^{z}\hat{\sigma}_{54}^{z} -$$
$$4\hat{\sigma}_{1}^{z}\hat{\sigma}_{55}^{z} - 4\hat{\sigma}_{1}^{z}\hat{\sigma}_{56}^{z} - 4\hat{\sigma}_{1}^{z}\hat{\sigma}_{57}^{z} - 4\hat{\sigma}_{1}^{z}\hat{\sigma}_{58}^{z} - 4\hat{\sigma}_{1}^{z}\hat{\sigma}_{59}^{z} + 4\hat{\sigma}_{1}^{z}\hat{\sigma}_{6}^{z} + 4\hat{\sigma}_{1}^{z}\hat{\sigma}_{7}^{z} + 4\hat{\sigma}_{1}^{z}\hat{\sigma}_{8}^{z} + 4\hat{\sigma}_{1}^{z}\hat{\sigma}_{9}^{z} + 404\hat{\sigma}_{1}^{z} + 4\hat{\sigma}_{10}^{z}\hat{\sigma}_{11}^{z} +$$
$$2\hat{\sigma}_{10}^{z}\hat{\sigma}_{12}^{z} + 2\hat{\sigma}_{10}^{z}\hat{\sigma}_{13}^{z} + 2\hat{\sigma}_{10}^{z}\hat{\sigma}_{14}^{z} + 2\hat{\sigma}_{10}^{z}\hat{\sigma}_{15}^{z} + 2\hat{\sigma}_{10}^{z}\hat{\sigma}_{16}^{z} + 2\hat{\sigma}_{10}^{z}\hat{\sigma}_{17}^{z} + 2\hat{\sigma}_{10}^{z}\hat{\sigma}_{18}^{z} + 2\hat{\sigma}_{10}^{z}\hat{\sigma}_{19}^{z} + 4\hat{\sigma}_{10}^{z}\hat{\sigma}_{2}^{z} + 2\hat{\sigma}_{10}^{z}\hat{\sigma}_{20}^{z} + 2\hat{\sigma}_{10}^{z}\hat{\sigma}_{21}^{z} +$$
$$180\hat{\sigma}_{10}^{z}\hat{\sigma}_{22}^{z} + 2\hat{\sigma}_{10}^{z}\hat{\sigma}_{23}^{z} + 2\hat{\sigma}_{10}^{z}\hat{\sigma}_{24}^{z} + 2\hat{\sigma}_{10}^{z}\hat{\sigma}_{25}^{z} + 2\hat{\sigma}_{10}^{z}\hat{\sigma}_{26}^{z} + 2\hat{\sigma}_{10}^{z}\hat{\sigma}_{27}^{z} + 2\hat{\sigma}_{10}^{z}\hat{\sigma}_{28}^{z} + 2\hat{\sigma}_{10}^{z}\hat{\sigma}_{29}^{z} + 4\hat{\sigma}_{10}^{z}\hat{\sigma}_{3}^{z} + 2\hat{\sigma}_{10}^{z}\hat{\sigma}_{30}^{z} +$$
$$2\hat{\sigma}_{10}^{z}\hat{\sigma}_{31}^{z} + 2\hat{\sigma}_{10}^{z}\hat{\sigma}_{32}^{z} + 2\hat{\sigma}_{10}^{z}\hat{\sigma}_{33}^{z} + 180\hat{\sigma}_{10}^{z}\hat{\sigma}_{34}^{z} + 2\hat{\sigma}_{10}^{z}\hat{\sigma}_{35}^{z} - 4\hat{\sigma}_{10}^{z}\hat{\sigma}_{36}^{z} - 4\hat{\sigma}_{10}^{z}\hat{\sigma}_{37}^{z} - 4\hat{\sigma}_{10}^{z}\hat{\sigma}_{38}^{z} - 4\hat{\sigma}_{10}^{z}\hat{\sigma}_{39}^{z} + 4\hat{\sigma}_{10}^{z}\hat{\sigma}_{4}^{z} -$$
$$4\hat{\sigma}_{10}^{z}\hat{\sigma}_{40}^{z} - 4\hat{\sigma}_{10}^{z}\hat{\sigma}_{41}^{z} - 4\hat{\sigma}_{10}^{z}\hat{\sigma}_{42}^{z} - 4\hat{\sigma}_{10}^{z}\hat{\sigma}_{43}^{z} - 4\hat{\sigma}_{10}^{z}\hat{\sigma}_{44}^{z} - 4\hat{\sigma}_{10}^{z}\hat{\sigma}_{45}^{z} - 360\hat{\sigma}_{10}^{z}\hat{\sigma}_{46}^{z} - 4\hat{\sigma}_{10}^{z}\hat{\sigma}_{47}^{z} - 4\hat{\sigma}_{10}^{z}\hat{\sigma}_{48}^{z} - 4\hat{\sigma}_{10}^{z}\hat{\sigma}_{49}^{z} +$$
$$4\hat{\sigma}_{10}^{z}\hat{\sigma}_{5}^{z} - 4\hat{\sigma}_{10}^{z}\hat{\sigma}_{50}^{z} - 4\hat{\sigma}_{10}^{z}\hat{\sigma}_{51}^{z} - 4\hat{\sigma}_{10}^{z}\hat{\sigma}_{52}^{z} - 4\hat{\sigma}_{10}^{z}\hat{\sigma}_{53}^{z} - 4\hat{\sigma}_{10}^{z}\hat{\sigma}_{54}^{z} - 4\hat{\sigma}_{10}^{z}\hat{\sigma}_{55}^{z} - 4\hat{\sigma}_{10}^{z}\hat{\sigma}_{56}^{z} - 4\hat{\sigma}_{10}^{z}\hat{\sigma}_{57}^{z} - 360\hat{\sigma}_{10}^{z}\hat{\sigma}_{58}^{z} -$$
$$4\hat{\sigma}_{10}^{z}\hat{\sigma}_{59}^{z} + 4\hat{\sigma}_{10}^{z}\hat{\sigma}_{6}^{z} + 4\hat{\sigma}_{10}^{z}\hat{\sigma}_{7}^{z} + 4\hat{\sigma}_{10}^{z}\hat{\sigma}_{8}^{z} + 4\hat{\sigma}_{10}^{z}\hat{\sigma}_{9}^{z} + 404\hat{\sigma}_{10}^{z} + 2\hat{\sigma}_{11}^{z}\hat{\sigma}_{12}^{z} + 2\hat{\sigma}_{11}^{z}\hat{\sigma}_{13}^{z} + 2\hat{\sigma}_{11}^{z}\hat{\sigma}_{14}^{z} + 2\hat{\sigma}_{11}^{z}\hat{\sigma}_{15}^{z} + 2\hat{\sigma}_{11}^{z}\hat{\sigma}_{16}^{z} +$$
$$2\hat{\sigma}_{11}^{z}\hat{\sigma}_{17}^{z} + 2\hat{\sigma}_{11}^{z}\hat{\sigma}_{18}^{z} + 2\hat{\sigma}_{11}^{z}\hat{\sigma}_{19}^{z} + 4\hat{\sigma}_{11}^{z}\hat{\sigma}_{2}^{z} + 2\hat{\sigma}_{11}^{z}\hat{\sigma}_{20}^{z} + 2\hat{\sigma}_{11}^{z}\hat{\sigma}_{21}^{z} + 2\hat{\sigma}_{11}^{z}\hat{\sigma}_{22}^{z} + 180\hat{\sigma}_{11}^{z}\hat{\sigma}_{23}^{z} + 2\hat{\sigma}_{11}^{z}\hat{\sigma}_{24}^{z} + 2\hat{\sigma}_{11}^{z}\hat{\sigma}_{25}^{z} +$$
$$2\hat{\sigma}_{11}^{z}\hat{\sigma}_{26}^{z} + 2\hat{\sigma}_{11}^{z}\hat{\sigma}_{27}^{z} + 2\hat{\sigma}_{11}^{z}\hat{\sigma}_{28}^{z} + 2\hat{\sigma}_{11}^{z}\hat{\sigma}_{29}^{z} + 4\hat{\sigma}_{11}^{z}\hat{\sigma}_{3}^{z} + 2\hat{\sigma}_{11}^{z}\hat{\sigma}_{30}^{z} + 2\hat{\sigma}_{11}^{z}\hat{\sigma}_{31}^{z} + 2\hat{\sigma}_{11}^{z}\hat{\sigma}_{32}^{z} + 2\hat{\sigma}_{11}^{z}\hat{\sigma}_{33}^{z} + 2\hat{\sigma}_{11}^{z}\hat{\sigma}_{34}^{z} +$$
$$180\hat{\sigma}_{11}^{z}\hat{\sigma}_{35}^{z} - 4\hat{\sigma}_{11}^{z}\hat{\sigma}_{36}^{z} - 4\hat{\sigma}_{11}^{z}\hat{\sigma}_{37}^{z} - 4\hat{\sigma}_{11}^{z}\hat{\sigma}_{38}^{z} - 4\hat{\sigma}_{11}^{z}\hat{\sigma}_{39}^{z} + 4\hat{\sigma}_{11}^{z}\hat{\sigma}_{4}^{z} - 4\hat{\sigma}_{11}^{z}\hat{\sigma}_{40}^{z} - 4\hat{\sigma}_{11}^{z}\hat{\sigma}_{41}^{z} - 4\hat{\sigma}_{11}^{z}\hat{\sigma}_{42}^{z} - 4\hat{\sigma}_{11}^{z}\hat{\sigma}_{43}^{z} -$$
$$4\hat{\sigma}_{11}^{z}\hat{\sigma}_{44}^{z} - 4\hat{\sigma}_{11}^{z}\hat{\sigma}_{45}^{z} - 4\hat{\sigma}_{11}^{z}\hat{\sigma}_{46}^{z} - 360\hat{\sigma}_{11}^{z}\hat{\sigma}_{47}^{z} - 4\hat{\sigma}_{11}^{z}\hat{\sigma}_{48}^{z} - 4\hat{\sigma}_{11}^{z}\hat{\sigma}_{49}^{z} + 4\hat{\sigma}_{11}^{z}\hat{\sigma}_{5}^{z} - 4\hat{\sigma}_{11}^{z}\hat{\sigma}_{50}^{z} - 4\hat{\sigma}_{11}^{z}\hat{\sigma}_{51}^{z} - 4\hat{\sigma}_{11}^{z}\hat{\sigma}_{52}^{z} -$$
$$4\hat{\sigma}_{11}^{z}\hat{\sigma}_{53}^{z} - 4\hat{\sigma}_{11}^{z}\hat{\sigma}_{54}^{z} - 4\hat{\sigma}_{11}^{z}\hat{\sigma}_{55}^{z} - 4\hat{\sigma}_{11}^{z}\hat{\sigma}_{56}^{z} - 4\hat{\sigma}_{11}^{z}\hat{\sigma}_{57}^{z} - 4\hat{\sigma}_{11}^{z}\hat{\sigma}_{58}^{z} - 360\hat{\sigma}_{11}^{z}\hat{\sigma}_{59}^{z} + 4\hat{\sigma}_{11}^{z}\hat{\sigma}_{6}^{z} + 4\hat{\sigma}_{11}^{z}\hat{\sigma}_{7}^{z} + 4\hat{\sigma}_{11}^{z}\hat{\sigma}_{8}^{z} +$$
$$4\hat{\sigma}_{11}^{z}\hat{\sigma}_{9}^{z} + 404\hat{\sigma}_{11}^{z} + 4\hat{\sigma}_{12}^{z}\hat{\sigma}_{13}^{z} + 4\hat{\sigma}_{12}^{z}\hat{\sigma}_{14}^{z} + 4\hat{\sigma}_{12}^{z}\hat{\sigma}_{15}^{z} + 4\hat{\sigma}_{12}^{z}\hat{\sigma}_{16}^{z} + 4\hat{\sigma}_{12}^{z}\hat{\sigma}_{17}^{z} + 4\hat{\sigma}_{12}^{z}\hat{\sigma}_{18}^{z} + 4\hat{\sigma}_{12}^{z}\hat{\sigma}_{19}^{z} + 2\hat{\sigma}_{12}^{z}\hat{\sigma}_{2}^{z} + 4\hat{\sigma}_{12}^{z}\hat{\sigma}_{20}^{z} +$$
$$4\hat{\sigma}_{12}^{z}\hat{\sigma}_{21}^{z} + 4\hat{\sigma}_{12}^{z}\hat{\sigma}_{22}^{z} + 4\hat{\sigma}_{12}^{z}\hat{\sigma}_{23}^{z} + 180\hat{\sigma}_{12}^{z}\hat{\sigma}_{24}^{z} + 2\hat{\sigma}_{12}^{z}\hat{\sigma}_{25}^{z} + 2\hat{\sigma}_{12}^{z}\hat{\sigma}_{26}^{z} + 2\hat{\sigma}_{12}^{z}\hat{\sigma}_{27}^{z} + 2\hat{\sigma}_{12}^{z}\hat{\sigma}_{28}^{z} + 2\hat{\sigma}_{12}^{z}\hat{\sigma}_{29}^{z} + 2\hat{\sigma}_{12}^{z}\hat{\sigma}_{3}^{z} +$$
$$2\hat{\sigma}_{12}^{z}\hat{\sigma}_{30}^{z} + 2\hat{\sigma}_{12}^{z}\hat{\sigma}_{31}^{z} + 2\hat{\sigma}_{12}^{z}\hat{\sigma}_{32}^{z} + 2\hat{\sigma}_{12}^{z}\hat{\sigma}_{33}^{z} + 2\hat{\sigma}_{12}^{z}\hat{\sigma}_{34}^{z} + 2\hat{\sigma}_{12}^{z}\hat{\sigma}_{35}^{z} - 360\hat{\sigma}_{12}^{z}\hat{\sigma}_{36}^{z} - 4\hat{\sigma}_{12}^{z}\hat{\sigma}_{37}^{z} - 4\hat{\sigma}_{12}^{z}\hat{\sigma}_{38}^{z} - 4\hat{\sigma}_{12}^{z}\hat{\sigma}_{39}^{z} +$$
$$2\hat{\sigma}_{12}^{z}\hat{\sigma}_{4}^{z} - 4\hat{\sigma}_{12}^{z}\hat{\sigma}_{40}^{z} - 4\hat{\sigma}_{12}^{z}\hat{\sigma}_{41}^{z} - 4\hat{\sigma}_{12}^{z}\hat{\sigma}_{42}^{z} - 4\hat{\sigma}_{12}^{z}\hat{\sigma}_{43}^{z} - 4\hat{\sigma}_{12}^{z}\hat{\sigma}_{44}^{z} - 4\hat{\sigma}_{12}^{z}\hat{\sigma}_{45}^{z} - 4\hat{\sigma}_{12}^{z}\hat{\sigma}_{46}^{z} - 4\hat{\sigma}_{12}^{z}\hat{\sigma}_{47}^{z} + 2\hat{\sigma}_{12}^{z}\hat{\sigma}_{5}^{z} + 2\hat{\sigma}_{12}^{z}\hat{\sigma}_{6}^{z} -$$
$$360\hat{\sigma}_{12}^{z}\hat{\sigma}_{60}^{z} - 4\hat{\sigma}_{12}^{z}\hat{\sigma}_{61}^{z} - 4\hat{\sigma}_{12}^{z}\hat{\sigma}_{62}^{z} - 4\hat{\sigma}_{12}^{z}\hat{\sigma}_{63}^{z} - 4\hat{\sigma}_{12}^{z}\hat{\sigma}_{64}^{z} - 4\hat{\sigma}_{12}^{z}\hat{\sigma}_{65}^{z} - 4\hat{\sigma}_{12}^{z}\hat{\sigma}_{66}^{z} - 4\hat{\sigma}_{12}^{z}\hat{\sigma}_{67}^{z} - 4\hat{\sigma}_{12}^{z}\hat{\sigma}_{68}^{z} - 4\hat{\sigma}_{12}^{z}\hat{\sigma}_{69}^{z} +$$
$$2\hat{\sigma}_{12}^{z}\hat{\sigma}_{7}^{z} - 4\hat{\sigma}_{12}^{z}\hat{\sigma}_{70}^{z} - 4\hat{\sigma}_{12}^{z}\hat{\sigma}_{71}^{z} + 2\hat{\sigma}_{12}^{z}\hat{\sigma}_{8}^{z} + 2\hat{\sigma}_{12}^{z}\hat{\sigma}_{9}^{z} + 404\hat{\sigma}_{12}^{z} + 4\hat{\sigma}_{13}^{z}\hat{\sigma}_{14}^{z} + 4\hat{\sigma}_{13}^{z}\hat{\sigma}_{15}^{z} + 4\hat{\sigma}_{13}^{z}\hat{\sigma}_{16}^{z} + 4\hat{\sigma}_{13}^{z}\hat{\sigma}_{17}^{z} + 4\hat{\sigma}_{13}^{z}\hat{\sigma}_{18}^{z} +$$
$$4\hat{\sigma}_{13}^{z}\hat{\sigma}_{19}^{z} + 2\hat{\sigma}_{13}^{z}\hat{\sigma}_{2}^{z} + 4\hat{\sigma}_{13}^{z}\hat{\sigma}_{20}^{z} + 4\hat{\sigma}_{13}^{z}\hat{\sigma}_{21}^{z} + 4\hat{\sigma}_{13}^{z}\hat{\sigma}_{22}^{z} + 4\hat{\sigma}_{13}^{z}\hat{\sigma}_{23}^{z} + 2\hat{\sigma}_{13}^{z}\hat{\sigma}_{24}^{z} + 180\hat{\sigma}_{13}^{z}\hat{\sigma}_{25}^{z} + 2\hat{\sigma}_{13}^{z}\hat{\sigma}_{26}^{z} + 2\hat{\sigma}_{13}^{z}\hat{\sigma}_{27}^{z} +$$
$$2\hat{\sigma}_{13}^{z}\hat{\sigma}_{28}^{z} + 2\hat{\sigma}_{13}^{z}\hat{\sigma}_{29}^{z} + 2\hat{\sigma}_{13}^{z}\hat{\sigma}_{3}^{z} + 2\hat{\sigma}_{13}^{z}\hat{\sigma}_{30}^{z} + 2\hat{\sigma}_{13}^{z}\hat{\sigma}_{31}^{z} + 2\hat{\sigma}_{13}^{z}\hat{\sigma}_{32}^{z} + 2\hat{\sigma}_{13}^{z}\hat{\sigma}_{33}^{z} + 2\hat{\sigma}_{13}^{z}\hat{\sigma}_{34}^{z} + 2\hat{\sigma}_{13}^{z}\hat{\sigma}_{35}^{z} - 4\hat{\sigma}_{13}^{z}\hat{\sigma}_{36}^{z} -$$
$$360\hat{\sigma}_{13}^{z}\hat{\sigma}_{37}^{z} - 4\hat{\sigma}_{13}^{z}\hat{\sigma}_{38}^{z} - 4\hat{\sigma}_{13}^{z}\hat{\sigma}_{39}^{z} + 2\hat{\sigma}_{13}^{z}\hat{\sigma}_{4}^{z} - 4\hat{\sigma}_{13}^{z}\hat{\sigma}_{40}^{z} - 4\hat{\sigma}_{13}^{z}\hat{\sigma}_{41}^{z} - 4\hat{\sigma}_{13}^{z}\hat{\sigma}_{42}^{z} - 4\hat{\sigma}_{13}^{z}\hat{\sigma}_{43}^{z} - 4\hat{\sigma}_{13}^{z}\hat{\sigma}_{44}^{z} - 4\hat{\sigma}_{13}^{z}\hat{\sigma}_{45}^{z} -$$
$$4\hat{\sigma}_{13}^{z}\hat{\sigma}_{46}^{z} - 4\hat{\sigma}_{13}^{z}\hat{\sigma}_{47}^{z} + 2\hat{\sigma}_{13}^{z}\hat{\sigma}_{5}^{z} + 2\hat{\sigma}_{13}^{z}\hat{\sigma}_{6}^{z} - 4\hat{\sigma}_{13}^{z}\hat{\sigma}_{60}^{z} - 360\hat{\sigma}_{13}^{z}\hat{\sigma}_{61}^{z} - 4\hat{\sigma}_{13}^{z}\hat{\sigma}_{62}^{z} - 4\hat{\sigma}_{13}^{z}\hat{\sigma}_{63}^{z} - 4\hat{\sigma}_{13}^{z}\hat{\sigma}_{64}^{z} - 4\hat{\sigma}_{13}^{z}\hat{\sigma}_{65}^{z} -$$
$$4\hat{\sigma}_{13}^{z}\hat{\sigma}_{66}^{z} - 4\hat{\sigma}_{13}^{z}\hat{\sigma}_{67}^{z} - 4\hat{\sigma}_{13}^{z}\hat{\sigma}_{68}^{z} - 4\hat{\sigma}_{13}^{z}\hat{\sigma}_{69}^{z} + 2\hat{\sigma}_{13}^{z}\hat{\sigma}_{7}^{z} - 4\hat{\sigma}_{13}^{z}\hat{\sigma}_{70}^{z} - 4\hat{\sigma}_{13}^{z}\hat{\sigma}_{71}^{z} + 2\hat{\sigma}_{13}^{z}\hat{\sigma}_{8}^{z} + 2\hat{\sigma}_{13}^{z}\hat{\sigma}_{9}^{z} + 404\hat{\sigma}_{13}^{z} + 4\hat{\sigma}_{14}^{z}\hat{\sigma}_{15}^{z} +$$
$$4\hat{\sigma}_{14}^{z}\hat{\sigma}_{16}^{z} + 4\hat{\sigma}_{14}^{z}\hat{\sigma}_{17}^{z} + 4\hat{\sigma}_{14}^{z}\hat{\sigma}_{18}^{z} + 4\hat{\sigma}_{14}^{z}\hat{\sigma}_{19}^{z} + 180\hat{\sigma}_{14}^{z}\hat{\sigma}_{2}^{z} + 4\hat{\sigma}_{14}^{z}\hat{\sigma}_{20}^{z} + 4\hat{\sigma}_{14}^{z}\hat{\sigma}_{21}^{z} + 4\hat{\sigma}_{14}^{z}\hat{\sigma}_{22}^{z} + 4\hat{\sigma}_{14}^{z}\hat{\sigma}_{23}^{z} + 2\hat{\sigma}_{14}^{z}\hat{\sigma}_{24}^{z} +$$
$$2\hat{\sigma}_{14}^{z}\hat{\sigma}_{25}^{z} + 180\hat{\sigma}_{14}^{z}\hat{\sigma}_{26}^{z} + 2\hat{\sigma}_{14}^{z}\hat{\sigma}_{27}^{z} + 2\hat{\sigma}_{14}^{z}\hat{\sigma}_{28}^{z} + 2\hat{\sigma}_{14}^{z}\hat{\sigma}_{29}^{z} + 2\hat{\sigma}_{14}^{z}\hat{\sigma}_{3}^{z} + 2\hat{\sigma}_{14}^{z}\hat{\sigma}_{30}^{z} + 2\hat{\sigma}_{14}^{z}\hat{\sigma}_{31}^{z} + 2\hat{\sigma}_{14}^{z}\hat{\sigma}_{32}^{z} + 2\hat{\sigma}_{14}^{z}\hat{\sigma}_{33}^{z} +$$
$$2\hat{\sigma}_{14}^{z}\hat{\sigma}_{34}^{z} + 2\hat{\sigma}_{14}^{z}\hat{\sigma}_{35}^{z} - 4\hat{\sigma}_{14}^{z}\hat{\sigma}_{36}^{z} - 4\hat{\sigma}_{14}^{z}\hat{\sigma}_{37}^{z} - 360\hat{\sigma}_{14}^{z}\hat{\sigma}_{38}^{z} - 4\hat{\sigma}_{14}^{z}\hat{\sigma}_{39}^{z} + 2\hat{\sigma}_{14}^{z}\hat{\sigma}_{4}^{z} - 4\hat{\sigma}_{14}^{z}\hat{\sigma}_{40}^{z} - 4\hat{\sigma}_{14}^{z}\hat{\sigma}_{41}^{z} - 4\hat{\sigma}_{14}^{z}\hat{\sigma}_{42}^{z} -$$
$$4\hat{\sigma}_{14}^{z}\hat{\sigma}_{43}^{z} - 4\hat{\sigma}_{14}^{z}\hat{\sigma}_{44}^{z} - 4\hat{\sigma}_{14}^{z}\hat{\sigma}_{45}^{z} - 4\hat{\sigma}_{14}^{z}\hat{\sigma}_{46}^{z} - 4\hat{\sigma}_{14}^{z}\hat{\sigma}_{47}^{z} + 2\hat{\sigma}_{14}^{z}\hat{\sigma}_{5}^{z} + 2\hat{\sigma}_{14}^{z}\hat{\sigma}_{6}^{z} - 4\hat{\sigma}_{14}^{z}\hat{\sigma}_{60}^{z} - 4\hat{\sigma}_{14}^{z}\hat{\sigma}_{61}^{z} - 360\hat{\sigma}_{14}^{z}\hat{\sigma}_{62}^{z} -$$
$$4\hat{\sigma}_{14}^{z}\hat{\sigma}_{63}^{z} - 4\hat{\sigma}_{14}^{z}\hat{\sigma}_{64}^{z} - 4\hat{\sigma}_{14}^{z}\hat{\sigma}_{65}^{z} - 4\hat{\sigma}_{14}^{z}\hat{\sigma}_{66}^{z} - 4\hat{\sigma}_{14}^{z}\hat{\sigma}_{67}^{z} - 4\hat{\sigma}_{14}^{z}\hat{\sigma}_{68}^{z} - 4\hat{\sigma}_{14}^{z}\hat{\sigma}_{69}^{z} + 2\hat{\sigma}_{14}^{z}\hat{\sigma}_{7}^{z} - 4\hat{\sigma}_{14}^{z}\hat{\sigma}_{70}^{z} - 4\hat{\sigma}_{14}^{z}\hat{\sigma}_{71}^{z} +$$
$$2\hat{\sigma}_{14}^{z}\hat{\sigma}_{8}^{z} + 2\hat{\sigma}_{14}^{z}\hat{\sigma}_{9}^{z} + 404\hat{\sigma}_{14}^{z} + 4\hat{\sigma}_{15}^{z}\hat{\sigma}_{16}^{z} + 4\hat{\sigma}_{15}^{z}\hat{\sigma}_{17}^{z} + 4\hat{\sigma}_{15}^{z}\hat{\sigma}_{18}^{z} + 4\hat{\sigma}_{15}^{z}\hat{\sigma}_{19}^{z} + 2\hat{\sigma}_{15}^{z}\hat{\sigma}_{2}^{z} + 4\hat{\sigma}_{15}^{z}\hat{\sigma}_{20}^{z} + 4\hat{\sigma}_{15}^{z}\hat{\sigma}_{21}^{z} + 4\hat{\sigma}_{15}^{z}\hat{\sigma}_{22}^{z} +$$
$$4\hat{\sigma}_{15}^{z}\hat{\sigma}_{23}^{z} + 2\hat{\sigma}_{15}^{z}\hat{\sigma}_{24}^{z} + 2\hat{\sigma}_{15}^{z}\hat{\sigma}_{25}^{z} + 2\hat{\sigma}_{15}^{z}\hat{\sigma}_{26}^{z} + 180\hat{\sigma}_{15}^{z}\hat{\sigma}_{27}^{z} + 2\hat{\sigma}_{15}^{z}\hat{\sigma}_{28}^{z} + 2\hat{\sigma}_{15}^{z}\hat{\sigma}_{29}^{z} + 180\hat{\sigma}_{15}^{z}\hat{\sigma}_{3}^{z} + 2\hat{\sigma}_{15}^{z}\hat{\sigma}_{30}^{z} + 2\hat{\sigma}_{15}^{z}\hat{\sigma}_{31}^{z} +$$
$$2\hat{\sigma}_{15}^{z}\hat{\sigma}_{32}^{z} + 2\hat{\sigma}_{15}^{z}\hat{\sigma}_{33}^{z} + 2\hat{\sigma}_{15}^{z}\hat{\sigma}_{34}^{z} + 2\hat{\sigma}_{15}^{z}\hat{\sigma}_{35}^{z} - 4\hat{\sigma}_{15}^{z}\hat{\sigma}_{36}^{z} - 4\hat{\sigma}_{15}^{z}\hat{\sigma}_{37}^{z} - 4\hat{\sigma}_{15}^{z}\hat{\sigma}_{38}^{z} - 360\hat{\sigma}_{15}^{z}\hat{\sigma}_{39}^{z} + 2\hat{\sigma}_{15}^{z}\hat{\sigma}_{4}^{z} - 4\hat{\sigma}_{15}^{z}\hat{\sigma}_{40}^{z} -$$
$$4\hat{\sigma}_{15}^{z}\hat{\sigma}_{41}^{z} - 4\hat{\sigma}_{15}^{z}\hat{\sigma}_{42}^{z} - 4\hat{\sigma}_{15}^{z}\hat{\sigma}_{43}^{z} - 4\hat{\sigma}_{15}^{z}\hat{\sigma}_{44}^{z} - 4\hat{\sigma}_{15}^{z}\hat{\sigma}_{45}^{z} - 4\hat{\sigma}_{15}^{z}\hat{\sigma}_{46}^{z} - 4\hat{\sigma}_{15}^{z}\hat{\sigma}_{47}^{z} + 2\hat{\sigma}_{15}^{z}\hat{\sigma}_{5}^{z} + 2\hat{\sigma}_{15}^{z}\hat{\sigma}_{6}^{z} - 4\hat{\sigma}_{15}^{z}\hat{\sigma}_{60}^{z} - 4\hat{\sigma}_{15}^{z}\hat{\sigma}_{61}^{z} -$$
$$4\hat{\sigma}_{15}^{z}\hat{\sigma}_{62}^{z} - 360\hat{\sigma}_{15}^{z}\hat{\sigma}_{63}^{z} - 4\hat{\sigma}_{15}^{z}\hat{\sigma}_{64}^{z} - 4\hat{\sigma}_{15}^{z}\hat{\sigma}_{65}^{z} - 4\hat{\sigma}_{15}^{z}\hat{\sigma}_{66}^{z} - 4\hat{\sigma}_{15}^{z}\hat{\sigma}_{67}^{z} - 4\hat{\sigma}_{15}^{z}\hat{\sigma}_{68}^{z} - 4\hat{\sigma}_{15}^{z}\hat{\sigma}_{69}^{z} + 2\hat{\sigma}_{15}^{z}\hat{\sigma}_{7}^{z} - 4\hat{\sigma}_{15}^{z}\hat{\sigma}_{70}^{z} -$$
$$4\hat{\sigma}_{15}^{z}\hat{\sigma}_{71}^{z} + 2\hat{\sigma}_{15}^{z}\hat{\sigma}_{8}^{z} + 2\hat{\sigma}_{15}^{z}\hat{\sigma}_{9}^{z} + 404\hat{\sigma}_{15}^{z} + 4\hat{\sigma}_{16}^{z}\hat{\sigma}_{17}^{z} + 4\hat{\sigma}_{16}^{z}\hat{\sigma}_{18}^{z} + 4\hat{\sigma}_{16}^{z}\hat{\sigma}_{19}^{z} + 2\hat{\sigma}_{16}^{z}\hat{\sigma}_{2}^{z} + 4\hat{\sigma}_{16}^{z}\hat{\sigma}_{20}^{z} + 4\hat{\sigma}_{16}^{z}\hat{\sigma}_{21}^{z} + 4\hat{\sigma}_{16}^{z}\hat{\sigma}_{22}^{z} +$$
$$4\hat{\sigma}_{16}^{z}\hat{\sigma}_{23}^{z} + 2\hat{\sigma}_{16}^{z}\hat{\sigma}_{24}^{z} + 2\hat{\sigma}_{16}^{z}\hat{\sigma}_{25}^{z} + 2\hat{\sigma}_{16}^{z}\hat{\sigma}_{26}^{z} + 2\hat{\sigma}_{16}^{z}\hat{\sigma}_{27}^{z} + 180\hat{\sigma}_{16}^{z}\hat{\sigma}_{28}^{z} + 2\hat{\sigma}_{16}^{z}\hat{\sigma}_{29}^{z} + 2\hat{\sigma}_{16}^{z}\hat{\sigma}_{3}^{z} + 2\hat{\sigma}_{16}^{z}\hat{\sigma}_{30}^{z} + 2\hat{\sigma}_{16}^{z}\hat{\sigma}_{31}^{z} +$$
$$2\hat{\sigma}_{16}^{z}\hat{\sigma}_{32}^{z} + 2\hat{\sigma}_{16}^{z}\hat{\sigma}_{33}^{z} + 2\hat{\sigma}_{16}^{z}\hat{\sigma}_{34}^{z} + 2\hat{\sigma}_{16}^{z}\hat{\sigma}_{35}^{z} - 4\hat{\sigma}_{16}^{z}\hat{\sigma}_{36}^{z} - 4\hat{\sigma}_{16}^{z}\hat{\sigma}_{37}^{z} - 4\hat{\sigma}_{16}^{z}\hat{\sigma}_{38}^{z} - 4\hat{\sigma}_{16}^{z}\hat{\sigma}_{39}^{z} + 180\hat{\sigma}_{16}^{z}\hat{\sigma}_{4}^{z} - 360\hat{\sigma}_{16}^{z}\hat{\sigma}_{40}^{z} -$$

$4\hat{\sigma}^z_{16}\hat{\sigma}^z_{41} - 4\hat{\sigma}^z_{16}\hat{\sigma}^z_{42} - 4\hat{\sigma}^z_{16}\hat{\sigma}^z_{43} - 4\hat{\sigma}^z_{16}\hat{\sigma}^z_{44} - 4\hat{\sigma}^z_{16}\hat{\sigma}^z_{45} - 4\hat{\sigma}^z_{16}\hat{\sigma}^z_{46} - 4\hat{\sigma}^z_{16}\hat{\sigma}^z_{47} + 2\hat{\sigma}^z_{16}\hat{\sigma}^z_{5} + 2\hat{\sigma}^z_{16}\hat{\sigma}^z_{6} - 4\hat{\sigma}^z_{16}\hat{\sigma}^z_{60} - 4\hat{\sigma}^z_{16}\hat{\sigma}^z_{61} -$
$4\hat{\sigma}^z_{16}\hat{\sigma}^z_{62} - 4\hat{\sigma}^z_{16}\hat{\sigma}^z_{63} - 360\hat{\sigma}^z_{16}\hat{\sigma}^z_{64} - 4\hat{\sigma}^z_{16}\hat{\sigma}^z_{65} - 4\hat{\sigma}^z_{16}\hat{\sigma}^z_{66} - 4\hat{\sigma}^z_{16}\hat{\sigma}^z_{67} - 4\hat{\sigma}^z_{16}\hat{\sigma}^z_{68} - 4\hat{\sigma}^z_{16}\hat{\sigma}^z_{69} + 2\hat{\sigma}^z_{16}\hat{\sigma}^z_{7} - 4\hat{\sigma}^z_{16}\hat{\sigma}^z_{70} -$
$4\hat{\sigma}^z_{16}\hat{\sigma}^z_{71} + 2\hat{\sigma}^z_{16}\hat{\sigma}^z_{8} + 2\hat{\sigma}^z_{16}\hat{\sigma}^z_{9} + 404\hat{\sigma}^z_{16} + 4\hat{\sigma}^z_{17}\hat{\sigma}^z_{18} + 4\hat{\sigma}^z_{17}\hat{\sigma}^z_{19} + 2\hat{\sigma}^z_{17}\hat{\sigma}^z_{2} + 4\hat{\sigma}^z_{17}\hat{\sigma}^z_{20} + 4\hat{\sigma}^z_{17}\hat{\sigma}^z_{21} + 4\hat{\sigma}^z_{17}\hat{\sigma}^z_{22} + 4\hat{\sigma}^z_{17}\hat{\sigma}^z_{23} +$
$2\hat{\sigma}^z_{17}\hat{\sigma}^z_{24} + 2\hat{\sigma}^z_{17}\hat{\sigma}^z_{25} + 2\hat{\sigma}^z_{17}\hat{\sigma}^z_{26} + 2\hat{\sigma}^z_{17}\hat{\sigma}^z_{27} + 2\hat{\sigma}^z_{17}\hat{\sigma}^z_{28} + 180\hat{\sigma}^z_{17}\hat{\sigma}^z_{29} + 2\hat{\sigma}^z_{17}\hat{\sigma}^z_{3} + 2\hat{\sigma}^z_{17}\hat{\sigma}^z_{30} + 2\hat{\sigma}^z_{17}\hat{\sigma}^z_{31} + 2\hat{\sigma}^z_{17}\hat{\sigma}^z_{32} +$
$2\hat{\sigma}^z_{17}\hat{\sigma}^z_{33} + 2\hat{\sigma}^z_{17}\hat{\sigma}^z_{34} + 2\hat{\sigma}^z_{17}\hat{\sigma}^z_{35} - 4\hat{\sigma}^z_{17}\hat{\sigma}^z_{36} - 4\hat{\sigma}^z_{17}\hat{\sigma}^z_{37} - 4\hat{\sigma}^z_{17}\hat{\sigma}^z_{38} - 4\hat{\sigma}^z_{17}\hat{\sigma}^z_{39} + 2\hat{\sigma}^z_{17}\hat{\sigma}^z_{4} - 4\hat{\sigma}^z_{17}\hat{\sigma}^z_{40} - 360\hat{\sigma}^z_{17}\hat{\sigma}^z_{41} -$
$4\hat{\sigma}^z_{17}\hat{\sigma}^z_{42} - 4\hat{\sigma}^z_{17}\hat{\sigma}^z_{43} - 4\hat{\sigma}^z_{17}\hat{\sigma}^z_{44} - 4\hat{\sigma}^z_{17}\hat{\sigma}^z_{45} - 4\hat{\sigma}^z_{17}\hat{\sigma}^z_{46} - 4\hat{\sigma}^z_{17}\hat{\sigma}^z_{47} + 180\hat{\sigma}^z_{17}\hat{\sigma}^z_{5} + 2\hat{\sigma}^z_{17}\hat{\sigma}^z_{6} - 4\hat{\sigma}^z_{17}\hat{\sigma}^z_{60} - 4\hat{\sigma}^z_{17}\hat{\sigma}^z_{61} -$
$4\hat{\sigma}^z_{17}\hat{\sigma}^z_{62} - 4\hat{\sigma}^z_{17}\hat{\sigma}^z_{63} - 4\hat{\sigma}^z_{17}\hat{\sigma}^z_{64} - 360\hat{\sigma}^z_{17}\hat{\sigma}^z_{65} - 4\hat{\sigma}^z_{17}\hat{\sigma}^z_{66} - 4\hat{\sigma}^z_{17}\hat{\sigma}^z_{67} - 4\hat{\sigma}^z_{17}\hat{\sigma}^z_{68} - 4\hat{\sigma}^z_{17}\hat{\sigma}^z_{69} + 2\hat{\sigma}^z_{17}\hat{\sigma}^z_{7} - 4\hat{\sigma}^z_{17}\hat{\sigma}^z_{70} -$
$4\hat{\sigma}^z_{17}\hat{\sigma}^z_{71} + 2\hat{\sigma}^z_{17}\hat{\sigma}^z_{8} + 2\hat{\sigma}^z_{17}\hat{\sigma}^z_{9} + 404\hat{\sigma}^z_{17} + 4\hat{\sigma}^z_{18}\hat{\sigma}^z_{19} + 2\hat{\sigma}^z_{18}\hat{\sigma}^z_{2} + 4\hat{\sigma}^z_{18}\hat{\sigma}^z_{20} + 4\hat{\sigma}^z_{18}\hat{\sigma}^z_{21} + 4\hat{\sigma}^z_{18}\hat{\sigma}^z_{22} + 4\hat{\sigma}^z_{18}\hat{\sigma}^z_{23} + 2\hat{\sigma}^z_{18}\hat{\sigma}^z_{24} +$
$2\hat{\sigma}^z_{18}\hat{\sigma}^z_{25} + 2\hat{\sigma}^z_{18}\hat{\sigma}^z_{26} + 2\hat{\sigma}^z_{18}\hat{\sigma}^z_{27} + 2\hat{\sigma}^z_{18}\hat{\sigma}^z_{28} + 2\hat{\sigma}^z_{18}\hat{\sigma}^z_{29} + 2\hat{\sigma}^z_{18}\hat{\sigma}^z_{3} + 180\hat{\sigma}^z_{18}\hat{\sigma}^z_{30} + 2\hat{\sigma}^z_{18}\hat{\sigma}^z_{31} + 2\hat{\sigma}^z_{18}\hat{\sigma}^z_{32} + 2\hat{\sigma}^z_{18}\hat{\sigma}^z_{33} +$
$2\hat{\sigma}^z_{18}\hat{\sigma}^z_{34} + 2\hat{\sigma}^z_{18}\hat{\sigma}^z_{35} - 4\hat{\sigma}^z_{18}\hat{\sigma}^z_{36} - 4\hat{\sigma}^z_{18}\hat{\sigma}^z_{37} - 4\hat{\sigma}^z_{18}\hat{\sigma}^z_{38} - 4\hat{\sigma}^z_{18}\hat{\sigma}^z_{39} + 2\hat{\sigma}^z_{18}\hat{\sigma}^z_{4} - 4\hat{\sigma}^z_{18}\hat{\sigma}^z_{40} - 4\hat{\sigma}^z_{18}\hat{\sigma}^z_{41} - 360\hat{\sigma}^z_{18}\hat{\sigma}^z_{42} -$
$4\hat{\sigma}^z_{18}\hat{\sigma}^z_{43} - 4\hat{\sigma}^z_{18}\hat{\sigma}^z_{44} - 4\hat{\sigma}^z_{18}\hat{\sigma}^z_{45} - 4\hat{\sigma}^z_{18}\hat{\sigma}^z_{46} - 4\hat{\sigma}^z_{18}\hat{\sigma}^z_{47} + 2\hat{\sigma}^z_{18}\hat{\sigma}^z_{5} + 180\hat{\sigma}^z_{18}\hat{\sigma}^z_{6} - 4\hat{\sigma}^z_{18}\hat{\sigma}^z_{60} - 4\hat{\sigma}^z_{18}\hat{\sigma}^z_{61} - 4\hat{\sigma}^z_{18}\hat{\sigma}^z_{62} -$
$4\hat{\sigma}^z_{18}\hat{\sigma}^z_{63} - 4\hat{\sigma}^z_{18}\hat{\sigma}^z_{64} - 4\hat{\sigma}^z_{18}\hat{\sigma}^z_{65} - 360\hat{\sigma}^z_{18}\hat{\sigma}^z_{66} - 4\hat{\sigma}^z_{18}\hat{\sigma}^z_{67} - 4\hat{\sigma}^z_{18}\hat{\sigma}^z_{68} - 4\hat{\sigma}^z_{18}\hat{\sigma}^z_{69} + 2\hat{\sigma}^z_{18}\hat{\sigma}^z_{7} - 4\hat{\sigma}^z_{18}\hat{\sigma}^z_{70} - 4\hat{\sigma}^z_{18}\hat{\sigma}^z_{71} +$
$2\hat{\sigma}^z_{18}\hat{\sigma}^z_{8} + 2\hat{\sigma}^z_{18}\hat{\sigma}^z_{9} + 404\hat{\sigma}^z_{18} + 2\hat{\sigma}^z_{19}\hat{\sigma}^z_{2} + 4\hat{\sigma}^z_{19}\hat{\sigma}^z_{20} + 4\hat{\sigma}^z_{19}\hat{\sigma}^z_{21} + 4\hat{\sigma}^z_{19}\hat{\sigma}^z_{22} + 4\hat{\sigma}^z_{19}\hat{\sigma}^z_{23} + 2\hat{\sigma}^z_{19}\hat{\sigma}^z_{24} + 2\hat{\sigma}^z_{19}\hat{\sigma}^z_{25} + 2\hat{\sigma}^z_{19}\hat{\sigma}^z_{26} +$
$2\hat{\sigma}^z_{19}\hat{\sigma}^z_{27} + 2\hat{\sigma}^z_{19}\hat{\sigma}^z_{28} + 2\hat{\sigma}^z_{19}\hat{\sigma}^z_{29} + 2\hat{\sigma}^z_{19}\hat{\sigma}^z_{3} + 2\hat{\sigma}^z_{19}\hat{\sigma}^z_{30} + 180\hat{\sigma}^z_{19}\hat{\sigma}^z_{31} + 2\hat{\sigma}^z_{19}\hat{\sigma}^z_{32} + 2\hat{\sigma}^z_{19}\hat{\sigma}^z_{33} + 2\hat{\sigma}^z_{19}\hat{\sigma}^z_{34} + 2\hat{\sigma}^z_{19}\hat{\sigma}^z_{35} -$
$4\hat{\sigma}^z_{19}\hat{\sigma}^z_{36} - 4\hat{\sigma}^z_{19}\hat{\sigma}^z_{37} - 4\hat{\sigma}^z_{19}\hat{\sigma}^z_{38} - 4\hat{\sigma}^z_{19}\hat{\sigma}^z_{39} + 2\hat{\sigma}^z_{19}\hat{\sigma}^z_{4} - 4\hat{\sigma}^z_{19}\hat{\sigma}^z_{40} - 4\hat{\sigma}^z_{19}\hat{\sigma}^z_{41} - 4\hat{\sigma}^z_{19}\hat{\sigma}^z_{42} - 360\hat{\sigma}^z_{19}\hat{\sigma}^z_{43} - 4\hat{\sigma}^z_{19}\hat{\sigma}^z_{44} -$
$4\hat{\sigma}^z_{19}\hat{\sigma}^z_{45} - 4\hat{\sigma}^z_{19}\hat{\sigma}^z_{46} - 4\hat{\sigma}^z_{19}\hat{\sigma}^z_{47} + 2\hat{\sigma}^z_{19}\hat{\sigma}^z_{5} + 2\hat{\sigma}^z_{19}\hat{\sigma}^z_{6} - 4\hat{\sigma}^z_{19}\hat{\sigma}^z_{60} - 4\hat{\sigma}^z_{19}\hat{\sigma}^z_{61} - 4\hat{\sigma}^z_{19}\hat{\sigma}^z_{62} - 4\hat{\sigma}^z_{19}\hat{\sigma}^z_{63} - 4\hat{\sigma}^z_{19}\hat{\sigma}^z_{64} - 4\hat{\sigma}^z_{19}\hat{\sigma}^z_{65} -$
$4\hat{\sigma}^z_{19}\hat{\sigma}^z_{66} - 360\hat{\sigma}^z_{19}\hat{\sigma}^z_{67} - 4\hat{\sigma}^z_{19}\hat{\sigma}^z_{68} - 4\hat{\sigma}^z_{19}\hat{\sigma}^z_{69} + 180\hat{\sigma}^z_{19}\hat{\sigma}^z_{7} - 4\hat{\sigma}^z_{19}\hat{\sigma}^z_{70} - 4\hat{\sigma}^z_{19}\hat{\sigma}^z_{71} + 2\hat{\sigma}^z_{19}\hat{\sigma}^z_{8} + 2\hat{\sigma}^z_{19}\hat{\sigma}^z_{9} + 404\hat{\sigma}^z_{19} +$
$2\hat{\sigma}^z_{2}\hat{\sigma}^z_{20} + 2\hat{\sigma}^z_{2}\hat{\sigma}^z_{21} + 2\hat{\sigma}^z_{2}\hat{\sigma}^z_{22} + 2\hat{\sigma}^z_{2}\hat{\sigma}^z_{23} + 2\hat{\sigma}^z_{2}\hat{\sigma}^z_{24} + 2\hat{\sigma}^z_{2}\hat{\sigma}^z_{25} + 180\hat{\sigma}^z_{2}\hat{\sigma}^z_{26} + 2\hat{\sigma}^z_{2}\hat{\sigma}^z_{27} + 2\hat{\sigma}^z_{2}\hat{\sigma}^z_{28} + 2\hat{\sigma}^z_{2}\hat{\sigma}^z_{29} + 4\hat{\sigma}^z_{2}\hat{\sigma}^z_{3} +$
$2\hat{\sigma}^z_{2}\hat{\sigma}^z_{30} + 2\hat{\sigma}^z_{2}\hat{\sigma}^z_{31} + 2\hat{\sigma}^z_{2}\hat{\sigma}^z_{32} + 2\hat{\sigma}^z_{2}\hat{\sigma}^z_{33} + 2\hat{\sigma}^z_{2}\hat{\sigma}^z_{34} + 2\hat{\sigma}^z_{2}\hat{\sigma}^z_{35} - 4\hat{\sigma}^z_{2}\hat{\sigma}^z_{36} - 4\hat{\sigma}^z_{2}\hat{\sigma}^z_{37} - 360\hat{\sigma}^z_{2}\hat{\sigma}^z_{38} - 4\hat{\sigma}^z_{2}\hat{\sigma}^z_{39} + 4\hat{\sigma}^z_{2}\hat{\sigma}^z_{4} -$
$4\hat{\sigma}^z_{2}\hat{\sigma}^z_{40} - 4\hat{\sigma}^z_{2}\hat{\sigma}^z_{41} - 4\hat{\sigma}^z_{2}\hat{\sigma}^z_{42} - 4\hat{\sigma}^z_{2}\hat{\sigma}^z_{43} - 4\hat{\sigma}^z_{2}\hat{\sigma}^z_{44} - 4\hat{\sigma}^z_{2}\hat{\sigma}^z_{45} - 4\hat{\sigma}^z_{2}\hat{\sigma}^z_{46} - 4\hat{\sigma}^z_{2}\hat{\sigma}^z_{47} - 4\hat{\sigma}^z_{2}\hat{\sigma}^z_{48} - 4\hat{\sigma}^z_{2}\hat{\sigma}^z_{49} + 4\hat{\sigma}^z_{2}\hat{\sigma}^z_{5} -$
$360\hat{\sigma}^z_{2}\hat{\sigma}^z_{50} - 4\hat{\sigma}^z_{2}\hat{\sigma}^z_{51} - 4\hat{\sigma}^z_{2}\hat{\sigma}^z_{52} - 4\hat{\sigma}^z_{2}\hat{\sigma}^z_{53} - 4\hat{\sigma}^z_{2}\hat{\sigma}^z_{54} - 4\hat{\sigma}^z_{2}\hat{\sigma}^z_{55} - 4\hat{\sigma}^z_{2}\hat{\sigma}^z_{56} - 4\hat{\sigma}^z_{2}\hat{\sigma}^z_{57} - 4\hat{\sigma}^z_{2}\hat{\sigma}^z_{58} - 4\hat{\sigma}^z_{2}\hat{\sigma}^z_{59} + 4\hat{\sigma}^z_{2}\hat{\sigma}^z_{6} +$
$4\hat{\sigma}^z_{2}\hat{\sigma}^z_{7} + 4\hat{\sigma}^z_{2}\hat{\sigma}^z_{8} + 4\hat{\sigma}^z_{2}\hat{\sigma}^z_{9} + 404\hat{\sigma}^z_{2} + 4\hat{\sigma}^z_{20}\hat{\sigma}^z_{21} + 4\hat{\sigma}^z_{20}\hat{\sigma}^z_{22} + 4\hat{\sigma}^z_{20}\hat{\sigma}^z_{23} + 2\hat{\sigma}^z_{20}\hat{\sigma}^z_{24} + 2\hat{\sigma}^z_{20}\hat{\sigma}^z_{25} + 2\hat{\sigma}^z_{20}\hat{\sigma}^z_{26} + 2\hat{\sigma}^z_{20}\hat{\sigma}^z_{27} +$
$2\hat{\sigma}^z_{20}\hat{\sigma}^z_{28} + 2\hat{\sigma}^z_{20}\hat{\sigma}^z_{29} + 2\hat{\sigma}^z_{20}\hat{\sigma}^z_{3} + 2\hat{\sigma}^z_{20}\hat{\sigma}^z_{30} + 2\hat{\sigma}^z_{20}\hat{\sigma}^z_{31} + 180\hat{\sigma}^z_{20}\hat{\sigma}^z_{32} + 2\hat{\sigma}^z_{20}\hat{\sigma}^z_{33} + 2\hat{\sigma}^z_{20}\hat{\sigma}^z_{34} + 2\hat{\sigma}^z_{20}\hat{\sigma}^z_{35} - 4\hat{\sigma}^z_{20}\hat{\sigma}^z_{36} -$
$4\hat{\sigma}^z_{20}\hat{\sigma}^z_{37} - 4\hat{\sigma}^z_{20}\hat{\sigma}^z_{38} - 4\hat{\sigma}^z_{20}\hat{\sigma}^z_{39} + 2\hat{\sigma}^z_{20}\hat{\sigma}^z_{4} - 4\hat{\sigma}^z_{20}\hat{\sigma}^z_{40} - 4\hat{\sigma}^z_{20}\hat{\sigma}^z_{41} - 4\hat{\sigma}^z_{20}\hat{\sigma}^z_{42} - 4\hat{\sigma}^z_{20}\hat{\sigma}^z_{43} - 360\hat{\sigma}^z_{20}\hat{\sigma}^z_{44} - 4\hat{\sigma}^z_{20}\hat{\sigma}^z_{45} -$
$4\hat{\sigma}^z_{20}\hat{\sigma}^z_{46} - 4\hat{\sigma}^z_{20}\hat{\sigma}^z_{47} + 2\hat{\sigma}^z_{20}\hat{\sigma}^z_{5} + 2\hat{\sigma}^z_{20}\hat{\sigma}^z_{6} - 4\hat{\sigma}^z_{20}\hat{\sigma}^z_{60} - 4\hat{\sigma}^z_{20}\hat{\sigma}^z_{61} - 4\hat{\sigma}^z_{20}\hat{\sigma}^z_{62} - 4\hat{\sigma}^z_{20}\hat{\sigma}^z_{63} - 4\hat{\sigma}^z_{20}\hat{\sigma}^z_{64} - 4\hat{\sigma}^z_{20}\hat{\sigma}^z_{65} - 4\hat{\sigma}^z_{20}\hat{\sigma}^z_{66} -$
$4\hat{\sigma}^z_{20}\hat{\sigma}^z_{67} - 360\hat{\sigma}^z_{20}\hat{\sigma}^z_{68} - 4\hat{\sigma}^z_{20}\hat{\sigma}^z_{69} + 2\hat{\sigma}^z_{20}\hat{\sigma}^z_{7} - 4\hat{\sigma}^z_{20}\hat{\sigma}^z_{70} - 4\hat{\sigma}^z_{20}\hat{\sigma}^z_{71} + 180\hat{\sigma}^z_{20}\hat{\sigma}^z_{8} + 2\hat{\sigma}^z_{20}\hat{\sigma}^z_{9} + 404\hat{\sigma}^z_{20} + 4\hat{\sigma}^z_{21}\hat{\sigma}^z_{22} +$
$4\hat{\sigma}^z_{21}\hat{\sigma}^z_{23} + 2\hat{\sigma}^z_{21}\hat{\sigma}^z_{24} + 2\hat{\sigma}^z_{21}\hat{\sigma}^z_{25} + 2\hat{\sigma}^z_{21}\hat{\sigma}^z_{26} + 2\hat{\sigma}^z_{21}\hat{\sigma}^z_{27} + 2\hat{\sigma}^z_{21}\hat{\sigma}^z_{28} + 2\hat{\sigma}^z_{21}\hat{\sigma}^z_{29} + 2\hat{\sigma}^z_{21}\hat{\sigma}^z_{3} + 2\hat{\sigma}^z_{21}\hat{\sigma}^z_{30} + 2\hat{\sigma}^z_{21}\hat{\sigma}^z_{31} + 2\hat{\sigma}^z_{21}\hat{\sigma}^z_{32} +$
$180\hat{\sigma}^z_{21}\hat{\sigma}^z_{33} + 2\hat{\sigma}^z_{21}\hat{\sigma}^z_{34} + 2\hat{\sigma}^z_{21}\hat{\sigma}^z_{35} - 4\hat{\sigma}^z_{21}\hat{\sigma}^z_{36} - 4\hat{\sigma}^z_{21}\hat{\sigma}^z_{37} - 4\hat{\sigma}^z_{21}\hat{\sigma}^z_{38} - 4\hat{\sigma}^z_{21}\hat{\sigma}^z_{39} + 2\hat{\sigma}^z_{21}\hat{\sigma}^z_{4} - 4\hat{\sigma}^z_{21}\hat{\sigma}^z_{40} - 4\hat{\sigma}^z_{21}\hat{\sigma}^z_{41} -$
$4\hat{\sigma}^z_{21}\hat{\sigma}^z_{42} - 4\hat{\sigma}^z_{21}\hat{\sigma}^z_{43} - 4\hat{\sigma}^z_{21}\hat{\sigma}^z_{44} - 360\hat{\sigma}^z_{21}\hat{\sigma}^z_{45} - 4\hat{\sigma}^z_{21}\hat{\sigma}^z_{46} - 4\hat{\sigma}^z_{21}\hat{\sigma}^z_{47} + 2\hat{\sigma}^z_{21}\hat{\sigma}^z_{5} + 2\hat{\sigma}^z_{21}\hat{\sigma}^z_{6} - 4\hat{\sigma}^z_{21}\hat{\sigma}^z_{60} - 4\hat{\sigma}^z_{21}\hat{\sigma}^z_{61} -$
$4\hat{\sigma}^z_{21}\hat{\sigma}^z_{62} - 4\hat{\sigma}^z_{21}\hat{\sigma}^z_{63} - 4\hat{\sigma}^z_{21}\hat{\sigma}^z_{64} - 4\hat{\sigma}^z_{21}\hat{\sigma}^z_{65} - 4\hat{\sigma}^z_{21}\hat{\sigma}^z_{66} - 4\hat{\sigma}^z_{21}\hat{\sigma}^z_{67} - 4\hat{\sigma}^z_{21}\hat{\sigma}^z_{68} - 360\hat{\sigma}^z_{21}\hat{\sigma}^z_{69} + 2\hat{\sigma}^z_{21}\hat{\sigma}^z_{7} - 4\hat{\sigma}^z_{21}\hat{\sigma}^z_{70} -$
$4\hat{\sigma}^z_{21}\hat{\sigma}^z_{71} + 2\hat{\sigma}^z_{21}\hat{\sigma}^z_{8} + 180\hat{\sigma}^z_{21}\hat{\sigma}^z_{9} + 404\hat{\sigma}^z_{21} + 4\hat{\sigma}^z_{22}\hat{\sigma}^z_{23} + 2\hat{\sigma}^z_{22}\hat{\sigma}^z_{24} + 2\hat{\sigma}^z_{22}\hat{\sigma}^z_{25} + 2\hat{\sigma}^z_{22}\hat{\sigma}^z_{26} + 2\hat{\sigma}^z_{22}\hat{\sigma}^z_{27} + 2\hat{\sigma}^z_{22}\hat{\sigma}^z_{28} + 2\hat{\sigma}^z_{22}\hat{\sigma}^z_{29} +$
$2\hat{\sigma}^z_{22}\hat{\sigma}^z_{3} + 2\hat{\sigma}^z_{22}\hat{\sigma}^z_{30} + 2\hat{\sigma}^z_{22}\hat{\sigma}^z_{31} + 2\hat{\sigma}^z_{22}\hat{\sigma}^z_{32} + 2\hat{\sigma}^z_{22}\hat{\sigma}^z_{33} + 180\hat{\sigma}^z_{22}\hat{\sigma}^z_{34} + 2\hat{\sigma}^z_{22}\hat{\sigma}^z_{35} - 4\hat{\sigma}^z_{22}\hat{\sigma}^z_{36} - 4\hat{\sigma}^z_{22}\hat{\sigma}^z_{37} - 4\hat{\sigma}^z_{22}\hat{\sigma}^z_{38} -$
$4\hat{\sigma}^z_{22}\hat{\sigma}^z_{39} + 2\hat{\sigma}^z_{22}\hat{\sigma}^z_{4} - 4\hat{\sigma}^z_{22}\hat{\sigma}^z_{40} - 4\hat{\sigma}^z_{22}\hat{\sigma}^z_{41} - 4\hat{\sigma}^z_{22}\hat{\sigma}^z_{42} - 4\hat{\sigma}^z_{22}\hat{\sigma}^z_{43} - 4\hat{\sigma}^z_{22}\hat{\sigma}^z_{44} - 4\hat{\sigma}^z_{22}\hat{\sigma}^z_{45} - 360\hat{\sigma}^z_{22}\hat{\sigma}^z_{46} - 4\hat{\sigma}^z_{22}\hat{\sigma}^z_{47} +$
$2\hat{\sigma}^z_{22}\hat{\sigma}^z_{5} + 2\hat{\sigma}^z_{22}\hat{\sigma}^z_{6} - 4\hat{\sigma}^z_{22}\hat{\sigma}^z_{60} - 4\hat{\sigma}^z_{22}\hat{\sigma}^z_{61} - 4\hat{\sigma}^z_{22}\hat{\sigma}^z_{62} - 4\hat{\sigma}^z_{22}\hat{\sigma}^z_{63} - 4\hat{\sigma}^z_{22}\hat{\sigma}^z_{64} - 4\hat{\sigma}^z_{22}\hat{\sigma}^z_{65} - 4\hat{\sigma}^z_{22}\hat{\sigma}^z_{66} - 4\hat{\sigma}^z_{22}\hat{\sigma}^z_{67} - 4\hat{\sigma}^z_{22}\hat{\sigma}^z_{68} -$
$4\hat{\sigma}^z_{22}\hat{\sigma}^z_{69} + 2\hat{\sigma}^z_{22}\hat{\sigma}^z_{7} - 360\hat{\sigma}^z_{22}\hat{\sigma}^z_{70} - 4\hat{\sigma}^z_{22}\hat{\sigma}^z_{71} + 2\hat{\sigma}^z_{22}\hat{\sigma}^z_{8} + 2\hat{\sigma}^z_{22}\hat{\sigma}^z_{9} + 404\hat{\sigma}^z_{22} + 2\hat{\sigma}^z_{23}\hat{\sigma}^z_{24} + 2\hat{\sigma}^z_{23}\hat{\sigma}^z_{25} + 2\hat{\sigma}^z_{23}\hat{\sigma}^z_{26} + 2\hat{\sigma}^z_{23}\hat{\sigma}^z_{27} +$
$2\hat{\sigma}^z_{23}\hat{\sigma}^z_{28} + 2\hat{\sigma}^z_{23}\hat{\sigma}^z_{29} + 2\hat{\sigma}^z_{23}\hat{\sigma}^z_{3} + 2\hat{\sigma}^z_{23}\hat{\sigma}^z_{30} + 2\hat{\sigma}^z_{23}\hat{\sigma}^z_{31} + 2\hat{\sigma}^z_{23}\hat{\sigma}^z_{32} + 2\hat{\sigma}^z_{23}\hat{\sigma}^z_{33} + 2\hat{\sigma}^z_{23}\hat{\sigma}^z_{34} + 180\hat{\sigma}^z_{23}\hat{\sigma}^z_{35} - 4\hat{\sigma}^z_{23}\hat{\sigma}^z_{36} -$
$4\hat{\sigma}^z_{23}\hat{\sigma}^z_{37} - 4\hat{\sigma}^z_{23}\hat{\sigma}^z_{38} - 4\hat{\sigma}^z_{23}\hat{\sigma}^z_{39} + 2\hat{\sigma}^z_{23}\hat{\sigma}^z_{4} - 4\hat{\sigma}^z_{23}\hat{\sigma}^z_{40} - 4\hat{\sigma}^z_{23}\hat{\sigma}^z_{41} - 4\hat{\sigma}^z_{23}\hat{\sigma}^z_{42} - 4\hat{\sigma}^z_{23}\hat{\sigma}^z_{43} - 4\hat{\sigma}^z_{23}\hat{\sigma}^z_{44} - 4\hat{\sigma}^z_{23}\hat{\sigma}^z_{45} - 4\hat{\sigma}^z_{23}\hat{\sigma}^z_{46} -$
$360\hat{\sigma}^z_{23}\hat{\sigma}^z_{47} + 2\hat{\sigma}^z_{23}\hat{\sigma}^z_{5} + 2\hat{\sigma}^z_{23}\hat{\sigma}^z_{6} - 4\hat{\sigma}^z_{23}\hat{\sigma}^z_{60} - 4\hat{\sigma}^z_{23}\hat{\sigma}^z_{61} - 4\hat{\sigma}^z_{23}\hat{\sigma}^z_{62} - 4\hat{\sigma}^z_{23}\hat{\sigma}^z_{63} - 4\hat{\sigma}^z_{23}\hat{\sigma}^z_{64} - 4\hat{\sigma}^z_{23}\hat{\sigma}^z_{65} - 4\hat{\sigma}^z_{23}\hat{\sigma}^z_{66} -$
$4\hat{\sigma}^z_{23}\hat{\sigma}^z_{67} - 4\hat{\sigma}^z_{23}\hat{\sigma}^z_{68} - 4\hat{\sigma}^z_{23}\hat{\sigma}^z_{69} + 2\hat{\sigma}^z_{23}\hat{\sigma}^z_{7} - 4\hat{\sigma}^z_{23}\hat{\sigma}^z_{70} - 360\hat{\sigma}^z_{23}\hat{\sigma}^z_{71} + 2\hat{\sigma}^z_{23}\hat{\sigma}^z_{8} + 2\hat{\sigma}^z_{23}\hat{\sigma}^z_{9} + 404\hat{\sigma}^z_{23} + 4\hat{\sigma}^z_{24}\hat{\sigma}^z_{25} + 4\hat{\sigma}^z_{24}\hat{\sigma}^z_{26} +$
$4\hat{\sigma}^z_{24}\hat{\sigma}^z_{27} + 4\hat{\sigma}^z_{24}\hat{\sigma}^z_{28} + 4\hat{\sigma}^z_{24}\hat{\sigma}^z_{29} + 2\hat{\sigma}^z_{24}\hat{\sigma}^z_{3} + 4\hat{\sigma}^z_{24}\hat{\sigma}^z_{30} + 4\hat{\sigma}^z_{24}\hat{\sigma}^z_{31} + 4\hat{\sigma}^z_{24}\hat{\sigma}^z_{32} + 4\hat{\sigma}^z_{24}\hat{\sigma}^z_{33} + 4\hat{\sigma}^z_{24}\hat{\sigma}^z_{34} + 4\hat{\sigma}^z_{24}\hat{\sigma}^z_{35} + 2\hat{\sigma}^z_{24}\hat{\sigma}^z_{4} -$
$360\hat{\sigma}^z_{24}\hat{\sigma}^z_{48} - 4\hat{\sigma}^z_{24}\hat{\sigma}^z_{49} + 2\hat{\sigma}^z_{24}\hat{\sigma}^z_{5} - 4\hat{\sigma}^z_{24}\hat{\sigma}^z_{50} - 4\hat{\sigma}^z_{24}\hat{\sigma}^z_{51} - 4\hat{\sigma}^z_{24}\hat{\sigma}^z_{52} - 4\hat{\sigma}^z_{24}\hat{\sigma}^z_{53} - 4\hat{\sigma}^z_{24}\hat{\sigma}^z_{54} - 4\hat{\sigma}^z_{24}\hat{\sigma}^z_{55} - 4\hat{\sigma}^z_{24}\hat{\sigma}^z_{56} -$
$4\hat{\sigma}^z_{24}\hat{\sigma}^z_{57} - 4\hat{\sigma}^z_{24}\hat{\sigma}^z_{58} - 4\hat{\sigma}^z_{24}\hat{\sigma}^z_{59} + 2\hat{\sigma}^z_{24}\hat{\sigma}^z_{6} - 360\hat{\sigma}^z_{24}\hat{\sigma}^z_{60} - 4\hat{\sigma}^z_{24}\hat{\sigma}^z_{61} - 4\hat{\sigma}^z_{24}\hat{\sigma}^z_{62} - 4\hat{\sigma}^z_{24}\hat{\sigma}^z_{63} - 4\hat{\sigma}^z_{24}\hat{\sigma}^z_{64} - 4\hat{\sigma}^z_{24}\hat{\sigma}^z_{65} -$
$4\hat{\sigma}^z_{24}\hat{\sigma}^z_{66} - 4\hat{\sigma}^z_{24}\hat{\sigma}^z_{67} - 4\hat{\sigma}^z_{24}\hat{\sigma}^z_{68} - 4\hat{\sigma}^z_{24}\hat{\sigma}^z_{69} + 2\hat{\sigma}^z_{24}\hat{\sigma}^z_{7} - 4\hat{\sigma}^z_{24}\hat{\sigma}^z_{70} - 4\hat{\sigma}^z_{24}\hat{\sigma}^z_{71} + 2\hat{\sigma}^z_{24}\hat{\sigma}^z_{8} + 2\hat{\sigma}^z_{24}\hat{\sigma}^z_{9} + 404\hat{\sigma}^z_{24} + 4\hat{\sigma}^z_{25}\hat{\sigma}^z_{26} +$
$4\hat{\sigma}^z_{25}\hat{\sigma}^z_{27} + 4\hat{\sigma}^z_{25}\hat{\sigma}^z_{28} + 4\hat{\sigma}^z_{25}\hat{\sigma}^z_{29} + 2\hat{\sigma}^z_{25}\hat{\sigma}^z_{3} + 4\hat{\sigma}^z_{25}\hat{\sigma}^z_{30} + 4\hat{\sigma}^z_{25}\hat{\sigma}^z_{31} + 4\hat{\sigma}^z_{25}\hat{\sigma}^z_{32} + 4\hat{\sigma}^z_{25}\hat{\sigma}^z_{33} + 4\hat{\sigma}^z_{25}\hat{\sigma}^z_{34} + 4\hat{\sigma}^z_{25}\hat{\sigma}^z_{35} + 2\hat{\sigma}^z_{25}\hat{\sigma}^z_{4} -$
$4\hat{\sigma}^z_{25}\hat{\sigma}^z_{48} - 360\hat{\sigma}^z_{25}\hat{\sigma}^z_{49} + 2\hat{\sigma}^z_{25}\hat{\sigma}^z_{5} - 4\hat{\sigma}^z_{25}\hat{\sigma}^z_{50} - 4\hat{\sigma}^z_{25}\hat{\sigma}^z_{51} - 4\hat{\sigma}^z_{25}\hat{\sigma}^z_{52} - 4\hat{\sigma}^z_{25}\hat{\sigma}^z_{53} - 4\hat{\sigma}^z_{25}\hat{\sigma}^z_{54} - 4\hat{\sigma}^z_{25}\hat{\sigma}^z_{55} - 4\hat{\sigma}^z_{25}\hat{\sigma}^z_{56} -$
$4\hat{\sigma}^z_{25}\hat{\sigma}^z_{57} - 4\hat{\sigma}^z_{25}\hat{\sigma}^z_{58} - 4\hat{\sigma}^z_{25}\hat{\sigma}^z_{59} + 2\hat{\sigma}^z_{25}\hat{\sigma}^z_{6} - 4\hat{\sigma}^z_{25}\hat{\sigma}^z_{60} - 360\hat{\sigma}^z_{25}\hat{\sigma}^z_{61} - 4\hat{\sigma}^z_{25}\hat{\sigma}^z_{62} - 4\hat{\sigma}^z_{25}\hat{\sigma}^z_{63} - 4\hat{\sigma}^z_{25}\hat{\sigma}^z_{64} - 4\hat{\sigma}^z_{25}\hat{\sigma}^z_{65} -$
$4\hat{\sigma}^z_{25}\hat{\sigma}^z_{66} - 4\hat{\sigma}^z_{25}\hat{\sigma}^z_{67} - 4\hat{\sigma}^z_{25}\hat{\sigma}^z_{68} - 4\hat{\sigma}^z_{25}\hat{\sigma}^z_{69} + 2\hat{\sigma}^z_{25}\hat{\sigma}^z_{7} - 4\hat{\sigma}^z_{25}\hat{\sigma}^z_{70} - 4\hat{\sigma}^z_{25}\hat{\sigma}^z_{71} + 2\hat{\sigma}^z_{25}\hat{\sigma}^z_{8} + 2\hat{\sigma}^z_{25}\hat{\sigma}^z_{9} + 404\hat{\sigma}^z_{25} + 4\hat{\sigma}^z_{26}\hat{\sigma}^z_{27} +$
$4\hat{\sigma}^z_{26}\hat{\sigma}^z_{28} + 4\hat{\sigma}^z_{26}\hat{\sigma}^z_{29} + 2\hat{\sigma}^z_{26}\hat{\sigma}^z_{3} + 4\hat{\sigma}^z_{26}\hat{\sigma}^z_{30} + 4\hat{\sigma}^z_{26}\hat{\sigma}^z_{31} + 4\hat{\sigma}^z_{26}\hat{\sigma}^z_{32} + 4\hat{\sigma}^z_{26}\hat{\sigma}^z_{33} + 4\hat{\sigma}^z_{26}\hat{\sigma}^z_{34} + 4\hat{\sigma}^z_{26}\hat{\sigma}^z_{35} + 2\hat{\sigma}^z_{26}\hat{\sigma}^z_{4} - 4\hat{\sigma}^z_{26}\hat{\sigma}^z_{48} -$

$$4\hat{\sigma}^z_{26}\hat{\sigma}^z_{49} + 2\hat{\sigma}^z_{26}\hat{\sigma}^z_{5} - 360\hat{\sigma}^z_{26}\hat{\sigma}^z_{50} - 4\hat{\sigma}^z_{26}\hat{\sigma}^z_{51} - 4\hat{\sigma}^z_{26}\hat{\sigma}^z_{52} - 4\hat{\sigma}^z_{26}\hat{\sigma}^z_{53} - 4\hat{\sigma}^z_{26}\hat{\sigma}^z_{54} - 4\hat{\sigma}^z_{26}\hat{\sigma}^z_{55} - 4\hat{\sigma}^z_{26}\hat{\sigma}^z_{56} - 4\hat{\sigma}^z_{26}\hat{\sigma}^z_{57} -$$
$$4\hat{\sigma}^z_{26}\hat{\sigma}^z_{58} - 4\hat{\sigma}^z_{26}\hat{\sigma}^z_{59} + 2\hat{\sigma}^z_{26}\hat{\sigma}^z_{6} - 4\hat{\sigma}^z_{26}\hat{\sigma}^z_{60} - 4\hat{\sigma}^z_{26}\hat{\sigma}^z_{61} - 360\hat{\sigma}^z_{26}\hat{\sigma}^z_{62} - 4\hat{\sigma}^z_{26}\hat{\sigma}^z_{63} - 4\hat{\sigma}^z_{26}\hat{\sigma}^z_{64} - 4\hat{\sigma}^z_{26}\hat{\sigma}^z_{65} - 4\hat{\sigma}^z_{26}\hat{\sigma}^z_{66} -$$
$$4\hat{\sigma}^z_{26}\hat{\sigma}^z_{67} - 4\hat{\sigma}^z_{26}\hat{\sigma}^z_{68} - 4\hat{\sigma}^z_{26}\hat{\sigma}^z_{69} + 2\hat{\sigma}^z_{26}\hat{\sigma}^z_{7} - 4\hat{\sigma}^z_{26}\hat{\sigma}^z_{70} - 4\hat{\sigma}^z_{26}\hat{\sigma}^z_{71} + 2\hat{\sigma}^z_{26}\hat{\sigma}^z_{8} + 2\hat{\sigma}^z_{26}\hat{\sigma}^z_{9} + 404\hat{\sigma}^z_{26} + 4\hat{\sigma}^z_{27}\hat{\sigma}^z_{28} + 4\hat{\sigma}^z_{27}\hat{\sigma}^z_{29} +$$
$$180\hat{\sigma}^z_{27}\hat{\sigma}^z_{3} + 4\hat{\sigma}^z_{27}\hat{\sigma}^z_{30} + 4\hat{\sigma}^z_{27}\hat{\sigma}^z_{31} + 4\hat{\sigma}^z_{27}\hat{\sigma}^z_{32} + 4\hat{\sigma}^z_{27}\hat{\sigma}^z_{33} + 4\hat{\sigma}^z_{27}\hat{\sigma}^z_{34} + 4\hat{\sigma}^z_{27}\hat{\sigma}^z_{35} + 2\hat{\sigma}^z_{27}\hat{\sigma}^z_{4} - 4\hat{\sigma}^z_{27}\hat{\sigma}^z_{48} - 4\hat{\sigma}^z_{27}\hat{\sigma}^z_{49} +$$
$$2\hat{\sigma}^z_{27}\hat{\sigma}^z_{5} - 4\hat{\sigma}^z_{27}\hat{\sigma}^z_{50} - 360\hat{\sigma}^z_{27}\hat{\sigma}^z_{51} - 4\hat{\sigma}^z_{27}\hat{\sigma}^z_{52} - 4\hat{\sigma}^z_{27}\hat{\sigma}^z_{53} - 4\hat{\sigma}^z_{27}\hat{\sigma}^z_{54} - 4\hat{\sigma}^z_{27}\hat{\sigma}^z_{55} - 4\hat{\sigma}^z_{27}\hat{\sigma}^z_{56} - 4\hat{\sigma}^z_{27}\hat{\sigma}^z_{57} - 4\hat{\sigma}^z_{27}\hat{\sigma}^z_{58} -$$
$$4\hat{\sigma}^z_{27}\hat{\sigma}^z_{59} + 2\hat{\sigma}^z_{27}\hat{\sigma}^z_{6} - 4\hat{\sigma}^z_{27}\hat{\sigma}^z_{60} - 4\hat{\sigma}^z_{27}\hat{\sigma}^z_{61} - 4\hat{\sigma}^z_{27}\hat{\sigma}^z_{62} - 360\hat{\sigma}^z_{27}\hat{\sigma}^z_{63} - 4\hat{\sigma}^z_{27}\hat{\sigma}^z_{64} - 4\hat{\sigma}^z_{27}\hat{\sigma}^z_{65} - 4\hat{\sigma}^z_{27}\hat{\sigma}^z_{66} - 4\hat{\sigma}^z_{27}\hat{\sigma}^z_{67} -$$
$$4\hat{\sigma}^z_{27}\hat{\sigma}^z_{68} - 4\hat{\sigma}^z_{27}\hat{\sigma}^z_{69} + 2\hat{\sigma}^z_{27}\hat{\sigma}^z_{7} - 4\hat{\sigma}^z_{27}\hat{\sigma}^z_{70} - 4\hat{\sigma}^z_{27}\hat{\sigma}^z_{71} + 2\hat{\sigma}^z_{27}\hat{\sigma}^z_{8} + 2\hat{\sigma}^z_{27}\hat{\sigma}^z_{9} + 404\hat{\sigma}^z_{27} + 4\hat{\sigma}^z_{28}\hat{\sigma}^z_{29} + 2\hat{\sigma}^z_{28}\hat{\sigma}^z_{3} + 4\hat{\sigma}^z_{28}\hat{\sigma}^z_{30} +$$
$$4\hat{\sigma}^z_{28}\hat{\sigma}^z_{31} + 4\hat{\sigma}^z_{28}\hat{\sigma}^z_{32} + 4\hat{\sigma}^z_{28}\hat{\sigma}^z_{33} + 4\hat{\sigma}^z_{28}\hat{\sigma}^z_{34} + 4\hat{\sigma}^z_{28}\hat{\sigma}^z_{35} + 180\hat{\sigma}^z_{28}\hat{\sigma}^z_{4} - 4\hat{\sigma}^z_{28}\hat{\sigma}^z_{48} - 4\hat{\sigma}^z_{28}\hat{\sigma}^z_{49} + 2\hat{\sigma}^z_{28}\hat{\sigma}^z_{5} - 4\hat{\sigma}^z_{28}\hat{\sigma}^z_{50} -$$
$$4\hat{\sigma}^z_{28}\hat{\sigma}^z_{51} - 360\hat{\sigma}^z_{28}\hat{\sigma}^z_{52} - 4\hat{\sigma}^z_{28}\hat{\sigma}^z_{53} - 4\hat{\sigma}^z_{28}\hat{\sigma}^z_{54} - 4\hat{\sigma}^z_{28}\hat{\sigma}^z_{55} - 4\hat{\sigma}^z_{28}\hat{\sigma}^z_{56} - 4\hat{\sigma}^z_{28}\hat{\sigma}^z_{57} - 4\hat{\sigma}^z_{28}\hat{\sigma}^z_{58} - 4\hat{\sigma}^z_{28}\hat{\sigma}^z_{59} + 2\hat{\sigma}^z_{28}\hat{\sigma}^z_{6} -$$
$$4\hat{\sigma}^z_{28}\hat{\sigma}^z_{60} - 4\hat{\sigma}^z_{28}\hat{\sigma}^z_{61} - 4\hat{\sigma}^z_{28}\hat{\sigma}^z_{62} - 4\hat{\sigma}^z_{28}\hat{\sigma}^z_{63} - 360\hat{\sigma}^z_{28}\hat{\sigma}^z_{64} - 4\hat{\sigma}^z_{28}\hat{\sigma}^z_{65} - 4\hat{\sigma}^z_{28}\hat{\sigma}^z_{66} - 4\hat{\sigma}^z_{28}\hat{\sigma}^z_{67} - 4\hat{\sigma}^z_{28}\hat{\sigma}^z_{68} - 4\hat{\sigma}^z_{28}\hat{\sigma}^z_{69} +$$
$$2\hat{\sigma}^z_{28}\hat{\sigma}^z_{7} - 4\hat{\sigma}^z_{28}\hat{\sigma}^z_{70} - 4\hat{\sigma}^z_{28}\hat{\sigma}^z_{71} + 2\hat{\sigma}^z_{28}\hat{\sigma}^z_{8} + 2\hat{\sigma}^z_{28}\hat{\sigma}^z_{9} + 404\hat{\sigma}^z_{28} + 2\hat{\sigma}^z_{29}\hat{\sigma}^z_{3} + 4\hat{\sigma}^z_{29}\hat{\sigma}^z_{30} + 4\hat{\sigma}^z_{29}\hat{\sigma}^z_{31} + 4\hat{\sigma}^z_{29}\hat{\sigma}^z_{32} + 4\hat{\sigma}^z_{29}\hat{\sigma}^z_{33} +$$
$$4\hat{\sigma}^z_{29}\hat{\sigma}^z_{34} + 4\hat{\sigma}^z_{29}\hat{\sigma}^z_{35} + 2\hat{\sigma}^z_{29}\hat{\sigma}^z_{4} - 4\hat{\sigma}^z_{29}\hat{\sigma}^z_{48} - 4\hat{\sigma}^z_{29}\hat{\sigma}^z_{49} + 180\hat{\sigma}^z_{29}\hat{\sigma}^z_{5} - 4\hat{\sigma}^z_{29}\hat{\sigma}^z_{50} - 4\hat{\sigma}^z_{29}\hat{\sigma}^z_{51} - 4\hat{\sigma}^z_{29}\hat{\sigma}^z_{52} - 360\hat{\sigma}^z_{29}\hat{\sigma}^z_{53} -$$
$$4\hat{\sigma}^z_{29}\hat{\sigma}^z_{54} - 4\hat{\sigma}^z_{29}\hat{\sigma}^z_{55} - 4\hat{\sigma}^z_{29}\hat{\sigma}^z_{56} - 4\hat{\sigma}^z_{29}\hat{\sigma}^z_{57} - 4\hat{\sigma}^z_{29}\hat{\sigma}^z_{58} - 4\hat{\sigma}^z_{29}\hat{\sigma}^z_{59} + 2\hat{\sigma}^z_{29}\hat{\sigma}^z_{6} - 4\hat{\sigma}^z_{29}\hat{\sigma}^z_{60} - 4\hat{\sigma}^z_{29}\hat{\sigma}^z_{61} - 4\hat{\sigma}^z_{29}\hat{\sigma}^z_{62} - 4\hat{\sigma}^z_{29}\hat{\sigma}^z_{63} -$$
$$4\hat{\sigma}^z_{29}\hat{\sigma}^z_{64} - 360\hat{\sigma}^z_{29}\hat{\sigma}^z_{65} - 4\hat{\sigma}^z_{29}\hat{\sigma}^z_{66} - 4\hat{\sigma}^z_{29}\hat{\sigma}^z_{67} - 4\hat{\sigma}^z_{29}\hat{\sigma}^z_{68} - 4\hat{\sigma}^z_{29}\hat{\sigma}^z_{69} + 2\hat{\sigma}^z_{29}\hat{\sigma}^z_{7} - 4\hat{\sigma}^z_{29}\hat{\sigma}^z_{70} - 4\hat{\sigma}^z_{29}\hat{\sigma}^z_{71} + 2\hat{\sigma}^z_{29}\hat{\sigma}^z_{8} +$$
$$2\hat{\sigma}^z_{29}\hat{\sigma}^z_{9} + 404\hat{\sigma}^z_{29} + 2\hat{\sigma}^z_{3}\hat{\sigma}^z_{30} + 2\hat{\sigma}^z_{3}\hat{\sigma}^z_{31} + 2\hat{\sigma}^z_{3}\hat{\sigma}^z_{32} + 2\hat{\sigma}^z_{3}\hat{\sigma}^z_{33} + 2\hat{\sigma}^z_{3}\hat{\sigma}^z_{34} + 2\hat{\sigma}^z_{3}\hat{\sigma}^z_{35} - 4\hat{\sigma}^z_{3}\hat{\sigma}^z_{36} - 4\hat{\sigma}^z_{3}\hat{\sigma}^z_{37} - 4\hat{\sigma}^z_{3}\hat{\sigma}^z_{38} -$$
$$360\hat{\sigma}^z_{3}\hat{\sigma}^z_{39} + 4\hat{\sigma}^z_{3}\hat{\sigma}^z_{4} - 4\hat{\sigma}^z_{3}\hat{\sigma}^z_{40} - 4\hat{\sigma}^z_{3}\hat{\sigma}^z_{41} - 4\hat{\sigma}^z_{3}\hat{\sigma}^z_{42} - 4\hat{\sigma}^z_{3}\hat{\sigma}^z_{43} - 4\hat{\sigma}^z_{3}\hat{\sigma}^z_{44} - 4\hat{\sigma}^z_{3}\hat{\sigma}^z_{45} - 4\hat{\sigma}^z_{3}\hat{\sigma}^z_{46} - 4\hat{\sigma}^z_{3}\hat{\sigma}^z_{47} - 4\hat{\sigma}^z_{3}\hat{\sigma}^z_{48} -$$
$$4\hat{\sigma}^z_{3}\hat{\sigma}^z_{49} + 4\hat{\sigma}^z_{3}\hat{\sigma}^z_{5} - 4\hat{\sigma}^z_{3}\hat{\sigma}^z_{50} - 360\hat{\sigma}^z_{3}\hat{\sigma}^z_{51} - 4\hat{\sigma}^z_{3}\hat{\sigma}^z_{52} - 4\hat{\sigma}^z_{3}\hat{\sigma}^z_{53} - 4\hat{\sigma}^z_{3}\hat{\sigma}^z_{54} - 4\hat{\sigma}^z_{3}\hat{\sigma}^z_{55} - 4\hat{\sigma}^z_{3}\hat{\sigma}^z_{56} - 4\hat{\sigma}^z_{3}\hat{\sigma}^z_{57} - 4\hat{\sigma}^z_{3}\hat{\sigma}^z_{58} -$$
$$4\hat{\sigma}^z_{3}\hat{\sigma}^z_{59} + 4\hat{\sigma}^z_{3}\hat{\sigma}^z_{6} + 4\hat{\sigma}^z_{3}\hat{\sigma}^z_{7} + 4\hat{\sigma}^z_{3}\hat{\sigma}^z_{8} + 4\hat{\sigma}^z_{3}\hat{\sigma}^z_{9} + 404\hat{\sigma}^z_{3} + 4\hat{\sigma}^z_{30}\hat{\sigma}^z_{31} + 4\hat{\sigma}^z_{30}\hat{\sigma}^z_{32} + 4\hat{\sigma}^z_{30}\hat{\sigma}^z_{33} + 4\hat{\sigma}^z_{30}\hat{\sigma}^z_{34} + 4\hat{\sigma}^z_{30}\hat{\sigma}^z_{35} +$$
$$2\hat{\sigma}^z_{30}\hat{\sigma}^z_{4} - 4\hat{\sigma}^z_{30}\hat{\sigma}^z_{48} - 4\hat{\sigma}^z_{30}\hat{\sigma}^z_{49} + 2\hat{\sigma}^z_{30}\hat{\sigma}^z_{5} - 4\hat{\sigma}^z_{30}\hat{\sigma}^z_{50} - 4\hat{\sigma}^z_{30}\hat{\sigma}^z_{51} - 4\hat{\sigma}^z_{30}\hat{\sigma}^z_{52} - 4\hat{\sigma}^z_{30}\hat{\sigma}^z_{53} - 360\hat{\sigma}^z_{30}\hat{\sigma}^z_{54} - 4\hat{\sigma}^z_{30}\hat{\sigma}^z_{55} -$$
$$4\hat{\sigma}^z_{30}\hat{\sigma}^z_{56} - 4\hat{\sigma}^z_{30}\hat{\sigma}^z_{57} - 4\hat{\sigma}^z_{30}\hat{\sigma}^z_{58} - 4\hat{\sigma}^z_{30}\hat{\sigma}^z_{59} + 180\hat{\sigma}^z_{30}\hat{\sigma}^z_{6} - 4\hat{\sigma}^z_{30}\hat{\sigma}^z_{60} - 4\hat{\sigma}^z_{30}\hat{\sigma}^z_{61} - 4\hat{\sigma}^z_{30}\hat{\sigma}^z_{62} - 4\hat{\sigma}^z_{30}\hat{\sigma}^z_{63} - 4\hat{\sigma}^z_{30}\hat{\sigma}^z_{64} -$$
$$4\hat{\sigma}^z_{30}\hat{\sigma}^z_{65} - 360\hat{\sigma}^z_{30}\hat{\sigma}^z_{66} - 4\hat{\sigma}^z_{30}\hat{\sigma}^z_{67} - 4\hat{\sigma}^z_{30}\hat{\sigma}^z_{68} - 4\hat{\sigma}^z_{30}\hat{\sigma}^z_{69} + 2\hat{\sigma}^z_{30}\hat{\sigma}^z_{7} - 4\hat{\sigma}^z_{30}\hat{\sigma}^z_{70} - 4\hat{\sigma}^z_{30}\hat{\sigma}^z_{71} + 2\hat{\sigma}^z_{30}\hat{\sigma}^z_{8} + 2\hat{\sigma}^z_{30}\hat{\sigma}^z_{9} + 404\hat{\sigma}^z_{30} +$$
$$4\hat{\sigma}^z_{31}\hat{\sigma}^z_{32} + 4\hat{\sigma}^z_{31}\hat{\sigma}^z_{33} + 4\hat{\sigma}^z_{31}\hat{\sigma}^z_{34} + 4\hat{\sigma}^z_{31}\hat{\sigma}^z_{35} + 2\hat{\sigma}^z_{31}\hat{\sigma}^z_{4} - 4\hat{\sigma}^z_{31}\hat{\sigma}^z_{48} - 4\hat{\sigma}^z_{31}\hat{\sigma}^z_{49} + 2\hat{\sigma}^z_{31}\hat{\sigma}^z_{5} - 4\hat{\sigma}^z_{31}\hat{\sigma}^z_{50} - 4\hat{\sigma}^z_{31}\hat{\sigma}^z_{51} - 4\hat{\sigma}^z_{31}\hat{\sigma}^z_{52} -$$
$$4\hat{\sigma}^z_{31}\hat{\sigma}^z_{53} - 4\hat{\sigma}^z_{31}\hat{\sigma}^z_{54} - 360\hat{\sigma}^z_{31}\hat{\sigma}^z_{55} - 4\hat{\sigma}^z_{31}\hat{\sigma}^z_{56} - 4\hat{\sigma}^z_{31}\hat{\sigma}^z_{57} - 4\hat{\sigma}^z_{31}\hat{\sigma}^z_{58} - 4\hat{\sigma}^z_{31}\hat{\sigma}^z_{59} + 2\hat{\sigma}^z_{31}\hat{\sigma}^z_{6} - 4\hat{\sigma}^z_{31}\hat{\sigma}^z_{60} - 4\hat{\sigma}^z_{31}\hat{\sigma}^z_{61} -$$
$$4\hat{\sigma}^z_{31}\hat{\sigma}^z_{62} - 4\hat{\sigma}^z_{31}\hat{\sigma}^z_{63} - 4\hat{\sigma}^z_{31}\hat{\sigma}^z_{64} - 4\hat{\sigma}^z_{31}\hat{\sigma}^z_{65} - 4\hat{\sigma}^z_{31}\hat{\sigma}^z_{66} - 360\hat{\sigma}^z_{31}\hat{\sigma}^z_{67} - 4\hat{\sigma}^z_{31}\hat{\sigma}^z_{68} - 4\hat{\sigma}^z_{31}\hat{\sigma}^z_{69} + 180\hat{\sigma}^z_{31}\hat{\sigma}^z_{7} - 4\hat{\sigma}^z_{31}\hat{\sigma}^z_{70} -$$
$$4\hat{\sigma}^z_{31}\hat{\sigma}^z_{71} + 2\hat{\sigma}^z_{31}\hat{\sigma}^z_{8} + 2\hat{\sigma}^z_{31}\hat{\sigma}^z_{9} + 404\hat{\sigma}^z_{31} + 4\hat{\sigma}^z_{32}\hat{\sigma}^z_{33} + 4\hat{\sigma}^z_{32}\hat{\sigma}^z_{34} + 4\hat{\sigma}^z_{32}\hat{\sigma}^z_{35} + 2\hat{\sigma}^z_{32}\hat{\sigma}^z_{4} - 4\hat{\sigma}^z_{32}\hat{\sigma}^z_{48} - 4\hat{\sigma}^z_{32}\hat{\sigma}^z_{49} + 2\hat{\sigma}^z_{32}\hat{\sigma}^z_{5} -$$
$$4\hat{\sigma}^z_{32}\hat{\sigma}^z_{50} - 4\hat{\sigma}^z_{32}\hat{\sigma}^z_{51} - 4\hat{\sigma}^z_{32}\hat{\sigma}^z_{52} - 4\hat{\sigma}^z_{32}\hat{\sigma}^z_{53} - 4\hat{\sigma}^z_{32}\hat{\sigma}^z_{54} - 4\hat{\sigma}^z_{32}\hat{\sigma}^z_{55} - 360\hat{\sigma}^z_{32}\hat{\sigma}^z_{56} - 4\hat{\sigma}^z_{32}\hat{\sigma}^z_{57} - 4\hat{\sigma}^z_{32}\hat{\sigma}^z_{58} - 4\hat{\sigma}^z_{32}\hat{\sigma}^z_{59} +$$
$$2\hat{\sigma}^z_{32}\hat{\sigma}^z_{6} - 4\hat{\sigma}^z_{32}\hat{\sigma}^z_{60} - 4\hat{\sigma}^z_{32}\hat{\sigma}^z_{61} - 4\hat{\sigma}^z_{32}\hat{\sigma}^z_{62} - 4\hat{\sigma}^z_{32}\hat{\sigma}^z_{63} - 4\hat{\sigma}^z_{32}\hat{\sigma}^z_{64} - 4\hat{\sigma}^z_{32}\hat{\sigma}^z_{65} - 4\hat{\sigma}^z_{32}\hat{\sigma}^z_{66} - 4\hat{\sigma}^z_{32}\hat{\sigma}^z_{67} - 360\hat{\sigma}^z_{32}\hat{\sigma}^z_{68} -$$
$$4\hat{\sigma}^z_{32}\hat{\sigma}^z_{69} + 2\hat{\sigma}^z_{32}\hat{\sigma}^z_{7} - 4\hat{\sigma}^z_{32}\hat{\sigma}^z_{70} - 4\hat{\sigma}^z_{32}\hat{\sigma}^z_{71} + 180\hat{\sigma}^z_{32}\hat{\sigma}^z_{8} + 2\hat{\sigma}^z_{32}\hat{\sigma}^z_{9} + 404\hat{\sigma}^z_{32} + 4\hat{\sigma}^z_{33}\hat{\sigma}^z_{34} + 4\hat{\sigma}^z_{33}\hat{\sigma}^z_{35} + 2\hat{\sigma}^z_{33}\hat{\sigma}^z_{4} - 4\hat{\sigma}^z_{33}\hat{\sigma}^z_{48} -$$
$$4\hat{\sigma}^z_{33}\hat{\sigma}^z_{49} + 2\hat{\sigma}^z_{33}\hat{\sigma}^z_{5} - 4\hat{\sigma}^z_{33}\hat{\sigma}^z_{50} - 4\hat{\sigma}^z_{33}\hat{\sigma}^z_{51} - 4\hat{\sigma}^z_{33}\hat{\sigma}^z_{52} - 4\hat{\sigma}^z_{33}\hat{\sigma}^z_{53} - 4\hat{\sigma}^z_{33}\hat{\sigma}^z_{54} - 4\hat{\sigma}^z_{33}\hat{\sigma}^z_{55} - 4\hat{\sigma}^z_{33}\hat{\sigma}^z_{56} - 360\hat{\sigma}^z_{33}\hat{\sigma}^z_{57} -$$
$$4\hat{\sigma}^z_{33}\hat{\sigma}^z_{58} - 4\hat{\sigma}^z_{33}\hat{\sigma}^z_{59} + 2\hat{\sigma}^z_{33}\hat{\sigma}^z_{6} - 4\hat{\sigma}^z_{33}\hat{\sigma}^z_{60} - 4\hat{\sigma}^z_{33}\hat{\sigma}^z_{61} - 4\hat{\sigma}^z_{33}\hat{\sigma}^z_{62} - 4\hat{\sigma}^z_{33}\hat{\sigma}^z_{63} - 4\hat{\sigma}^z_{33}\hat{\sigma}^z_{64} - 4\hat{\sigma}^z_{33}\hat{\sigma}^z_{65} - 4\hat{\sigma}^z_{33}\hat{\sigma}^z_{66} - 4\hat{\sigma}^z_{33}\hat{\sigma}^z_{67} -$$
$$4\hat{\sigma}^z_{33}\hat{\sigma}^z_{68} - 360\hat{\sigma}^z_{33}\hat{\sigma}^z_{69} + 2\hat{\sigma}^z_{33}\hat{\sigma}^z_{7} - 4\hat{\sigma}^z_{33}\hat{\sigma}^z_{70} - 4\hat{\sigma}^z_{33}\hat{\sigma}^z_{71} + 2\hat{\sigma}^z_{33}\hat{\sigma}^z_{8} + 180\hat{\sigma}^z_{33}\hat{\sigma}^z_{9} + 404\hat{\sigma}^z_{33} + 4\hat{\sigma}^z_{34}\hat{\sigma}^z_{35} + 2\hat{\sigma}^z_{34}\hat{\sigma}^z_{4} -$$
$$4\hat{\sigma}^z_{34}\hat{\sigma}^z_{48} - 4\hat{\sigma}^z_{34}\hat{\sigma}^z_{49} + 2\hat{\sigma}^z_{34}\hat{\sigma}^z_{5} - 4\hat{\sigma}^z_{34}\hat{\sigma}^z_{50} - 4\hat{\sigma}^z_{34}\hat{\sigma}^z_{51} - 4\hat{\sigma}^z_{34}\hat{\sigma}^z_{52} - 4\hat{\sigma}^z_{34}\hat{\sigma}^z_{53} - 4\hat{\sigma}^z_{34}\hat{\sigma}^z_{54} - 4\hat{\sigma}^z_{34}\hat{\sigma}^z_{55} - 4\hat{\sigma}^z_{34}\hat{\sigma}^z_{56} - 4\hat{\sigma}^z_{34}\hat{\sigma}^z_{57} -$$
$$360\hat{\sigma}^z_{34}\hat{\sigma}^z_{58} - 4\hat{\sigma}^z_{34}\hat{\sigma}^z_{59} + 2\hat{\sigma}^z_{34}\hat{\sigma}^z_{6} - 4\hat{\sigma}^z_{34}\hat{\sigma}^z_{60} - 4\hat{\sigma}^z_{34}\hat{\sigma}^z_{61} - 4\hat{\sigma}^z_{34}\hat{\sigma}^z_{62} - 4\hat{\sigma}^z_{34}\hat{\sigma}^z_{63} - 4\hat{\sigma}^z_{34}\hat{\sigma}^z_{64} - 4\hat{\sigma}^z_{34}\hat{\sigma}^z_{65} - 4\hat{\sigma}^z_{34}\hat{\sigma}^z_{66} -$$
$$4\hat{\sigma}^z_{34}\hat{\sigma}^z_{67} - 4\hat{\sigma}^z_{34}\hat{\sigma}^z_{68} - 4\hat{\sigma}^z_{34}\hat{\sigma}^z_{69} + 2\hat{\sigma}^z_{34}\hat{\sigma}^z_{7} - 360\hat{\sigma}^z_{34}\hat{\sigma}^z_{70} - 4\hat{\sigma}^z_{34}\hat{\sigma}^z_{71} + 2\hat{\sigma}^z_{34}\hat{\sigma}^z_{8} + 2\hat{\sigma}^z_{34}\hat{\sigma}^z_{9} + 404\hat{\sigma}^z_{34} + 2\hat{\sigma}^z_{35}\hat{\sigma}^z_{4} - 4\hat{\sigma}^z_{35}\hat{\sigma}^z_{48} -$$
$$4\hat{\sigma}^z_{35}\hat{\sigma}^z_{49} + 2\hat{\sigma}^z_{35}\hat{\sigma}^z_{5} - 4\hat{\sigma}^z_{35}\hat{\sigma}^z_{50} - 4\hat{\sigma}^z_{35}\hat{\sigma}^z_{51} - 4\hat{\sigma}^z_{35}\hat{\sigma}^z_{52} - 4\hat{\sigma}^z_{35}\hat{\sigma}^z_{53} - 4\hat{\sigma}^z_{35}\hat{\sigma}^z_{54} - 4\hat{\sigma}^z_{35}\hat{\sigma}^z_{55} - 4\hat{\sigma}^z_{35}\hat{\sigma}^z_{56} - 4\hat{\sigma}^z_{35}\hat{\sigma}^z_{57} - 4\hat{\sigma}^z_{35}\hat{\sigma}^z_{58} -$$
$$360\hat{\sigma}^z_{35}\hat{\sigma}^z_{59} + 2\hat{\sigma}^z_{35}\hat{\sigma}^z_{6} - 4\hat{\sigma}^z_{35}\hat{\sigma}^z_{60} - 4\hat{\sigma}^z_{35}\hat{\sigma}^z_{61} - 4\hat{\sigma}^z_{35}\hat{\sigma}^z_{62} - 4\hat{\sigma}^z_{35}\hat{\sigma}^z_{63} - 4\hat{\sigma}^z_{35}\hat{\sigma}^z_{64} - 4\hat{\sigma}^z_{35}\hat{\sigma}^z_{65} - 4\hat{\sigma}^z_{35}\hat{\sigma}^z_{66} - 4\hat{\sigma}^z_{35}\hat{\sigma}^z_{67} -$$
$$4\hat{\sigma}^z_{35}\hat{\sigma}^z_{68} - 4\hat{\sigma}^z_{35}\hat{\sigma}^z_{69} + 2\hat{\sigma}^z_{35}\hat{\sigma}^z_{7} - 4\hat{\sigma}^z_{35}\hat{\sigma}^z_{70} - 360\hat{\sigma}^z_{35}\hat{\sigma}^z_{71} + 2\hat{\sigma}^z_{35}\hat{\sigma}^z_{8} + 2\hat{\sigma}^z_{35}\hat{\sigma}^z_{9} + 404\hat{\sigma}^z_{35} + 8\hat{\sigma}^z_{36}\hat{\sigma}^z_{37} + 8\hat{\sigma}^z_{36}\hat{\sigma}^z_{38} + 8\hat{\sigma}^z_{36}\hat{\sigma}^z_{39} -$$
$$4\hat{\sigma}^z_{36}\hat{\sigma}^z_{4} + 8\hat{\sigma}^z_{36}\hat{\sigma}^z_{40} + 8\hat{\sigma}^z_{36}\hat{\sigma}^z_{41} + 8\hat{\sigma}^z_{36}\hat{\sigma}^z_{42} + 8\hat{\sigma}^z_{36}\hat{\sigma}^z_{43} + 8\hat{\sigma}^z_{36}\hat{\sigma}^z_{44} + 8\hat{\sigma}^z_{36}\hat{\sigma}^z_{45} + 8\hat{\sigma}^z_{36}\hat{\sigma}^z_{46} + 8\hat{\sigma}^z_{36}\hat{\sigma}^z_{47} - 4\hat{\sigma}^z_{36}\hat{\sigma}^z_{5} - 4\hat{\sigma}^z_{36}\hat{\sigma}^z_{6} -$$
$$4\hat{\sigma}^z_{36}\hat{\sigma}^z_{7} - 4\hat{\sigma}^z_{36}\hat{\sigma}^z_{8} - 4\hat{\sigma}^z_{36}\hat{\sigma}^z_{9} - 404\hat{\sigma}^z_{36} + 8\hat{\sigma}^z_{37}\hat{\sigma}^z_{38} + 8\hat{\sigma}^z_{37}\hat{\sigma}^z_{39} - 4\hat{\sigma}^z_{37}\hat{\sigma}^z_{4} + 8\hat{\sigma}^z_{37}\hat{\sigma}^z_{40} + 8\hat{\sigma}^z_{37}\hat{\sigma}^z_{41} + 8\hat{\sigma}^z_{37}\hat{\sigma}^z_{42} + 8\hat{\sigma}^z_{37}\hat{\sigma}^z_{43} +$$
$$8\hat{\sigma}^z_{37}\hat{\sigma}^z_{44} + 8\hat{\sigma}^z_{37}\hat{\sigma}^z_{45} + 8\hat{\sigma}^z_{37}\hat{\sigma}^z_{46} + 8\hat{\sigma}^z_{37}\hat{\sigma}^z_{47} - 4\hat{\sigma}^z_{37}\hat{\sigma}^z_{5} - 4\hat{\sigma}^z_{37}\hat{\sigma}^z_{6} - 4\hat{\sigma}^z_{37}\hat{\sigma}^z_{7} - 4\hat{\sigma}^z_{37}\hat{\sigma}^z_{8} - 4\hat{\sigma}^z_{37}\hat{\sigma}^z_{9} - 404\hat{\sigma}^z_{37} + 8\hat{\sigma}^z_{38}\hat{\sigma}^z_{39} -$$
$$4\hat{\sigma}^z_{38}\hat{\sigma}^z_{4} + 8\hat{\sigma}^z_{38}\hat{\sigma}^z_{40} + 8\hat{\sigma}^z_{38}\hat{\sigma}^z_{41} + 8\hat{\sigma}^z_{38}\hat{\sigma}^z_{42} + 8\hat{\sigma}^z_{38}\hat{\sigma}^z_{43} + 8\hat{\sigma}^z_{38}\hat{\sigma}^z_{44} + 8\hat{\sigma}^z_{38}\hat{\sigma}^z_{45} + 8\hat{\sigma}^z_{38}\hat{\sigma}^z_{46} + 8\hat{\sigma}^z_{38}\hat{\sigma}^z_{47} - 4\hat{\sigma}^z_{38}\hat{\sigma}^z_{5} - 4\hat{\sigma}^z_{38}\hat{\sigma}^z_{6} -$$
$$4\hat{\sigma}^z_{38}\hat{\sigma}^z_{7} - 4\hat{\sigma}^z_{38}\hat{\sigma}^z_{8} - 4\hat{\sigma}^z_{38}\hat{\sigma}^z_{9} - 404\hat{\sigma}^z_{38} - 4\hat{\sigma}^z_{39}\hat{\sigma}^z_{4} + 8\hat{\sigma}^z_{39}\hat{\sigma}^z_{40} + 8\hat{\sigma}^z_{39}\hat{\sigma}^z_{41} + 8\hat{\sigma}^z_{39}\hat{\sigma}^z_{42} + 8\hat{\sigma}^z_{39}\hat{\sigma}^z_{43} + 8\hat{\sigma}^z_{39}\hat{\sigma}^z_{44} + 8\hat{\sigma}^z_{39}\hat{\sigma}^z_{45} +$$
$$8\hat{\sigma}^z_{39}\hat{\sigma}^z_{46} + 8\hat{\sigma}^z_{39}\hat{\sigma}^z_{47} - 4\hat{\sigma}^z_{39}\hat{\sigma}^z_{5} - 4\hat{\sigma}^z_{39}\hat{\sigma}^z_{6} - 4\hat{\sigma}^z_{39}\hat{\sigma}^z_{7} - 4\hat{\sigma}^z_{39}\hat{\sigma}^z_{8} - 4\hat{\sigma}^z_{39}\hat{\sigma}^z_{9} - 404\hat{\sigma}^z_{39} - 360\hat{\sigma}^z_{4}\hat{\sigma}^z_{40} - 4\hat{\sigma}^z_{4}\hat{\sigma}^z_{41} - 4\hat{\sigma}^z_{4}\hat{\sigma}^z_{42} -$$
$$4\hat{\sigma}^z_{4}\hat{\sigma}^z_{43} - 4\hat{\sigma}^z_{4}\hat{\sigma}^z_{44} - 4\hat{\sigma}^z_{4}\hat{\sigma}^z_{45} - 4\hat{\sigma}^z_{4}\hat{\sigma}^z_{46} - 4\hat{\sigma}^z_{4}\hat{\sigma}^z_{47} - 4\hat{\sigma}^z_{4}\hat{\sigma}^z_{48} - 4\hat{\sigma}^z_{4}\hat{\sigma}^z_{49} + 4\hat{\sigma}^z_{4}\hat{\sigma}^z_{5} - 4\hat{\sigma}^z_{4}\hat{\sigma}^z_{50} - 4\hat{\sigma}^z_{4}\hat{\sigma}^z_{51} - 360\hat{\sigma}^z_{4}\hat{\sigma}^z_{52} -$$
$$4\hat{\sigma}^z_{4}\hat{\sigma}^z_{53} - 4\hat{\sigma}^z_{4}\hat{\sigma}^z_{54} - 4\hat{\sigma}^z_{4}\hat{\sigma}^z_{55} - 4\hat{\sigma}^z_{4}\hat{\sigma}^z_{56} - 4\hat{\sigma}^z_{4}\hat{\sigma}^z_{57} - 4\hat{\sigma}^z_{4}\hat{\sigma}^z_{58} - 4\hat{\sigma}^z_{4}\hat{\sigma}^z_{59} + 4\hat{\sigma}^z_{4}\hat{\sigma}^z_{6} + 4\hat{\sigma}^z_{4}\hat{\sigma}^z_{7} + 4\hat{\sigma}^z_{4}\hat{\sigma}^z_{8} + 4\hat{\sigma}^z_{4}\hat{\sigma}^z_{9} +$$
$$404\hat{\sigma}^z_{4} + 8\hat{\sigma}^z_{40}\hat{\sigma}^z_{41} + 8\hat{\sigma}^z_{40}\hat{\sigma}^z_{42} + 8\hat{\sigma}^z_{40}\hat{\sigma}^z_{43} + 8\hat{\sigma}^z_{40}\hat{\sigma}^z_{44} + 8\hat{\sigma}^z_{40}\hat{\sigma}^z_{45} + 8\hat{\sigma}^z_{40}\hat{\sigma}^z_{46} + 8\hat{\sigma}^z_{40}\hat{\sigma}^z_{47} - 4\hat{\sigma}^z_{40}\hat{\sigma}^z_{5} - 4\hat{\sigma}^z_{40}\hat{\sigma}^z_{6} - 4\hat{\sigma}^z_{40}\hat{\sigma}^z_{7} -$$
$$4\hat{\sigma}^z_{40}\hat{\sigma}^z_{8} - 4\hat{\sigma}^z_{40}\hat{\sigma}^z_{9} - 404\hat{\sigma}^z_{40} + 8\hat{\sigma}^z_{41}\hat{\sigma}^z_{42} + 8\hat{\sigma}^z_{41}\hat{\sigma}^z_{43} + 8\hat{\sigma}^z_{41}\hat{\sigma}^z_{44} + 8\hat{\sigma}^z_{41}\hat{\sigma}^z_{45} + 8\hat{\sigma}^z_{41}\hat{\sigma}^z_{46} + 8\hat{\sigma}^z_{41}\hat{\sigma}^z_{47} - 360\hat{\sigma}^z_{41}\hat{\sigma}^z_{5} - 4\hat{\sigma}^z_{41}\hat{\sigma}^z_{6} -$$
$$4\hat{\sigma}^z_{41}\hat{\sigma}^z_{7} - 4\hat{\sigma}^z_{41}\hat{\sigma}^z_{8} - 4\hat{\sigma}^z_{41}\hat{\sigma}^z_{9} - 404\hat{\sigma}^z_{41} + 8\hat{\sigma}^z_{42}\hat{\sigma}^z_{43} + 8\hat{\sigma}^z_{42}\hat{\sigma}^z_{44} + 8\hat{\sigma}^z_{42}\hat{\sigma}^z_{45} + 8\hat{\sigma}^z_{42}\hat{\sigma}^z_{46} + 8\hat{\sigma}^z_{42}\hat{\sigma}^z_{47} - 4\hat{\sigma}^z_{42}\hat{\sigma}^z_{5} - 360\hat{\sigma}^z_{42}\hat{\sigma}^z_{6} -$$

$$4\hat{\sigma}_{42}^{z}\hat{\sigma}_{7}^{z}-4\hat{\sigma}_{42}^{z}\hat{\sigma}_{8}^{z}-4\hat{\sigma}_{42}^{z}\hat{\sigma}_{9}^{z}-404\hat{\sigma}_{42}^{z}+8\hat{\sigma}_{43}^{z}\hat{\sigma}_{44}^{z}+8\hat{\sigma}_{43}^{z}\hat{\sigma}_{45}^{z}+8\hat{\sigma}_{43}^{z}\hat{\sigma}_{46}^{z}+8\hat{\sigma}_{43}^{z}\hat{\sigma}_{47}^{z}-4\hat{\sigma}_{43}^{z}\hat{\sigma}_{5}^{z}-4\hat{\sigma}_{43}^{z}\hat{\sigma}_{6}^{z}-360\hat{\sigma}_{43}^{z}\hat{\sigma}_{7}^{z}-$$
$$4\hat{\sigma}_{43}^{z}\hat{\sigma}_{8}^{z}-4\hat{\sigma}_{43}^{z}\hat{\sigma}_{9}^{z}-404\hat{\sigma}_{43}^{z}+8\hat{\sigma}_{44}^{z}\hat{\sigma}_{45}^{z}+8\hat{\sigma}_{44}^{z}\hat{\sigma}_{46}^{z}+8\hat{\sigma}_{44}^{z}\hat{\sigma}_{47}^{z}-4\hat{\sigma}_{44}^{z}\hat{\sigma}_{5}^{z}-4\hat{\sigma}_{44}^{z}\hat{\sigma}_{6}^{z}-4\hat{\sigma}_{44}^{z}\hat{\sigma}_{7}^{z}-360\hat{\sigma}_{44}^{z}\hat{\sigma}_{8}^{z}-4\hat{\sigma}_{44}^{z}\hat{\sigma}_{9}^{z}-$$
$$404\hat{\sigma}_{44}^{z}+8\hat{\sigma}_{45}^{z}\hat{\sigma}_{46}^{z}+8\hat{\sigma}_{45}^{z}\hat{\sigma}_{47}^{z}-4\hat{\sigma}_{45}^{z}\hat{\sigma}_{5}^{z}-4\hat{\sigma}_{45}^{z}\hat{\sigma}_{6}^{z}-4\hat{\sigma}_{45}^{z}\hat{\sigma}_{7}^{z}-4\hat{\sigma}_{45}^{z}\hat{\sigma}_{8}^{z}-360\hat{\sigma}_{45}^{z}\hat{\sigma}_{9}^{z}-404\hat{\sigma}_{45}^{z}+8\hat{\sigma}_{46}^{z}\hat{\sigma}_{47}^{z}-4\hat{\sigma}_{46}^{z}\hat{\sigma}_{5}^{z}-$$
$$4\hat{\sigma}_{46}^{z}\hat{\sigma}_{6}^{z}-4\hat{\sigma}_{46}^{z}\hat{\sigma}_{7}^{z}-4\hat{\sigma}_{46}^{z}\hat{\sigma}_{8}^{z}-4\hat{\sigma}_{46}^{z}\hat{\sigma}_{9}^{z}-404\hat{\sigma}_{46}^{z}-4\hat{\sigma}_{47}^{z}\hat{\sigma}_{5}^{z}-4\hat{\sigma}_{47}^{z}\hat{\sigma}_{6}^{z}-4\hat{\sigma}_{47}^{z}\hat{\sigma}_{7}^{z}-4\hat{\sigma}_{47}^{z}\hat{\sigma}_{8}^{z}-4\hat{\sigma}_{47}^{z}\hat{\sigma}_{9}^{z}-404\hat{\sigma}_{47}^{z}+$$
$$8\hat{\sigma}_{48}^{z}\hat{\sigma}_{49}^{z}-4\hat{\sigma}_{48}^{z}\hat{\sigma}_{5}^{z}+8\hat{\sigma}_{48}^{z}\hat{\sigma}_{50}^{z}+8\hat{\sigma}_{48}^{z}\hat{\sigma}_{51}^{z}+8\hat{\sigma}_{48}^{z}\hat{\sigma}_{52}^{z}+8\hat{\sigma}_{48}^{z}\hat{\sigma}_{53}^{z}+8\hat{\sigma}_{48}^{z}\hat{\sigma}_{54}^{z}+8\hat{\sigma}_{48}^{z}\hat{\sigma}_{55}^{z}+8\hat{\sigma}_{48}^{z}\hat{\sigma}_{56}^{z}+8\hat{\sigma}_{48}^{z}\hat{\sigma}_{57}^{z}+8\hat{\sigma}_{48}^{z}\hat{\sigma}_{58}^{z}+$$
$$8\hat{\sigma}_{48}^{z}\hat{\sigma}_{59}^{z}-4\hat{\sigma}_{48}^{z}\hat{\sigma}_{6}^{z}-4\hat{\sigma}_{48}^{z}\hat{\sigma}_{7}^{z}-4\hat{\sigma}_{48}^{z}\hat{\sigma}_{8}^{z}-4\hat{\sigma}_{48}^{z}\hat{\sigma}_{9}^{z}-404\hat{\sigma}_{48}^{z}-4\hat{\sigma}_{49}^{z}\hat{\sigma}_{5}^{z}+8\hat{\sigma}_{49}^{z}\hat{\sigma}_{50}^{z}+8\hat{\sigma}_{49}^{z}\hat{\sigma}_{51}^{z}+8\hat{\sigma}_{49}^{z}\hat{\sigma}_{52}^{z}+8\hat{\sigma}_{49}^{z}\hat{\sigma}_{53}^{z}+$$
$$8\hat{\sigma}_{49}^{z}\hat{\sigma}_{54}^{z}+8\hat{\sigma}_{49}^{z}\hat{\sigma}_{55}^{z}+8\hat{\sigma}_{49}^{z}\hat{\sigma}_{56}^{z}+8\hat{\sigma}_{49}^{z}\hat{\sigma}_{57}^{z}+8\hat{\sigma}_{49}^{z}\hat{\sigma}_{58}^{z}+8\hat{\sigma}_{49}^{z}\hat{\sigma}_{59}^{z}-4\hat{\sigma}_{49}^{z}\hat{\sigma}_{6}^{z}-4\hat{\sigma}_{49}^{z}\hat{\sigma}_{7}^{z}-4\hat{\sigma}_{49}^{z}\hat{\sigma}_{8}^{z}-4\hat{\sigma}_{49}^{z}\hat{\sigma}_{9}^{z}-404\hat{\sigma}_{49}^{z}-$$
$$4\hat{\sigma}_{5}^{z}\hat{\sigma}_{50}^{z}-4\hat{\sigma}_{5}^{z}\hat{\sigma}_{51}^{z}-4\hat{\sigma}_{5}^{z}\hat{\sigma}_{52}^{z}-360\hat{\sigma}_{5}^{z}\hat{\sigma}_{53}^{z}-4\hat{\sigma}_{5}^{z}\hat{\sigma}_{54}^{z}-4\hat{\sigma}_{5}^{z}\hat{\sigma}_{55}^{z}-4\hat{\sigma}_{5}^{z}\hat{\sigma}_{56}^{z}-4\hat{\sigma}_{5}^{z}\hat{\sigma}_{57}^{z}-4\hat{\sigma}_{5}^{z}\hat{\sigma}_{58}^{z}-4\hat{\sigma}_{5}^{z}\hat{\sigma}_{59}^{z}+4\hat{\sigma}_{5}^{z}\hat{\sigma}_{6}^{z}+$$
$$4\hat{\sigma}_{5}^{z}\hat{\sigma}_{7}^{z}+4\hat{\sigma}_{5}^{z}\hat{\sigma}_{8}^{z}+4\hat{\sigma}_{5}^{z}\hat{\sigma}_{9}^{z}+404\hat{\sigma}_{5}^{z}+8\hat{\sigma}_{50}^{z}\hat{\sigma}_{51}^{z}+8\hat{\sigma}_{50}^{z}\hat{\sigma}_{52}^{z}+8\hat{\sigma}_{50}^{z}\hat{\sigma}_{53}^{z}+8\hat{\sigma}_{50}^{z}\hat{\sigma}_{54}^{z}+8\hat{\sigma}_{50}^{z}\hat{\sigma}_{55}^{z}+8\hat{\sigma}_{50}^{z}\hat{\sigma}_{56}^{z}+8\hat{\sigma}_{50}^{z}\hat{\sigma}_{57}^{z}+$$
$$8\hat{\sigma}_{50}^{z}\hat{\sigma}_{58}^{z}+8\hat{\sigma}_{50}^{z}\hat{\sigma}_{59}^{z}-4\hat{\sigma}_{50}^{z}\hat{\sigma}_{6}^{z}-4\hat{\sigma}_{50}^{z}\hat{\sigma}_{7}^{z}-4\hat{\sigma}_{50}^{z}\hat{\sigma}_{8}^{z}-4\hat{\sigma}_{50}^{z}\hat{\sigma}_{9}^{z}-404\hat{\sigma}_{50}^{z}+8\hat{\sigma}_{51}^{z}\hat{\sigma}_{52}^{z}+8\hat{\sigma}_{51}^{z}\hat{\sigma}_{53}^{z}+8\hat{\sigma}_{51}^{z}\hat{\sigma}_{54}^{z}+8\hat{\sigma}_{51}^{z}\hat{\sigma}_{55}^{z}+$$
$$8\hat{\sigma}_{51}^{z}\hat{\sigma}_{56}^{z}+8\hat{\sigma}_{51}^{z}\hat{\sigma}_{57}^{z}+8\hat{\sigma}_{51}^{z}\hat{\sigma}_{58}^{z}+8\hat{\sigma}_{51}^{z}\hat{\sigma}_{59}^{z}-4\hat{\sigma}_{51}^{z}\hat{\sigma}_{6}^{z}-4\hat{\sigma}_{51}^{z}\hat{\sigma}_{7}^{z}-4\hat{\sigma}_{51}^{z}\hat{\sigma}_{8}^{z}-4\hat{\sigma}_{51}^{z}\hat{\sigma}_{9}^{z}-404\hat{\sigma}_{51}^{z}+8\hat{\sigma}_{52}^{z}\hat{\sigma}_{53}^{z}+8\hat{\sigma}_{52}^{z}\hat{\sigma}_{54}^{z}+$$
$$8\hat{\sigma}_{52}^{z}\hat{\sigma}_{55}^{z}+8\hat{\sigma}_{52}^{z}\hat{\sigma}_{56}^{z}+8\hat{\sigma}_{52}^{z}\hat{\sigma}_{57}^{z}+8\hat{\sigma}_{52}^{z}\hat{\sigma}_{58}^{z}+8\hat{\sigma}_{52}^{z}\hat{\sigma}_{59}^{z}-4\hat{\sigma}_{52}^{z}\hat{\sigma}_{6}^{z}-4\hat{\sigma}_{52}^{z}\hat{\sigma}_{7}^{z}-4\hat{\sigma}_{52}^{z}\hat{\sigma}_{8}^{z}-4\hat{\sigma}_{52}^{z}\hat{\sigma}_{9}^{z}-404\hat{\sigma}_{52}^{z}+8\hat{\sigma}_{53}^{z}\hat{\sigma}_{54}^{z}+$$
$$8\hat{\sigma}_{53}^{z}\hat{\sigma}_{55}^{z}+8\hat{\sigma}_{53}^{z}\hat{\sigma}_{56}^{z}+8\hat{\sigma}_{53}^{z}\hat{\sigma}_{57}^{z}+8\hat{\sigma}_{53}^{z}\hat{\sigma}_{58}^{z}+8\hat{\sigma}_{53}^{z}\hat{\sigma}_{59}^{z}-4\hat{\sigma}_{53}^{z}\hat{\sigma}_{6}^{z}-4\hat{\sigma}_{53}^{z}\hat{\sigma}_{7}^{z}-4\hat{\sigma}_{53}^{z}\hat{\sigma}_{8}^{z}-4\hat{\sigma}_{53}^{z}\hat{\sigma}_{9}^{z}-404\hat{\sigma}_{53}^{z}+8\hat{\sigma}_{54}^{z}\hat{\sigma}_{55}^{z}+$$
$$8\hat{\sigma}_{54}^{z}\hat{\sigma}_{56}^{z}+8\hat{\sigma}_{54}^{z}\hat{\sigma}_{57}^{z}+8\hat{\sigma}_{54}^{z}\hat{\sigma}_{58}^{z}+8\hat{\sigma}_{54}^{z}\hat{\sigma}_{59}^{z}-360\hat{\sigma}_{54}^{z}\hat{\sigma}_{6}^{z}-4\hat{\sigma}_{54}^{z}\hat{\sigma}_{7}^{z}-4\hat{\sigma}_{54}^{z}\hat{\sigma}_{8}^{z}-4\hat{\sigma}_{54}^{z}\hat{\sigma}_{9}^{z}-404\hat{\sigma}_{54}^{z}+8\hat{\sigma}_{55}^{z}\hat{\sigma}_{56}^{z}+8\hat{\sigma}_{55}^{z}\hat{\sigma}_{57}^{z}+$$
$$8\hat{\sigma}_{55}^{z}\hat{\sigma}_{58}^{z}+8\hat{\sigma}_{55}^{z}\hat{\sigma}_{59}^{z}-4\hat{\sigma}_{55}^{z}\hat{\sigma}_{6}^{z}-360\hat{\sigma}_{55}^{z}\hat{\sigma}_{7}^{z}-4\hat{\sigma}_{55}^{z}\hat{\sigma}_{8}^{z}-4\hat{\sigma}_{55}^{z}\hat{\sigma}_{9}^{z}-404\hat{\sigma}_{55}^{z}+8\hat{\sigma}_{56}^{z}\hat{\sigma}_{57}^{z}+8\hat{\sigma}_{56}^{z}\hat{\sigma}_{58}^{z}+8\hat{\sigma}_{56}^{z}\hat{\sigma}_{59}^{z}-4\hat{\sigma}_{56}^{z}\hat{\sigma}_{6}^{z}-$$
$$4\hat{\sigma}_{56}^{z}\hat{\sigma}_{7}^{z}-360\hat{\sigma}_{56}^{z}\hat{\sigma}_{8}^{z}-4\hat{\sigma}_{56}^{z}\hat{\sigma}_{9}^{z}-404\hat{\sigma}_{56}^{z}+8\hat{\sigma}_{57}^{z}\hat{\sigma}_{58}^{z}+8\hat{\sigma}_{57}^{z}\hat{\sigma}_{59}^{z}-4\hat{\sigma}_{57}^{z}\hat{\sigma}_{6}^{z}-4\hat{\sigma}_{57}^{z}\hat{\sigma}_{7}^{z}-4\hat{\sigma}_{57}^{z}\hat{\sigma}_{8}^{z}-360\hat{\sigma}_{57}^{z}\hat{\sigma}_{9}^{z}-404\hat{\sigma}_{57}^{z}+$$
$$8\hat{\sigma}_{58}^{z}\hat{\sigma}_{59}^{z}-4\hat{\sigma}_{58}^{z}\hat{\sigma}_{6}^{z}-4\hat{\sigma}_{58}^{z}\hat{\sigma}_{7}^{z}-4\hat{\sigma}_{58}^{z}\hat{\sigma}_{8}^{z}-4\hat{\sigma}_{58}^{z}\hat{\sigma}_{9}^{z}-404\hat{\sigma}_{58}^{z}-4\hat{\sigma}_{59}^{z}\hat{\sigma}_{6}^{z}-4\hat{\sigma}_{59}^{z}\hat{\sigma}_{7}^{z}-4\hat{\sigma}_{59}^{z}\hat{\sigma}_{8}^{z}-4\hat{\sigma}_{59}^{z}\hat{\sigma}_{9}^{z}-404\hat{\sigma}_{59}^{z}+$$
$$4\hat{\sigma}_{6}^{z}\hat{\sigma}_{7}^{z}+4\hat{\sigma}_{6}^{z}\hat{\sigma}_{8}^{z}+4\hat{\sigma}_{6}^{z}\hat{\sigma}_{9}^{z}+404\hat{\sigma}_{6}^{z}+8\hat{\sigma}_{60}^{z}\hat{\sigma}_{61}^{z}+8\hat{\sigma}_{60}^{z}\hat{\sigma}_{62}^{z}+8\hat{\sigma}_{60}^{z}\hat{\sigma}_{63}^{z}+8\hat{\sigma}_{60}^{z}\hat{\sigma}_{64}^{z}+8\hat{\sigma}_{60}^{z}\hat{\sigma}_{65}^{z}+8\hat{\sigma}_{60}^{z}\hat{\sigma}_{66}^{z}+8\hat{\sigma}_{60}^{z}\hat{\sigma}_{67}^{z}+$$
$$8\hat{\sigma}_{60}^{z}\hat{\sigma}_{68}^{z}+8\hat{\sigma}_{60}^{z}\hat{\sigma}_{69}^{z}+8\hat{\sigma}_{60}^{z}\hat{\sigma}_{70}^{z}+8\hat{\sigma}_{60}^{z}\hat{\sigma}_{71}^{z}-404\hat{\sigma}_{60}^{z}+8\hat{\sigma}_{61}^{z}\hat{\sigma}_{62}^{z}+8\hat{\sigma}_{61}^{z}\hat{\sigma}_{63}^{z}+8\hat{\sigma}_{61}^{z}\hat{\sigma}_{64}^{z}+8\hat{\sigma}_{61}^{z}\hat{\sigma}_{65}^{z}+8\hat{\sigma}_{61}^{z}\hat{\sigma}_{66}^{z}+8\hat{\sigma}_{61}^{z}\hat{\sigma}_{67}^{z}+$$
$$8\hat{\sigma}_{61}^{z}\hat{\sigma}_{68}^{z}+8\hat{\sigma}_{61}^{z}\hat{\sigma}_{69}^{z}+8\hat{\sigma}_{61}^{z}\hat{\sigma}_{70}^{z}+8\hat{\sigma}_{61}^{z}\hat{\sigma}_{71}^{z}-404\hat{\sigma}_{61}^{z}+8\hat{\sigma}_{62}^{z}\hat{\sigma}_{63}^{z}+8\hat{\sigma}_{62}^{z}\hat{\sigma}_{64}^{z}+8\hat{\sigma}_{62}^{z}\hat{\sigma}_{65}^{z}+8\hat{\sigma}_{62}^{z}\hat{\sigma}_{66}^{z}+8\hat{\sigma}_{62}^{z}\hat{\sigma}_{67}^{z}+8\hat{\sigma}_{62}^{z}\hat{\sigma}_{68}^{z}+$$
$$8\hat{\sigma}_{62}^{z}\hat{\sigma}_{69}^{z}+8\hat{\sigma}_{62}^{z}\hat{\sigma}_{70}^{z}+8\hat{\sigma}_{62}^{z}\hat{\sigma}_{71}^{z}-404\hat{\sigma}_{62}^{z}+8\hat{\sigma}_{63}^{z}\hat{\sigma}_{64}^{z}+8\hat{\sigma}_{63}^{z}\hat{\sigma}_{65}^{z}+8\hat{\sigma}_{63}^{z}\hat{\sigma}_{66}^{z}+8\hat{\sigma}_{63}^{z}\hat{\sigma}_{67}^{z}+8\hat{\sigma}_{63}^{z}\hat{\sigma}_{68}^{z}+8\hat{\sigma}_{63}^{z}\hat{\sigma}_{69}^{z}+8\hat{\sigma}_{63}^{z}\hat{\sigma}_{70}^{z}+$$
$$8\hat{\sigma}_{63}^{z}\hat{\sigma}_{71}^{z}-404\hat{\sigma}_{63}^{z}+8\hat{\sigma}_{64}^{z}\hat{\sigma}_{65}^{z}+8\hat{\sigma}_{64}^{z}\hat{\sigma}_{66}^{z}+8\hat{\sigma}_{64}^{z}\hat{\sigma}_{67}^{z}+8\hat{\sigma}_{64}^{z}\hat{\sigma}_{68}^{z}+8\hat{\sigma}_{64}^{z}\hat{\sigma}_{69}^{z}+8\hat{\sigma}_{64}^{z}\hat{\sigma}_{70}^{z}+8\hat{\sigma}_{64}^{z}\hat{\sigma}_{71}^{z}-404\hat{\sigma}_{64}^{z}+8\hat{\sigma}_{65}^{z}\hat{\sigma}_{66}^{z}+$$
$$8\hat{\sigma}_{65}^{z}\hat{\sigma}_{67}^{z}+8\hat{\sigma}_{65}^{z}\hat{\sigma}_{68}^{z}+8\hat{\sigma}_{65}^{z}\hat{\sigma}_{69}^{z}+8\hat{\sigma}_{65}^{z}\hat{\sigma}_{70}^{z}+8\hat{\sigma}_{65}^{z}\hat{\sigma}_{71}^{z}-404\hat{\sigma}_{65}^{z}+8\hat{\sigma}_{66}^{z}\hat{\sigma}_{67}^{z}+8\hat{\sigma}_{66}^{z}\hat{\sigma}_{68}^{z}+8\hat{\sigma}_{66}^{z}\hat{\sigma}_{69}^{z}+8\hat{\sigma}_{66}^{z}\hat{\sigma}_{70}^{z}+8\hat{\sigma}_{66}^{z}\hat{\sigma}_{71}^{z}-$$
$$404\hat{\sigma}_{66}^{z}+8\hat{\sigma}_{67}^{z}\hat{\sigma}_{68}^{z}+8\hat{\sigma}_{67}^{z}\hat{\sigma}_{69}^{z}+8\hat{\sigma}_{67}^{z}\hat{\sigma}_{70}^{z}+8\hat{\sigma}_{67}^{z}\hat{\sigma}_{71}^{z}-404\hat{\sigma}_{67}^{z}+8\hat{\sigma}_{68}^{z}\hat{\sigma}_{69}^{z}+8\hat{\sigma}_{68}^{z}\hat{\sigma}_{70}^{z}+8\hat{\sigma}_{68}^{z}\hat{\sigma}_{71}^{z}-404\hat{\sigma}_{68}^{z}+8\hat{\sigma}_{69}^{z}\hat{\sigma}_{70}^{z}+$$
$$8\hat{\sigma}_{69}^{z}\hat{\sigma}_{71}^{z}-404\hat{\sigma}_{69}^{z}+4\hat{\sigma}_{7}^{z}\hat{\sigma}_{8}^{z}+4\hat{\sigma}_{7}^{z}\hat{\sigma}_{9}^{z}+404\hat{\sigma}_{7}^{z}+8\hat{\sigma}_{70}^{z}\hat{\sigma}_{71}^{z}-404\hat{\sigma}_{70}^{z}-404\hat{\sigma}_{71}^{z}+4\hat{\sigma}_{8}^{z}\hat{\sigma}_{9}^{z}+404\hat{\sigma}_{8}^{z}+404\hat{\sigma}_{9}^{z}+19,872$$

A complete expression of Eq.(21)

$$\hat{H}_2\left(\hat{\sigma}\right)=2\hat{\sigma}_{0}^{z}\hat{\sigma}_{1}^{z}+2\hat{\sigma}_{0}^{z}\hat{\sigma}_{10}^{z}-2\hat{\sigma}_{0}^{z}\hat{\sigma}_{11}^{z}+2\hat{\sigma}_{0}^{z}\hat{\sigma}_{12}^{z}-2\hat{\sigma}_{0}^{z}\hat{\sigma}_{13}^{z}+2\hat{\sigma}_{0}^{z}\hat{\sigma}_{14}^{z}-2\hat{\sigma}_{0}^{z}\hat{\sigma}_{15}^{z}+2\hat{\sigma}_{0}^{z}\hat{\sigma}_{16}^{z}+2\hat{\sigma}_{0}^{z}\hat{\sigma}_{17}^{z}-2\hat{\sigma}_{0}^{z}\hat{\sigma}_{18}^{z}-$$
$$2\hat{\sigma}_{0}^{z}\hat{\sigma}_{19}^{z}-2\hat{\sigma}_{0}^{z}\hat{\sigma}_{2}^{z}-2\hat{\sigma}_{0}^{z}\hat{\sigma}_{20}^{z}-2\hat{\sigma}_{0}^{z}\hat{\sigma}_{21}^{z}+2\hat{\sigma}_{0}^{z}\hat{\sigma}_{22}^{z}+2\hat{\sigma}_{0}^{z}\hat{\sigma}_{23}^{z}-2\hat{\sigma}_{0}^{z}\hat{\sigma}_{24}^{z}+2\hat{\sigma}_{0}^{z}\hat{\sigma}_{25}^{z}+2\hat{\sigma}_{0}^{z}\hat{\sigma}_{26}^{z}+2\hat{\sigma}_{0}^{z}\hat{\sigma}_{27}^{z}-2\hat{\sigma}_{0}^{z}\hat{\sigma}_{3}^{z}+$$
$$2\hat{\sigma}_{0}^{z}\hat{\sigma}_{4}^{z}+2\hat{\sigma}_{0}^{z}\hat{\sigma}_{5}^{z}+2\hat{\sigma}_{0}^{z}\hat{\sigma}_{6}^{z}+2\hat{\sigma}_{0}^{z}\hat{\sigma}_{7}^{z}-2\hat{\sigma}_{0}^{z}\hat{\sigma}_{8}^{z}-2\hat{\sigma}_{0}^{z}\hat{\sigma}_{9}^{z}-2\hat{\sigma}_{1}^{z}\hat{\sigma}_{10}^{z}+2\hat{\sigma}_{1}^{z}\hat{\sigma}_{11}^{z}-2\hat{\sigma}_{1}^{z}\hat{\sigma}_{12}^{z}+2\hat{\sigma}_{1}^{z}\hat{\sigma}_{13}^{z}-2\hat{\sigma}_{1}^{z}\hat{\sigma}_{14}^{z}+$$
$$2\hat{\sigma}_{1}^{z}\hat{\sigma}_{15}^{z}-2\hat{\sigma}_{1}^{z}\hat{\sigma}_{16}^{z}-2\hat{\sigma}_{1}^{z}\hat{\sigma}_{17}^{z}+2\hat{\sigma}_{1}^{z}\hat{\sigma}_{18}^{z}+2\hat{\sigma}_{1}^{z}\hat{\sigma}_{19}^{z}+2\hat{\sigma}_{1}^{z}\hat{\sigma}_{2}^{z}+2\hat{\sigma}_{1}^{z}\hat{\sigma}_{20}^{z}+2\hat{\sigma}_{1}^{z}\hat{\sigma}_{21}^{z}-2\hat{\sigma}_{1}^{z}\hat{\sigma}_{22}^{z}-2\hat{\sigma}_{1}^{z}\hat{\sigma}_{23}^{z}+2\hat{\sigma}_{1}^{z}\hat{\sigma}_{24}^{z}-$$
$$2\hat{\sigma}_{1}^{z}\hat{\sigma}_{25}^{z}-2\hat{\sigma}_{1}^{z}\hat{\sigma}_{26}^{z}-2\hat{\sigma}_{1}^{z}\hat{\sigma}_{27}^{z}+2\hat{\sigma}_{1}^{z}\hat{\sigma}_{3}^{z}-2\hat{\sigma}_{1}^{z}\hat{\sigma}_{4}^{z}-2\hat{\sigma}_{1}^{z}\hat{\sigma}_{5}^{z}-2\hat{\sigma}_{1}^{z}\hat{\sigma}_{6}^{z}-2\hat{\sigma}_{1}^{z}\hat{\sigma}_{7}^{z}+2\hat{\sigma}_{1}^{z}\hat{\sigma}_{8}^{z}+2\hat{\sigma}_{1}^{z}\hat{\sigma}_{9}^{z}+2\hat{\sigma}_{10}^{z}\hat{\sigma}_{11}^{z}-$$
$$2\hat{\sigma}_{10}^{z}\hat{\sigma}_{12}^{z}+2\hat{\sigma}_{10}^{z}\hat{\sigma}_{13}^{z}-2\hat{\sigma}_{10}^{z}\hat{\sigma}_{14}^{z}+2\hat{\sigma}_{10}^{z}\hat{\sigma}_{15}^{z}-2\hat{\sigma}_{10}^{z}\hat{\sigma}_{16}^{z}-2\hat{\sigma}_{10}^{z}\hat{\sigma}_{17}^{z}+2\hat{\sigma}_{10}^{z}\hat{\sigma}_{18}^{z}+2\hat{\sigma}_{10}^{z}\hat{\sigma}_{19}^{z}+2\hat{\sigma}_{10}^{z}\hat{\sigma}_{2}^{z}+2\hat{\sigma}_{10}^{z}\hat{\sigma}_{20}^{z}+$$
$$2\hat{\sigma}_{10}^{z}\hat{\sigma}_{21}^{z}-2\hat{\sigma}_{10}^{z}\hat{\sigma}_{22}^{z}-2\hat{\sigma}_{10}^{z}\hat{\sigma}_{23}^{z}+2\hat{\sigma}_{10}^{z}\hat{\sigma}_{24}^{z}-2\hat{\sigma}_{10}^{z}\hat{\sigma}_{25}^{z}-2\hat{\sigma}_{10}^{z}\hat{\sigma}_{26}^{z}-2\hat{\sigma}_{10}^{z}\hat{\sigma}_{27}^{z}+2\hat{\sigma}_{10}^{z}\hat{\sigma}_{3}^{z}-2\hat{\sigma}_{10}^{z}\hat{\sigma}_{4}^{z}-2\hat{\sigma}_{10}^{z}\hat{\sigma}_{5}^{z}-2\hat{\sigma}_{10}^{z}\hat{\sigma}_{6}^{z}-$$
$$2\hat{\sigma}_{10}^{z}\hat{\sigma}_{7}^{z}+2\hat{\sigma}_{10}^{z}\hat{\sigma}_{8}^{z}+2\hat{\sigma}_{10}^{z}\hat{\sigma}_{9}^{z}+2\hat{\sigma}_{11}^{z}\hat{\sigma}_{12}^{z}-2\hat{\sigma}_{11}^{z}\hat{\sigma}_{13}^{z}+2\hat{\sigma}_{11}^{z}\hat{\sigma}_{14}^{z}-2\hat{\sigma}_{11}^{z}\hat{\sigma}_{15}^{z}+2\hat{\sigma}_{11}^{z}\hat{\sigma}_{16}^{z}+2\hat{\sigma}_{11}^{z}\hat{\sigma}_{17}^{z}-2\hat{\sigma}_{11}^{z}\hat{\sigma}_{18}^{z}-2\hat{\sigma}_{11}^{z}\hat{\sigma}_{19}^{z}-$$
$$2\hat{\sigma}_{11}^{z}\hat{\sigma}_{2}^{z}-2\hat{\sigma}_{11}^{z}\hat{\sigma}_{20}^{z}-2\hat{\sigma}_{11}^{z}\hat{\sigma}_{21}^{z}+2\hat{\sigma}_{11}^{z}\hat{\sigma}_{22}^{z}+2\hat{\sigma}_{11}^{z}\hat{\sigma}_{23}^{z}-2\hat{\sigma}_{11}^{z}\hat{\sigma}_{24}^{z}+2\hat{\sigma}_{11}^{z}\hat{\sigma}_{25}^{z}+2\hat{\sigma}_{11}^{z}\hat{\sigma}_{26}^{z}+2\hat{\sigma}_{11}^{z}\hat{\sigma}_{27}^{z}-2\hat{\sigma}_{11}^{z}\hat{\sigma}_{3}^{z}+2\hat{\sigma}_{11}^{z}\hat{\sigma}_{4}^{z}+$$
$$2\hat{\sigma}_{11}^{z}\hat{\sigma}_{5}^{z}+2\hat{\sigma}_{11}^{z}\hat{\sigma}_{6}^{z}+2\hat{\sigma}_{11}^{z}\hat{\sigma}_{7}^{z}-2\hat{\sigma}_{11}^{z}\hat{\sigma}_{8}^{z}-2\hat{\sigma}_{11}^{z}\hat{\sigma}_{9}^{z}+2\hat{\sigma}_{12}^{z}\hat{\sigma}_{13}^{z}-2\hat{\sigma}_{12}^{z}\hat{\sigma}_{14}^{z}+2\hat{\sigma}_{12}^{z}\hat{\sigma}_{15}^{z}-2\hat{\sigma}_{12}^{z}\hat{\sigma}_{16}^{z}-2\hat{\sigma}_{12}^{z}\hat{\sigma}_{17}^{z}+2\hat{\sigma}_{12}^{z}\hat{\sigma}_{18}^{z}+$$
$$2\hat{\sigma}_{12}^{z}\hat{\sigma}_{19}^{z}+2\hat{\sigma}_{12}^{z}\hat{\sigma}_{2}^{z}+2\hat{\sigma}_{12}^{z}\hat{\sigma}_{20}^{z}+2\hat{\sigma}_{12}^{z}\hat{\sigma}_{21}^{z}-2\hat{\sigma}_{12}^{z}\hat{\sigma}_{22}^{z}-2\hat{\sigma}_{12}^{z}\hat{\sigma}_{23}^{z}+2\hat{\sigma}_{12}^{z}\hat{\sigma}_{24}^{z}-2\hat{\sigma}_{12}^{z}\hat{\sigma}_{25}^{z}-2\hat{\sigma}_{12}^{z}\hat{\sigma}_{26}^{z}-2\hat{\sigma}_{12}^{z}\hat{\sigma}_{27}^{z}+$$
$$2\hat{\sigma}_{12}^{z}\hat{\sigma}_{3}^{z}-2\hat{\sigma}_{12}^{z}\hat{\sigma}_{4}^{z}-2\hat{\sigma}_{12}^{z}\hat{\sigma}_{5}^{z}-2\hat{\sigma}_{12}^{z}\hat{\sigma}_{6}^{z}-2\hat{\sigma}_{12}^{z}\hat{\sigma}_{7}^{z}+2\hat{\sigma}_{12}^{z}\hat{\sigma}_{8}^{z}+2\hat{\sigma}_{12}^{z}\hat{\sigma}_{9}^{z}+2\hat{\sigma}_{13}^{z}\hat{\sigma}_{14}^{z}-2\hat{\sigma}_{13}^{z}\hat{\sigma}_{15}^{z}+2\hat{\sigma}_{13}^{z}\hat{\sigma}_{16}^{z}+2\hat{\sigma}_{13}^{z}\hat{\sigma}_{17}^{z}-$$
$$2\hat{\sigma}_{13}^{z}\hat{\sigma}_{18}^{z}-2\hat{\sigma}_{13}^{z}\hat{\sigma}_{19}^{z}-2\hat{\sigma}_{13}^{z}\hat{\sigma}_{2}^{z}-2\hat{\sigma}_{13}^{z}\hat{\sigma}_{20}^{z}-2\hat{\sigma}_{13}^{z}\hat{\sigma}_{21}^{z}+2\hat{\sigma}_{13}^{z}\hat{\sigma}_{22}^{z}+2\hat{\sigma}_{13}^{z}\hat{\sigma}_{23}^{z}-2\hat{\sigma}_{13}^{z}\hat{\sigma}_{24}^{z}+2\hat{\sigma}_{13}^{z}\hat{\sigma}_{25}^{z}+2\hat{\sigma}_{13}^{z}\hat{\sigma}_{26}^{z}+$$
$$2\hat{\sigma}_{13}^{z}\hat{\sigma}_{27}^{z}-2\hat{\sigma}_{13}^{z}\hat{\sigma}_{3}^{z}+2\hat{\sigma}_{13}^{z}\hat{\sigma}_{4}^{z}+2\hat{\sigma}_{13}^{z}\hat{\sigma}_{5}^{z}+2\hat{\sigma}_{13}^{z}\hat{\sigma}_{6}^{z}+2\hat{\sigma}_{13}^{z}\hat{\sigma}_{7}^{z}-2\hat{\sigma}_{13}^{z}\hat{\sigma}_{8}^{z}-2\hat{\sigma}_{13}^{z}\hat{\sigma}_{9}^{z}+2\hat{\sigma}_{14}^{z}\hat{\sigma}_{15}^{z}-2\hat{\sigma}_{14}^{z}\hat{\sigma}_{16}^{z}-2\hat{\sigma}_{14}^{z}\hat{\sigma}_{17}^{z}+$$
$$2\hat{\sigma}_{14}^{z}\hat{\sigma}_{18}^{z}+2\hat{\sigma}_{14}^{z}\hat{\sigma}_{19}^{z}+2\hat{\sigma}_{14}^{z}\hat{\sigma}_{2}^{z}+2\hat{\sigma}_{14}^{z}\hat{\sigma}_{20}^{z}+2\hat{\sigma}_{14}^{z}\hat{\sigma}_{21}^{z}-2\hat{\sigma}_{14}^{z}\hat{\sigma}_{22}^{z}-2\hat{\sigma}_{14}^{z}\hat{\sigma}_{23}^{z}+2\hat{\sigma}_{14}^{z}\hat{\sigma}_{24}^{z}-2\hat{\sigma}_{14}^{z}\hat{\sigma}_{25}^{z}-2\hat{\sigma}_{14}^{z}\hat{\sigma}_{26}^{z}-$$
$$2\hat{\sigma}_{14}^{z}\hat{\sigma}_{27}^{z}+2\hat{\sigma}_{14}^{z}\hat{\sigma}_{3}^{z}-2\hat{\sigma}_{14}^{z}\hat{\sigma}_{4}^{z}-2\hat{\sigma}_{14}^{z}\hat{\sigma}_{5}^{z}-2\hat{\sigma}_{14}^{z}\hat{\sigma}_{6}^{z}-2\hat{\sigma}_{14}^{z}\hat{\sigma}_{7}^{z}+2\hat{\sigma}_{14}^{z}\hat{\sigma}_{8}^{z}+2\hat{\sigma}_{14}^{z}\hat{\sigma}_{9}^{z}+2\hat{\sigma}_{15}^{z}\hat{\sigma}_{16}^{z}+2\hat{\sigma}_{15}^{z}\hat{\sigma}_{17}^{z}-2\hat{\sigma}_{15}^{z}\hat{\sigma}_{18}^{z}-$$
$$2\hat{\sigma}_{15}^{z}\hat{\sigma}_{19}^{z}-2\hat{\sigma}_{15}^{z}\hat{\sigma}_{2}^{z}-2\hat{\sigma}_{15}^{z}\hat{\sigma}_{20}^{z}-2\hat{\sigma}_{15}^{z}\hat{\sigma}_{21}^{z}+2\hat{\sigma}_{15}^{z}\hat{\sigma}_{22}^{z}+2\hat{\sigma}_{15}^{z}\hat{\sigma}_{23}^{z}-2\hat{\sigma}_{15}^{z}\hat{\sigma}_{24}^{z}+2\hat{\sigma}_{15}^{z}\hat{\sigma}_{25}^{z}+2\hat{\sigma}_{15}^{z}\hat{\sigma}_{26}^{z}+2\hat{\sigma}_{15}^{z}\hat{\sigma}_{27}^{z}-$$
$$2\hat{\sigma}_{15}^{z}\hat{\sigma}_{3}^{z}+2\hat{\sigma}_{15}^{z}\hat{\sigma}_{4}^{z}+2\hat{\sigma}_{15}^{z}\hat{\sigma}_{5}^{z}+2\hat{\sigma}_{15}^{z}\hat{\sigma}_{6}^{z}+2\hat{\sigma}_{15}^{z}\hat{\sigma}_{7}^{z}-2\hat{\sigma}_{15}^{z}\hat{\sigma}_{8}^{z}-2\hat{\sigma}_{15}^{z}\hat{\sigma}_{9}^{z}-2\hat{\sigma}_{16}^{z}\hat{\sigma}_{17}^{z}+2\hat{\sigma}_{16}^{z}\hat{\sigma}_{18}^{z}+2\hat{\sigma}_{16}^{z}\hat{\sigma}_{19}^{z}+2\hat{\sigma}_{16}^{z}\hat{\sigma}_{2}^{z}+$$
$$2\hat{\sigma}_{16}^{z}\hat{\sigma}_{20}^{z}+2\hat{\sigma}_{16}^{z}\hat{\sigma}_{21}^{z}-2\hat{\sigma}_{16}^{z}\hat{\sigma}_{22}^{z}-2\hat{\sigma}_{16}^{z}\hat{\sigma}_{23}^{z}+2\hat{\sigma}_{16}^{z}\hat{\sigma}_{24}^{z}-2\hat{\sigma}_{16}^{z}\hat{\sigma}_{25}^{z}-2\hat{\sigma}_{16}^{z}\hat{\sigma}_{26}^{z}-2\hat{\sigma}_{16}^{z}\hat{\sigma}_{27}^{z}+2\hat{\sigma}_{16}^{z}\hat{\sigma}_{3}^{z}-2\hat{\sigma}_{16}^{z}\hat{\sigma}_{4}^{z}-2\hat{\sigma}_{16}^{z}\hat{\sigma}_{5}^{z}-$$
$$2\hat{\sigma}_{16}^{z}\hat{\sigma}_{6}^{z}-2\hat{\sigma}_{16}^{z}\hat{\sigma}_{7}^{z}+2\hat{\sigma}_{16}^{z}\hat{\sigma}_{8}^{z}+2\hat{\sigma}_{16}^{z}\hat{\sigma}_{9}^{z}+2\hat{\sigma}_{17}^{z}\hat{\sigma}_{18}^{z}+2\hat{\sigma}_{17}^{z}\hat{\sigma}_{19}^{z}+2\hat{\sigma}_{17}^{z}\hat{\sigma}_{2}^{z}+2\hat{\sigma}_{17}^{z}\hat{\sigma}_{20}^{z}+2\hat{\sigma}_{17}^{z}\hat{\sigma}_{21}^{z}-2\hat{\sigma}_{17}^{z}\hat{\sigma}_{22}^{z}-2\hat{\sigma}_{17}^{z}\hat{\sigma}_{23}^{z}+$$

$$2\hat{\sigma}_{17}^{z}\hat{\sigma}_{24}^{z} - 2\hat{\sigma}_{17}^{z}\hat{\sigma}_{25}^{z} - 2\hat{\sigma}_{17}^{z}\hat{\sigma}_{26}^{z} - 2\hat{\sigma}_{17}^{z}\hat{\sigma}_{27}^{z} + 2\hat{\sigma}_{17}^{z}\hat{\sigma}_{3}^{z} - 2\hat{\sigma}_{17}^{z}\hat{\sigma}_{4}^{z} - 2\hat{\sigma}_{17}^{z}\hat{\sigma}_{5}^{z} - 2\hat{\sigma}_{17}^{z}\hat{\sigma}_{6}^{z} - 2\hat{\sigma}_{17}^{z}\hat{\sigma}_{7}^{z} + 2\hat{\sigma}_{17}^{z}\hat{\sigma}_{8}^{z} + 2\hat{\sigma}_{17}^{z}\hat{\sigma}_{9}^{z} -$$

$$2\hat{\sigma}_{18}^{z}\hat{\sigma}_{19}^{z} - 2\hat{\sigma}_{18}^{z}\hat{\sigma}_{2}^{z} - 2\hat{\sigma}_{18}^{z}\hat{\sigma}_{20}^{z} - 2\hat{\sigma}_{18}^{z}\hat{\sigma}_{21}^{z} + 2\hat{\sigma}_{18}^{z}\hat{\sigma}_{22}^{z} + 2\hat{\sigma}_{18}^{z}\hat{\sigma}_{23}^{z} - 2\hat{\sigma}_{18}^{z}\hat{\sigma}_{24}^{z} + 2\hat{\sigma}_{18}^{z}\hat{\sigma}_{25}^{z} + 2\hat{\sigma}_{18}^{z}\hat{\sigma}_{26}^{z} + 2\hat{\sigma}_{18}^{z}\hat{\sigma}_{27}^{z} -$$

$$2\hat{\sigma}_{18}^{z}\hat{\sigma}_{3}^{z} + 2\hat{\sigma}_{18}^{z}\hat{\sigma}_{4}^{z} + 2\hat{\sigma}_{18}^{z}\hat{\sigma}_{5}^{z} + 2\hat{\sigma}_{18}^{z}\hat{\sigma}_{6}^{z} + 2\hat{\sigma}_{18}^{z}\hat{\sigma}_{7}^{z} - 2\hat{\sigma}_{18}^{z}\hat{\sigma}_{8}^{z} - 2\hat{\sigma}_{18}^{z}\hat{\sigma}_{9}^{z} - 2\hat{\sigma}_{19}^{z}\hat{\sigma}_{2}^{z} - 2\hat{\sigma}_{19}^{z}\hat{\sigma}_{20}^{z} - 2\hat{\sigma}_{19}^{z}\hat{\sigma}_{21}^{z} + 2\hat{\sigma}_{19}^{z}\hat{\sigma}_{22}^{z} +$$

$$2\hat{\sigma}_{19}^{z}\hat{\sigma}_{23}^{z} - 2\hat{\sigma}_{19}^{z}\hat{\sigma}_{24}^{z} + 2\hat{\sigma}_{19}^{z}\hat{\sigma}_{25}^{z} + 2\hat{\sigma}_{19}^{z}\hat{\sigma}_{26}^{z} + 2\hat{\sigma}_{19}^{z}\hat{\sigma}_{27}^{z} - 2\hat{\sigma}_{19}^{z}\hat{\sigma}_{3}^{z} + 2\hat{\sigma}_{19}^{z}\hat{\sigma}_{4}^{z} + 2\hat{\sigma}_{19}^{z}\hat{\sigma}_{5}^{z} + 2\hat{\sigma}_{19}^{z}\hat{\sigma}_{6}^{z} + 2\hat{\sigma}_{19}^{z}\hat{\sigma}_{7}^{z} - 2\hat{\sigma}_{19}^{z}\hat{\sigma}_{8}^{z} -$$

$$2\hat{\sigma}_{19}^{z}\hat{\sigma}_{9}^{z} - 2\hat{\sigma}_{2}^{z}\hat{\sigma}_{20}^{z} - 2\hat{\sigma}_{2}^{z}\hat{\sigma}_{21}^{z} + 2\hat{\sigma}_{2}^{z}\hat{\sigma}_{22}^{z} + 2\hat{\sigma}_{2}^{z}\hat{\sigma}_{23}^{z} - 2\hat{\sigma}_{2}^{z}\hat{\sigma}_{24}^{z} + 2\hat{\sigma}_{2}^{z}\hat{\sigma}_{25}^{z} + 2\hat{\sigma}_{2}^{z}\hat{\sigma}_{26}^{z} + 2\hat{\sigma}_{2}^{z}\hat{\sigma}_{27}^{z} - 2\hat{\sigma}_{2}^{z}\hat{\sigma}_{3}^{z} + 2\hat{\sigma}_{2}^{z}\hat{\sigma}_{4}^{z} +$$

$$2\hat{\sigma}_{2}^{z}\hat{\sigma}_{5}^{z} + 2\hat{\sigma}_{2}^{z}\hat{\sigma}_{6}^{z} + 2\hat{\sigma}_{2}^{z}\hat{\sigma}_{7}^{z} - 2\hat{\sigma}_{2}^{z}\hat{\sigma}_{8}^{z} - 2\hat{\sigma}_{2}^{z}\hat{\sigma}_{9}^{z} - 2\hat{\sigma}_{20}^{z}\hat{\sigma}_{21}^{z} + 2\hat{\sigma}_{20}^{z}\hat{\sigma}_{22}^{z} + 2\hat{\sigma}_{20}^{z}\hat{\sigma}_{23}^{z} - 2\hat{\sigma}_{20}^{z}\hat{\sigma}_{24}^{z} + 2\hat{\sigma}_{20}^{z}\hat{\sigma}_{25}^{z} + 2\hat{\sigma}_{20}^{z}\hat{\sigma}_{26}^{z} +$$

$$2\hat{\sigma}_{20}^{z}\hat{\sigma}_{27}^{z} - 2\hat{\sigma}_{20}^{z}\hat{\sigma}_{3}^{z} + 2\hat{\sigma}_{20}^{z}\hat{\sigma}_{4}^{z} + 2\hat{\sigma}_{20}^{z}\hat{\sigma}_{5}^{z} + 2\hat{\sigma}_{20}^{z}\hat{\sigma}_{6}^{z} + 2\hat{\sigma}_{20}^{z}\hat{\sigma}_{7}^{z} - 2\hat{\sigma}_{20}^{z}\hat{\sigma}_{8}^{z} - 2\hat{\sigma}_{20}^{z}\hat{\sigma}_{9}^{z} + 2\hat{\sigma}_{21}^{z}\hat{\sigma}_{22}^{z} + 2\hat{\sigma}_{21}^{z}\hat{\sigma}_{23}^{z} - 2\hat{\sigma}_{21}^{z}\hat{\sigma}_{24}^{z} +$$

$$2\hat{\sigma}_{21}^{z}\hat{\sigma}_{25}^{z} + 2\hat{\sigma}_{21}^{z}\hat{\sigma}_{26}^{z} + 2\hat{\sigma}_{21}^{z}\hat{\sigma}_{27}^{z} - 2\hat{\sigma}_{21}^{z}\hat{\sigma}_{3}^{z} + 2\hat{\sigma}_{21}^{z}\hat{\sigma}_{4}^{z} + 2\hat{\sigma}_{21}^{z}\hat{\sigma}_{5}^{z} + 2\hat{\sigma}_{21}^{z}\hat{\sigma}_{6}^{z} + 2\hat{\sigma}_{21}^{z}\hat{\sigma}_{7}^{z} - 2\hat{\sigma}_{21}^{z}\hat{\sigma}_{8}^{z} - 2\hat{\sigma}_{21}^{z}\hat{\sigma}_{9}^{z} - 2\hat{\sigma}_{22}^{z}\hat{\sigma}_{23}^{z} +$$

$$2\hat{\sigma}_{22}^{z}\hat{\sigma}_{24}^{z} - 2\hat{\sigma}_{22}^{z}\hat{\sigma}_{25}^{z} - 2\hat{\sigma}_{22}^{z}\hat{\sigma}_{26}^{z} - 2\hat{\sigma}_{22}^{z}\hat{\sigma}_{27}^{z} + 2\hat{\sigma}_{22}^{z}\hat{\sigma}_{3}^{z} - 2\hat{\sigma}_{22}^{z}\hat{\sigma}_{4}^{z} - 2\hat{\sigma}_{22}^{z}\hat{\sigma}_{5}^{z} - 2\hat{\sigma}_{22}^{z}\hat{\sigma}_{6}^{z} - 2\hat{\sigma}_{22}^{z}\hat{\sigma}_{7}^{z} + 2\hat{\sigma}_{22}^{z}\hat{\sigma}_{8}^{z} + 2\hat{\sigma}_{22}^{z}\hat{\sigma}_{9}^{z} +$$

$$2\hat{\sigma}_{23}^{z}\hat{\sigma}_{24}^{z} - 2\hat{\sigma}_{23}^{z}\hat{\sigma}_{25}^{z} - 2\hat{\sigma}_{23}^{z}\hat{\sigma}_{26}^{z} - 2\hat{\sigma}_{23}^{z}\hat{\sigma}_{27}^{z} + 2\hat{\sigma}_{23}^{z}\hat{\sigma}_{3}^{z} - 2\hat{\sigma}_{23}^{z}\hat{\sigma}_{4}^{z} - 2\hat{\sigma}_{23}^{z}\hat{\sigma}_{5}^{z} - 2\hat{\sigma}_{23}^{z}\hat{\sigma}_{6}^{z} - 2\hat{\sigma}_{23}^{z}\hat{\sigma}_{7}^{z} + 2\hat{\sigma}_{23}^{z}\hat{\sigma}_{8}^{z} + 2\hat{\sigma}_{23}^{z}\hat{\sigma}_{9}^{z} +$$

$$2\hat{\sigma}_{24}^{z}\hat{\sigma}_{25}^{z} + 2\hat{\sigma}_{24}^{z}\hat{\sigma}_{26}^{z} + 2\hat{\sigma}_{24}^{z}\hat{\sigma}_{27}^{z} - 2\hat{\sigma}_{24}^{z}\hat{\sigma}_{3}^{z} + 2\hat{\sigma}_{24}^{z}\hat{\sigma}_{4}^{z} + 2\hat{\sigma}_{24}^{z}\hat{\sigma}_{5}^{z} + 2\hat{\sigma}_{24}^{z}\hat{\sigma}_{6}^{z} + 2\hat{\sigma}_{24}^{z}\hat{\sigma}_{7}^{z} - 2\hat{\sigma}_{24}^{z}\hat{\sigma}_{8}^{z} - 2\hat{\sigma}_{24}^{z}\hat{\sigma}_{9}^{z} - 2\hat{\sigma}_{25}^{z}\hat{\sigma}_{26}^{z} -$$

$$2\hat{\sigma}_{25}^{z}\hat{\sigma}_{27}^{z} + 2\hat{\sigma}_{25}^{z}\hat{\sigma}_{3}^{z} - 2\hat{\sigma}_{25}^{z}\hat{\sigma}_{4}^{z} - 2\hat{\sigma}_{25}^{z}\hat{\sigma}_{5}^{z} - 2\hat{\sigma}_{25}^{z}\hat{\sigma}_{6}^{z} - 2\hat{\sigma}_{25}^{z}\hat{\sigma}_{7}^{z} + 2\hat{\sigma}_{25}^{z}\hat{\sigma}_{8}^{z} + 2\hat{\sigma}_{25}^{z}\hat{\sigma}_{9}^{z} - 2\hat{\sigma}_{26}^{z}\hat{\sigma}_{27}^{z} + 2\hat{\sigma}_{26}^{z}\hat{\sigma}_{3}^{z} - 2\hat{\sigma}_{26}^{z}\hat{\sigma}_{4}^{z} -$$

$$2\hat{\sigma}_{26}^{z}\hat{\sigma}_{5}^{z} - 2\hat{\sigma}_{26}^{z}\hat{\sigma}_{6}^{z} - 2\hat{\sigma}_{26}^{z}\hat{\sigma}_{7}^{z} + 2\hat{\sigma}_{26}^{z}\hat{\sigma}_{8}^{z} + 2\hat{\sigma}_{26}^{z}\hat{\sigma}_{9}^{z} + 2\hat{\sigma}_{27}^{z}\hat{\sigma}_{3}^{z} - 2\hat{\sigma}_{27}^{z}\hat{\sigma}_{4}^{z} - 2\hat{\sigma}_{27}^{z}\hat{\sigma}_{5}^{z} - 2\hat{\sigma}_{27}^{z}\hat{\sigma}_{6}^{z} - 2\hat{\sigma}_{27}^{z}\hat{\sigma}_{7}^{z} + 2\hat{\sigma}_{27}^{z}\hat{\sigma}_{8}^{z} +$$

$$2\hat{\sigma}_{27}^{z}\hat{\sigma}_{9}^{z} + 2\hat{\sigma}_{3}^{z}\hat{\sigma}_{4}^{z} + 2\hat{\sigma}_{3}^{z}\hat{\sigma}_{5}^{z} + 2\hat{\sigma}_{3}^{z}\hat{\sigma}_{6}^{z} + 2\hat{\sigma}_{3}^{z}\hat{\sigma}_{7}^{z} - 2\hat{\sigma}_{3}^{z}\hat{\sigma}_{8}^{z} - 2\hat{\sigma}_{3}^{z}\hat{\sigma}_{9}^{z} - 2\hat{\sigma}_{4}^{z}\hat{\sigma}_{5}^{z} - 2\hat{\sigma}_{4}^{z}\hat{\sigma}_{6}^{z} - 2\hat{\sigma}_{4}^{z}\hat{\sigma}_{7}^{z} + 2\hat{\sigma}_{4}^{z}\hat{\sigma}_{8}^{z} +$$

$$2\hat{\sigma}_{4}^{z}\hat{\sigma}_{9}^{z} - 2\hat{\sigma}_{5}^{z}\hat{\sigma}_{6}^{z} - 2\hat{\sigma}_{5}^{z}\hat{\sigma}_{7}^{z} + 2\hat{\sigma}_{5}^{z}\hat{\sigma}_{8}^{z} + 2\hat{\sigma}_{5}^{z}\hat{\sigma}_{9}^{z} - 2\hat{\sigma}_{6}^{z}\hat{\sigma}_{7}^{z} + 2\hat{\sigma}_{6}^{z}\hat{\sigma}_{8}^{z} + 2\hat{\sigma}_{6}^{z}\hat{\sigma}_{9}^{z} + 2\hat{\sigma}_{7}^{z}\hat{\sigma}_{8}^{z} + 2\hat{\sigma}_{7}^{z}\hat{\sigma}_{9}^{z} - 2\hat{\sigma}_{8}^{z}\hat{\sigma}_{9}^{z} + 756$$

REFERENCES

[1] A.B. Suksmono. Finding a Hadamard matrix by simulated annealing of spin vectors. *J. Phys.: Conf. Ser.*, 856(1):012012, 2017.

[2] A.B. Suksmono. Finding a Hadamard matrix by simulated quantum annealing. *Entropy*, 20(2):141, 2018.

[3] J. D. Biamonte. Nonperturbative k-body to two-body commuting conversion Hamiltonians and embedding problem instances into ising spins. *Phys. Rev. A*, 77(5):052331, 2008.

[4] A. Perdomo, C. Truncik, I. Tubert-Brohman, G. Rose, and A. Aspuru-Guzik. Construction of model Hamiltonians for adiabatic quantum computation and its application to finding low-energy conformations of lattice protein models. *Phys. Rev. A*, 78(1):012320, 2008.
